# An Artificial Antibody‐Based Toolbox Accelerates Validation of Hidden Microproteins Encoded by the Dark Genome

**DOI:** 10.1002/advs.202515707

**Published:** 2026-03-03

**Authors:** Hui He, Anqi Zhang, Zhanchen Guo, Qi Li, Song Gao, Wenyan Guan, Ying Li, Jingran Chen, Sijia Sun, Jian He, Zhen Liu

**Affiliations:** ^1^ State Key Laboratory of Analytical Chemistry for Life Science School of Chemistry Nanjing University Nanjing China; ^2^ Department of Pathology The Affiliated Drum Tower Hospital of Nanjing University Medical School Nanjing China; ^3^ Department of Nuclear Medicine The Affiliated Drum Tower Hospital of Nanjing University Medical School Nanjing China

**Keywords:** microproteins, molecularly imprinted polymers, non‐coding RNAs, small open reading frames, surface‐enhanced Raman scattering

## Abstract

Microproteins encoded by small open reading frames are a pivotal blind spot redefining the conventional protein‐coding assumptions. However, the annotation of the “dark proteome” remains time‐ and labor‐consuming due to the limited efficiency, sensitivity, and comprehensiveness of existing validation methods. To address these issues, we developed a comprehensive toolbox called CLAIMID to achieve accelerated and ultrasensitive validation at multiple biological scales. As the core of CLAIMID, molecularly imprinted polymers (MIPs), which are synthesized artificial antibodies toward putative microproteins, provide ultrasensitive and precise annotation in combination with surface‐enhanced Raman scattering (SERS) detection. The excellent specificity, comparable to antibodies, of MIPs enables high anti‐interference against biological matrix. The adaptability of MIPs engineering confers the rigorous validations by CLAIMID at multiple scales (single living cells, cell populations, and tissues) and diverse detection formats (SERS‐based immunoassay and imaging, and mass spectrometric identification). Through CLAIMID, we rapidly confirmed the protein‐level translation of four predicted microproteins—previously supported only by computational or ribosome profiling data—across various cell lines, and further identified three as potential tumor biomarkers, thereby demonstrating its university to putative microproteins. Together, we present an annotation toolbox with unparalleled efficiency, sensitivity, and scalability, moving forward for the advent of intriguing microprotein biology era.

## Graphical abstract

Microproteins are hidden treasures encoded by the “dark proteome” but remain largely underexplored due to the lack of highly efficient tools. We developed a molecularly imprinted polymers (MIPs)‐based toolbox (CLAIMID) to achieve accelerated and ultrasensitive microproteins validation at multiple biological scales (single living cells, cell populations, and tissues). Through CLAIMID, we validated four novel microproteins and demonstrated three of them as potential tumor biomarkers.

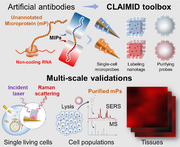

## 1 Introduction

Small open reading frames (sORFs), with the potential to encode microproteins of less than 100 amino acids, were historically dismissed in genome due to annotation prejudices [1, 2]. Owing to the advances in computational biology and sequencing technology [3, 4], emerging evidences supporting active translation of sORFs have ignited a previously hidden field in the proteome. Though ongoing investigative efforts paid, microproteins with limited validation at the protein level represent a large repertoire of poorly studied molecules possibly having crucial physiological roles [5, 6, 7]. Thus, the discovery and validation of novel microproteins is undoubtably of paramount importance, which will revolutionize and complete the proteome.

To redefine the blurry “coding vs noncoding” dichotomy, many characterization approaches, such as mass spectrometry (MS) [8], immunoassay and gene editing [9, 10], have been developed for microprotein validation, despite still suffering from several technical concerns. The tiny size and extremely low abundance of microproteins challenge these methods in detection sensitivity and manipulation generality (e.g. few peptide choices for optimal antigenicity or protease digestion) [11]. Furthermore, the tedious and complicated pre‐ or post‐processing procedures, like flag sequence knock‐in or antibody production, further hampers the annotation progresses [12]. Beyond technological refinement, there is growing consensus that the holistic annotation of one biomolecule is a multilayered challenge. The biology of living organism is by nature multiscale, coordinated by diverse hierarchies that factor into progressively smaller units [13]. A comprehensive view of any biological entity cannot be investigated in isolation from just one or two subsystems [14, 15, 16]. Although a limited portion of microproteins’ subset information in cell populations or tissues has been provided, single living cells, which provide varying niches for molecular complexity and, in doing so, elicit emergent heterogeneous phenotypes at further scales, is an indispensable whereas lost stratification [17, 18]. Altogether, microprotein biology is urgently calling for a sensitive and versatile approach with streamlined procedure and multiscale profiling capability.

**FIGURE 1:**
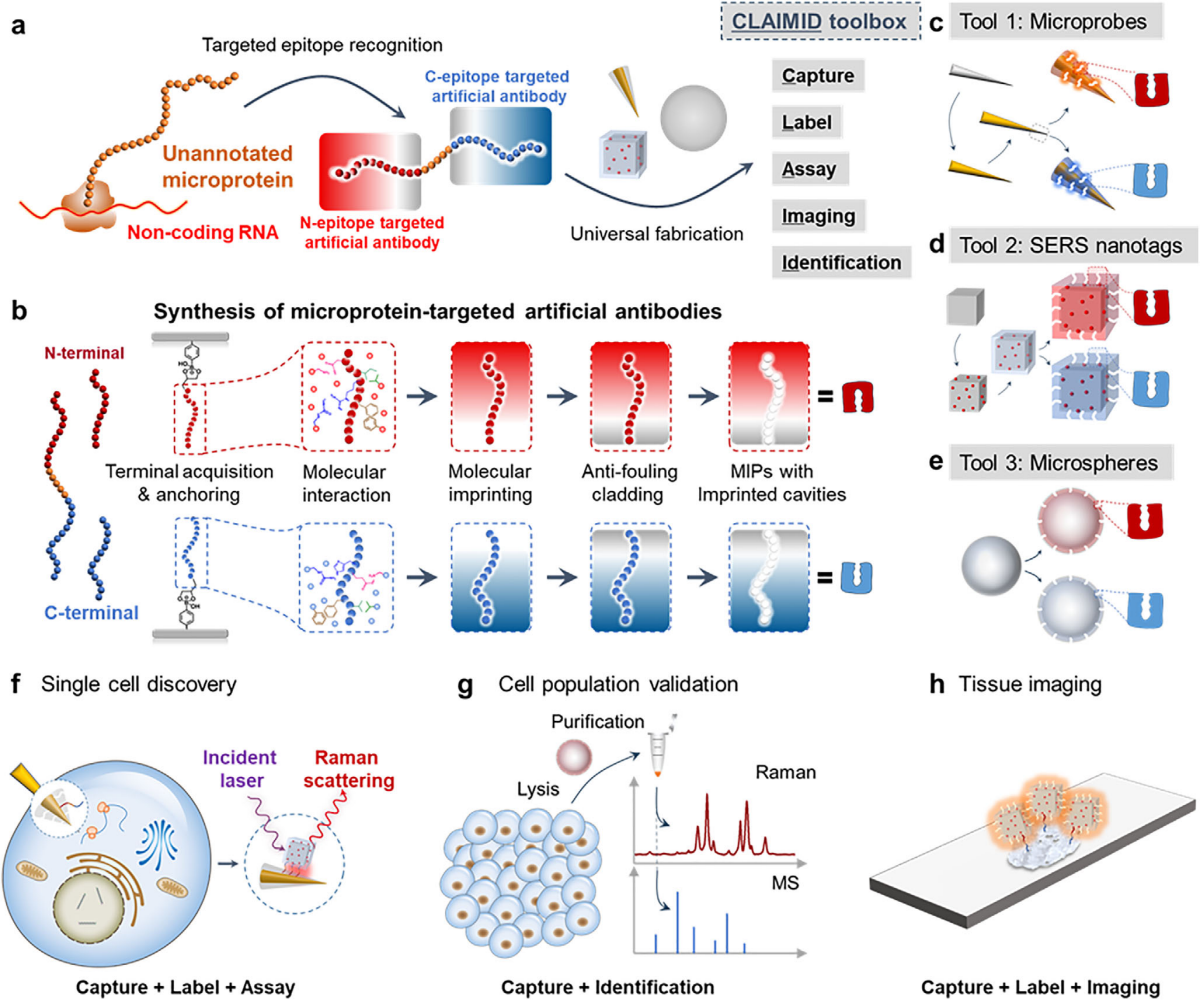
Schematic of probing and validating tumor‐associated microproteins encoded by sORFs using MIP‐based CLAIMID toolbox. (a) Construction of artificial antibody‐based CLAIMID toolbox for microprotein capture, label, assay, imaging, and identification. (b) Production of artificial antibodies toward a selected microprotein. (c) Microprobes in CLAIMID toolbox. (d) Plasmonic nanotags in CLAIMID toolbox. (e) Microspheres in CLAIMID toolbox. (f) Single‐cell assay for detection of microprotein. (g) Detection and identification of microprotein in cell population. (h) Tissue imaging of microprotein by SERS imaging.

To address the aforementioned challenges, we herein present an unprecedented microprotein validation toolbox termed CLAIMID, whose name is derived from its multi‐functions including capture, labeling, assay, imaging, and identification. The core functionality of CLAIMID for precise microprotein recognition hinges on molecularly imprinted polymers (MIPs), which are artificial antibodies synthesized via copolymerization of multiple functional monomers in the presence of a template (artificial antigen) [19, 20]. Once the specific recognition toward target microprotein occurs, CLAIMID will output ultrasensitive surface‐enhanced Raman scattering (SERS) [21] signals confirming its presence at multiple levels. The MIPs, offering tailored recognition sites for each characteristic template [22, 23, 24], can be theoretically designed for specifically recognizing any putative microproteins. Not only the wide applicability for microproteins, but the adaptability of CLAIMID also comes from the flexibility of MIPs engineering. Through MIPs fabricated on materials with diverse formats, the applications of CLAIMID range from single living cells to cell populations, and eventually expanded to macroscale tissue, resulting in rigorous conclusion from multiple‐method validation (SERS‐based assay and imaging, and MS identification). The schematic for CLAIMID is illustrated in Figure 1. Besides precise annotations, the multilevel charting via CLAIMID also paves a new way for the elucidation of cancer‐related microproteins. Using CLAIMID, we validated four new microproteins and pinpointed three of them differentially expressed in tumors, enriching the potential biomarker catalogs across scales. Together, the proposed CLAIMID toolbox can fulfil the challenging exploration of microproteins, possessing several unique advantages such as target universality, customized design, easy‐preparation, ultrahigh sensitivity, and multiscale characterization. We believe that the joint analyses via CLAIMID will draw the panoramic views of novel microproteins and eventually provide thorough understandings for their functional assignments.

## 2 Results and Discussion

### 2.1 Rational Selection of Putative Microproteins

According to the genomic locations, sORFs are categorized as those embedded within the non‐coding RNAs and within the untranslated regions (UTRs) of mRNAs [25]. To broadly explore multiscale landscapes of novel microproteins, we selected the protein‐coding‐potential sORFs in both categories based on ribosome sequencing data [3, 26, 27, 28, 29, 30, 31]. The translations from long non‐coding RNAs (lncRNAs) are an important source of microproteins, from which we chose two as follows. One is lncRNA LINC00961 that has been reported to encode a microprotein namely SPAR via a noncanonical ORF (ORF7 in Figure S1a) [32, 33]. Considering the random ribosome scanning [34], other three sORFs in LINC00961, including ORF5, ORF6, and ORF3, were also selected to explore the possible existence of new microproteins (mP2, mP3, and mP4, respectively). The previously validated ORF7‐encoded SPAR (mP1) was used as a positive control for characterizing the proposed toolbox. Another lncRNA, EBLN3P, was selected due to ribosome sequencing reads at ORF1 [3], suggesting the possible translation of presumable microprotein mP5 (Figure S1b). For the UTR‐associated category, upstream ORFs (uORFs) found in the 5’ UTR of protein‐coding gene represent a functionally significant group. We focused on two canonical genes, ZDHHC4 and RNF145, both containing ribosome‐interacting uORFs (Figure S1c,d) [3], as models to provide accurate annotations at protein‐level. The two putative microproteins were named as mP6 and mP7, respectively. More importantly, all of these genes have an association with disease progression in a variety of cancers (Figure S2), hinting at the possible physiological functions of selected microproteins. Together with rational considerations, we chose seven potential microproteins for further validation. Detailed information of these microproteins is given in Table S1.

### 2.2 Synthesis and Characterization of Artificial Antibodies Toward Microprotein Candidates

**FIGURE 2:**
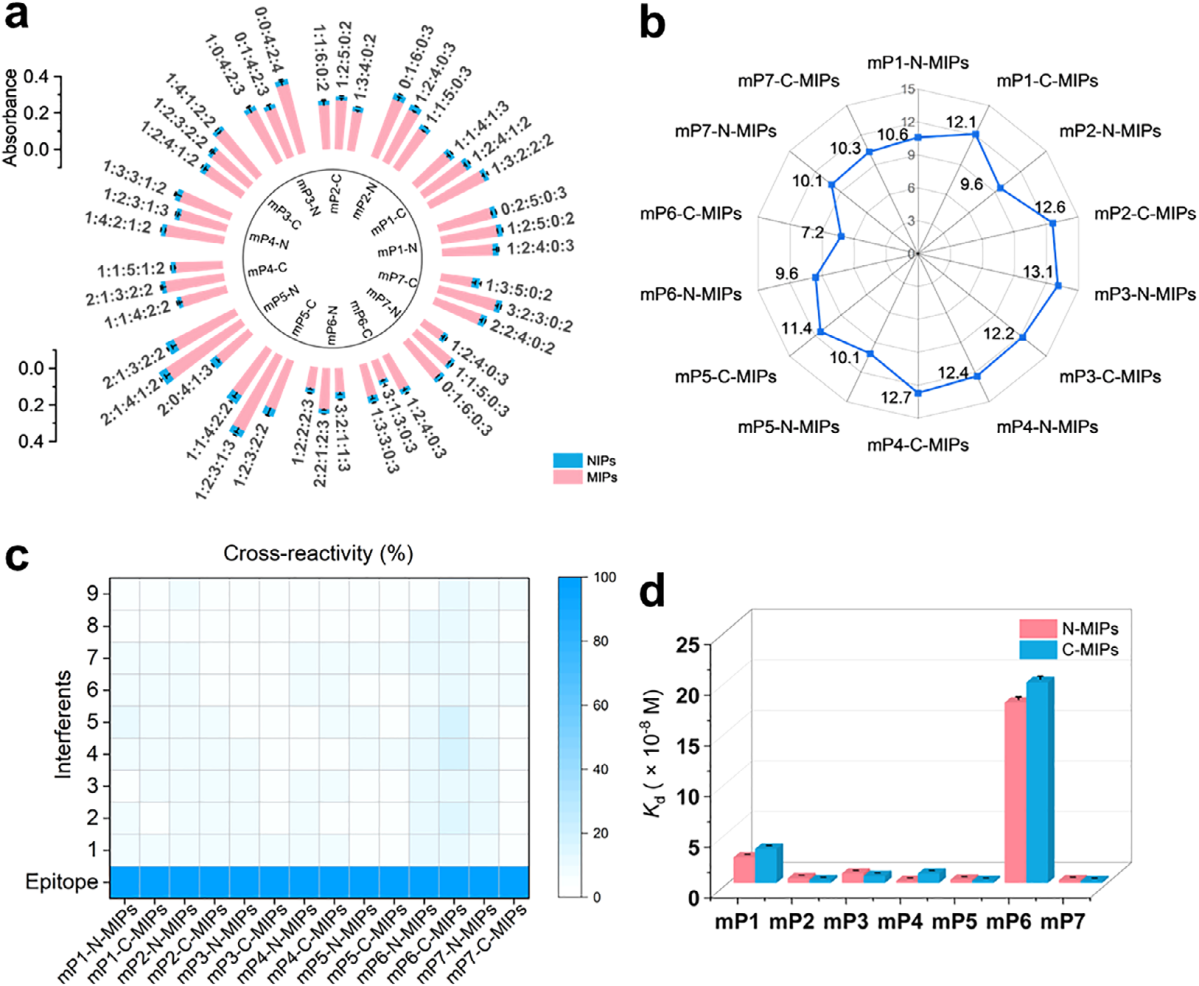
Performance characterization of MIP‐based specific recognition of selected microproteins. (a) Imprinting condition optimization of seven selected microproteins. The ratio values of monomers obey the order of aminopropyltriethoxysilane (APTES), 3‐ureidopropyltriethoxysilane (UPTES), isobutyltriethoxysilane (IBTES), benzyltriethoxysilane (BnTES), and tetraethoxysilane (TEOS). Mean ± SEM, *n* = 3 independent experiments. (b) The imprinting factors of optimized MIPs toward both N‐ and C‐terminal epitopes of the seven microproteins. (c) Cross‐reactivity tests for all of MIPs. Mean ± SEM, *n* = 3 independent experiments, specific interferents see supporting information. (d) Dissociation constants of all of MIPs. The average *K*_d_ values were calculated among four parallel tests.

The multi‐functions of CLAIMID are underpinned by molecular recognition, a fundamental characteristic of biochemical systems covering broad spectrums of orchestrated phenomena between molecules. Working as artificial antibodies with rival performances, MIPs can provide the complementary sites in size, shape, and functional groups for microproteins’ recognitions [35]. This superior recognitive property of MIPs places themselves at the central node of CLAIMID toolbox. Through a state‐of‐art molecular imprinting approach, namely boronate‐affinity oriented surface imprinting and cladding (BOSIC) [24], a set of MIPs for targeting all the putative microproteins were synthesized. The BOSIC approach, with the involvement of four major steps, template immobilization, imprinting, cladding, and template removal, makes excellent selectivity and affinity simultaneously available. Two terminal peptides with 12 amino acid residues were selected as the templates, allowing characteristic epitopes for each targeted microprotein and recognitive homogeneity for corresponding imprinted materials (Figure S3). All of terminal epitopes (except for mP6) exhibited unfolded and flexible conformations in the AlphaFold‐predicted microproteins’ structures [36], demonstrating that they could be easily to be designed for molecular imprinting and subsequent recognition (Figure S4). Using imprinting factor (IF) as a criterion for imprinting effect evaluation, we optimized the monomer ratio of polymerization reaction system to provide the complementary recognition ligands in the imprinted cavities (Figure 2a). The IF value, defined by the ratio of epitope captured by a MIP over that by a corresponding non‐imprinted polymer (NIP), has been widely acknowledged to be correlated with the recognition ability of MIPs. The NIPs were synthesized via identical procedure expect for no template. Thus, the yielded MIPs with high IF values (generally > 9) could efficiently recognize corresponding terminal epitopes of microproteins (Figure 2b and Figures S5–S11). High IF values also brought about lower cross‐reactivity (<10% except for mP6) and dissociation constants (ca. 10^−9^ M except for mP6) (Figure 2c,d and Figures S5–S11). The powerful capturing ability of the optimal MIPs paves the foundation of CLAIMID toolbox. Compared with conventional antibody production, out method exhibited unique advantages. For unstudied microproteins, the whole production takes only 1 week to acquire desired artificial antibodies, while the production of conventional antibodies require at least 1 month. Without intact antigen, the terminal epitope (ca. 12aa) was enough to produce the artificial antibody, avoiding the conjugation of the microprotein to carrier protein and following animal immune‐stimulation, expression, and purification. Notably, due to the generality of the proposed polymerization reaction, our prepared artificial antibodies can be rationally engineered on multiscale and multi‐material structures, actualizing the following functions such as microprotein capture, labeling, imaging, identification, and so on.

### 2.3 Expression Profiles of Microprotein Candidates in Single Living Cells

**FIGURE 3:**
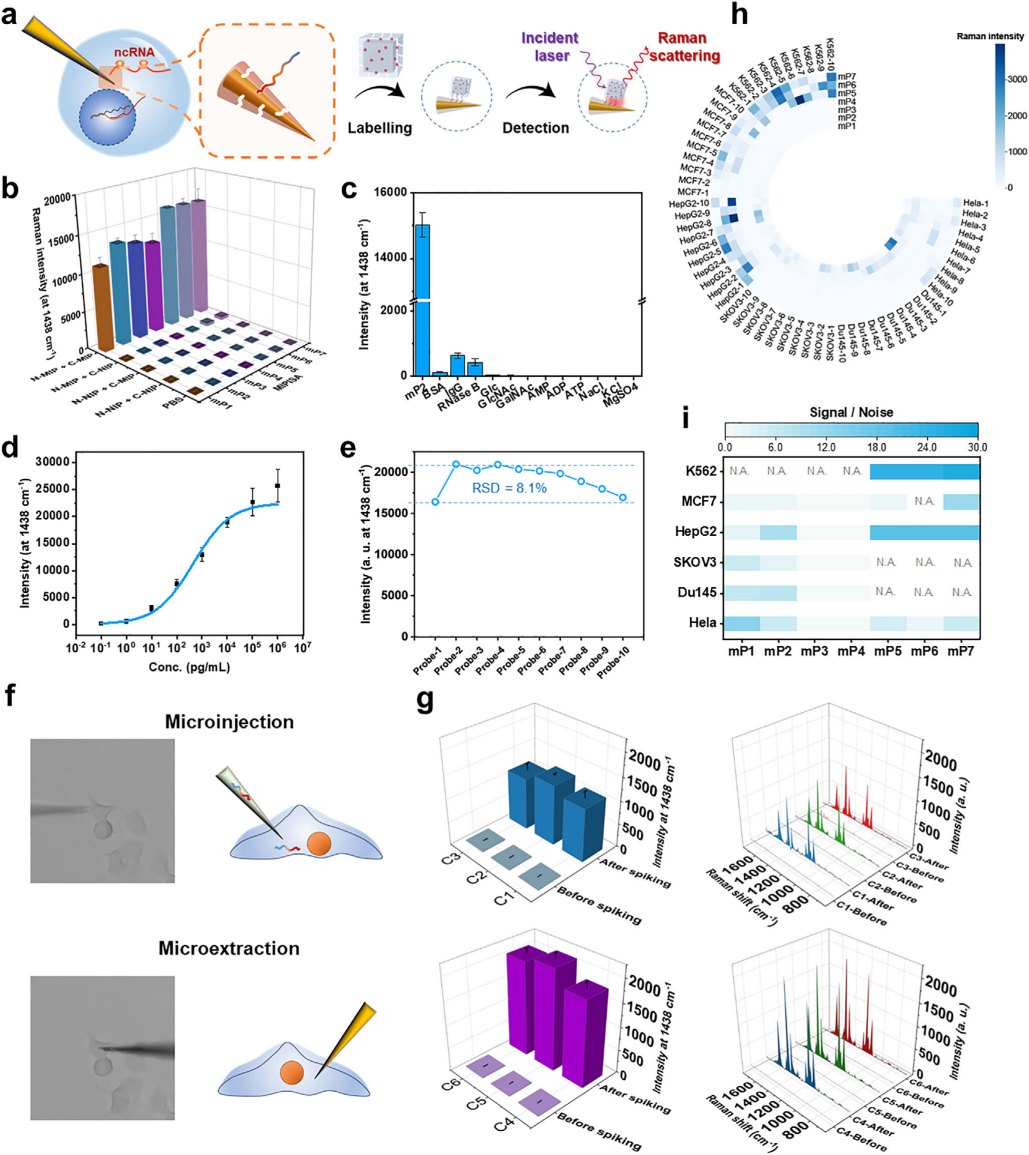
**Expression profiles of microproteins candidates in single living cells**. (a) Schematic of MIPs‐based PISA for microprotein detection in single living cells. (b) Raman intensities for five different combinations of analytes and single‐cell analytical materials (c) Cross‐reactivity of dual recognition for mP2. (d) The binding isotherm for mP2 via CLAIMID. (e) Reproducibility of MIP‐based PISA for mP2 using ten microprobes. (f) Microscopic image and schematic of spike‐and‐test by the aid of our home‐built micromanipulation platform. (g) Raman intensities of PISA and the corresponding spectra for spiked and non‐spiked single living MDA‐MB‐231 cells. C1‐C3, randomly selected single living cells for microinjection with ∼20 copies. C4‐C6, randomly selected single living cells for microinjection with ∼200 copies. Data represent mean ± SEM.  All experiments were performed in triplicate. (h) Raman intensities for potential microproteins in single living cells. The six tested cell lines include HeLa, Du145, SKOV3, HepG2, MCF7, and K562. (i) Heatmap of average microprotein expression levels in different cell lines. Data represent mean ± SEM. All experiments were performed in triplicate.

The intricate and interactive arrangements within a cell play vital roles for biomolecular identity. Thus, the annotation of microprotein in a single living cell, preserving their native niches, is of great importance. To achieve this challenging and unattained goal, we applied microprobes and nanotags as the first tool in CLAIMID, which were fabricated with N‐ and C‐terminal epitope‐specific MIPs respectively, for discovering the putative microproteins in single living cells. Inspired by our previous plasmonic immunosandwich assay (PISA) [37, 38, 39, 40, 41, 42], an analytical methodology applicable to both in vitro and single cells, we designed a customizable recognition and plasmonic detection platform as follows. Using a home‐built 3D micro‐manipulating system (Figure S12), we applied the MIP‐based PISA for capturing the unannotated microproteins by a microprobe in single living cells and verifying them through plasmonic nanotag‐labeling and ultrasensitive detection in vitro (Figure 3a). The single‐cell analytical tools were characterized in Figures S13 and S14. To verify the effectiveness of dual terminal recognition, we designed and analyzed five different combinations of analytes and materials. Only when MIPs for two terminals and target microprotein coexisted, the sandwich structure formed resulting in apparent signal readout (Figure 3b). Lacking any of the three elements led to limited signal, which ensured the reliability of signal outputs. The total cross‐reactivity in this dual recognition condition was less than 4% (Figure 3c). As another key performance characteristic, sensitivity was first evaluated by the binding isotherm of representative model mP2. The limitation of detection was found to be as low as 0.34 pM (Figure 3d), guaranteeing further ultrasensitive detection. The final in vitro detection sensitivity was equivalent to ultrasensitive immunoassays like radioimmunoassay or chemiluminescence immunoassay. Further, we performed the single‐cell detection after quantitatively microinjecting mP2 of the single‐molecule level into an individual blank cell that had been confirmed before injection. The detectable Raman responses (S/N > 3) in the case of very few molecules in vivo inferred that CLAIMID exhibited single‐molecule level sensitivity in single living cells (Figure S15). Through near‐field simulation of plasmonic enhancement via finite difference time domain method, the excellent sensitivity could be contributed the superior plasmonic signal amplification of our applied cube‐on‐probe manner that provided about ten‐fold signal enhancement compared with frequently‐used sphere‐on‐probe manner [37, 38] (Figure S16). Such improved signal enhancement greatly ensured the single‐molecule level sensitivity of the PISA used in this study. The reproducibility was also evaluated through performing ten parallel experiments on ten microprobes, separately. As shown in Figure 3e, stable signal readouts were observed, giving an RSD value of 8.1%. The specificity of this method is the primary determinant. In single living cells, we conducted spike‐and‐test experiments to evaluate the applicability and robustness of the proposed method. Given the exceptional sensitivity of this approach, we initially selected single cells that did not express mP5 to establish baseline measurements. As illustrated in Figure 3f,g, we utilized a 3D micro‐manipulation platform, allowing precise insertion of a micro‐syringe into the cytoplasm of the targeted cells. We then injected varying volumes of mP5 solution at two distinct concentrations. Single‐cell PISA was employed to detect the spiked mP5 molecules within the intracellular milieu. The results demonstrated that despite the complexity and interference from the cellular microenvironment, our method was able to successfully detect the spiked mP5 targets. Notably, the sensitivity of the method remained unaffected by the challenging conditions presented by the living cell context. Additionally, we performed similar assays in another cell line (Figure S17), further confirming the versatility and generalizability of the approach. These findings indicate that the method maintains its high specificity, even in the presence of endogenous cellular components, underscoring its potential for precise biomolecular detection in complex biological systems. Together, above findings suggest that the MIPs‐based CLAIMID is a reliable approach for discovery of novel microproteins in single living cells.

By virtue of the satisfied methodological performance, the expressional profiles of these seven microprotein candidates in single living cells were then explored by MIP‐based PISA, as depicted in Figure 3h and Figures S18–S24. For LINC00961, the detection signals of mP1 from several single cells confirmed its expression, which is in line with literature reports for whether high or low expression [32, 33] and also confirms the reliability of our method. As for the other unstudied microproteins derived from the LINC00961, mP2 was found to be present in two cell lines (Du145 and HepG2) while almost no detectable signal for mP3 and mP4 in all tested cell lines. All of mP2, mP3, and mP4 were not detected in MCF7 cells, which are attributed to extremely limited genetic expression [32, 33]. The other three microproteins, mP5 from EBLN3P, mP6 from ZDHHC4, and mP7 from RNF145 produced apparent Raman responses (S/N > 3) in three tested cell lines, namely HepG2, MCF7, and K562, demonstrating their endogenous expressions. Although cellular heterogeneity caused huge expression difference among single cells, ten of cell tests were enough to reflect the real expression status for each microproteins. The final average expression landscapes of microprotein candidates are shown in Figure 3i. Moreover, the PISA method confers us with a subcellular resolution to insight into the microprotein localization in individual cells [15, 43], which is also vital for proteins’ functionalities [44, 45]. Taking mP5 as an example, according to the recorded photographs, we could obtain the subcellular location of the mP5‐epitope‐imprinted probe while testing, thus making the Raman intensities for mP5 in cytoplasm and nucleus distinguishable. As shown in Figure S25, mP5 was found merely in cytoplasm but not in nucleus of a HepG2 cell. Taken together, above results demonstrated that CLAIMID can offer an efficient tool for discovering and positioning sORF‐encoded microproteins in single living cells. We must emphasize that the interpretation and annotation of microproteins in single living cells, as a previously missed perspective reflecting microproteins’ existence in native status without transfection or gene engineering/editing and cell lysis, will provide more new insights for following identification and in‐depth functional exploration. Such more detailed views were obtained based on the proposed CLAIMID toolbox, which has not been reported before.

### 2.4 Affinity Purification and Validation of Microprotein in Cell Populations

**FIGURE 4:**
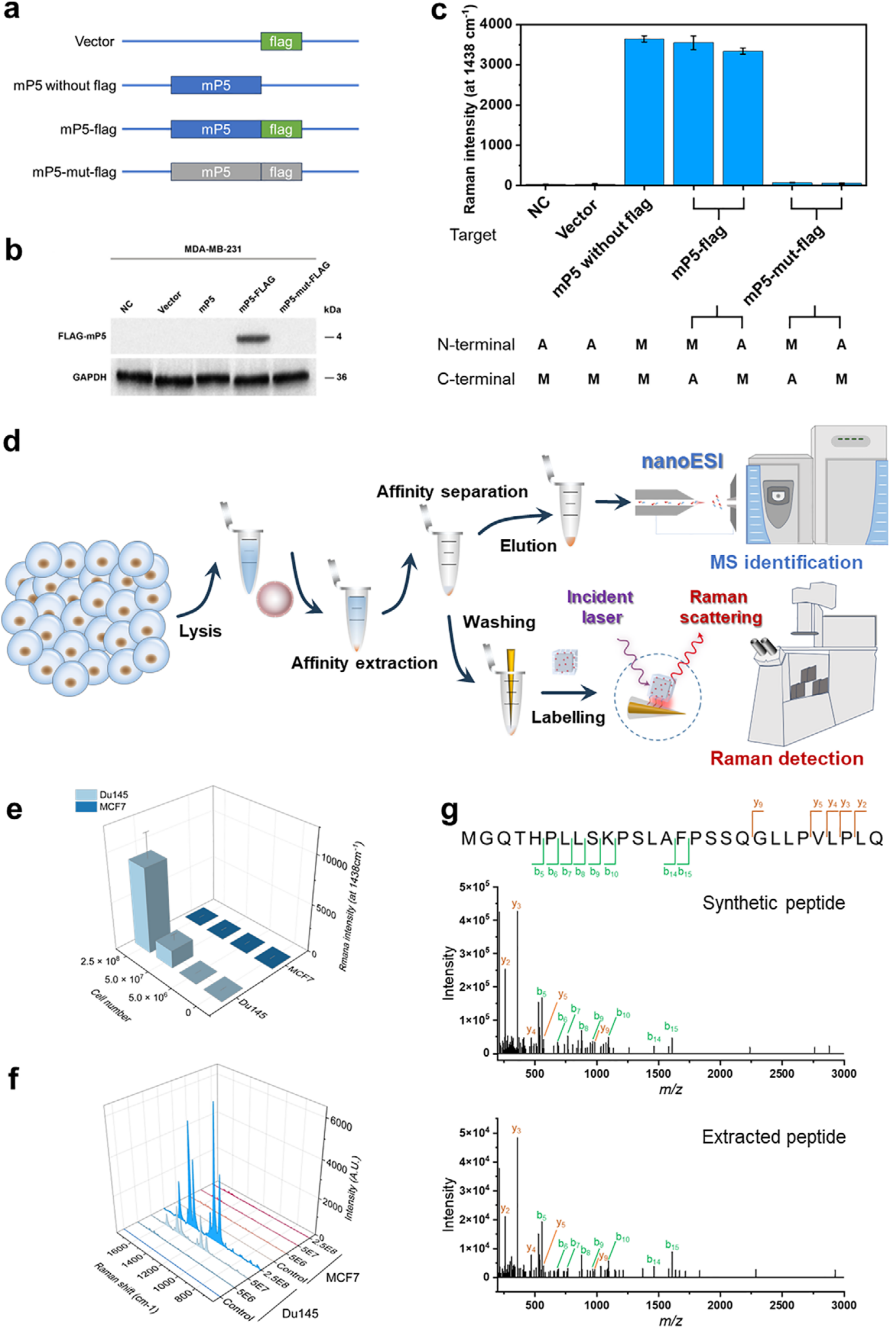
Affinity purification and validation of microprotein in cell populations. (a) Plasmid design for transfection. (b) Western blot analysis results after transfection with different plasmids. (c) Comparison of different groups in (b) by anti‐flag‐based and MIP‐based PISA, separately. A, anti‐flag‐based probe or SERS nanotag. M, MIP‐based probe or SERS nanotag. (d) Schematic of detection and identification of microprotein via CLAIMID‐based affinity purification. Raman intensities of PISA (e) for mP2 in Du145 and MCF7 cell populations consisting of different number of cells and the representative Raman spectra (f). Mean ± SEM, *n* = 3 biologically independent experiments. (g) MS identification of mP2 from lysates of ∼2.5 × 10^8^ Du145 cells. Data represent mean ± SEM. All experiments were performed in triplicate.

The average results produced by cell populations are also critical for microprotein validation. Similar to the single‐cell assay, the specificity of this method in cell lysate is significant. Using mP5 as a representative example, the related cell lysates were used as interferents to evaluate the specificity of the method, and the extremely low endogenous expression of mP5 was reconfirmed. Upon the introduction of exogenous mP5 molecules, their abundance could be clearly and quantitatively measured (Figure S26). These results further demonstrate that the complex cellular components released after cell lysis do not adversely affect the performance of the method. Collectively, spike‐and‐test assays conducted at both the single‐cell and cell‐population levels confirm the high specificity of CLAIMID within complex cellular matrices. More importantly, we performed a series of biological validation experiments to substantiate the specificity and the robustness of the proposed method (Figure 4a). We first overexpressed mP5 via plasmid transfection and subsequently suppressed its expression through start‐codon mutagenesis in two cell lines. As shown in Figure 4b,c and Figure S27, the FLAG‐fused mP5 open reading frame (ORF) was confirmed to encode the microprotein, whereas the corresponding mutated ORF failed to produce mP5, demonstrating that the lncRNA‐derived ORF is translationally competent. To rigorously assess specificity, cell lysates from transfected cells were independently analyzed using anti‐FLAG antibody‐based recognition and MIP‐based recognition. The results show that the MIP‐based probe and nanotag achieve specificity comparable to that of conventional FLAG‐based approaches. Importantly, unlike FLAG fusion strategies, our method does not rely on genetic tagging and is therefore inherently more scalable for microprotein studies, enabling direct detection of native microproteins and facilitating broader application across diverse targets and clinically relevant samples.

Afterward, with mP2 as a model microprotein, we exploited MIP‐based affinity purification for the enrichment and identification of target microprotein in cell lysates. Two cell lines, Du145 cells with mP2 expression and MCF7 cells without mP2 expression, were used as model and control cell lines, respectively. As shown in Figure 4d, after cell lysis and separation by ultracentrifugation, cytosolic proteins were collected for subsequent extraction by MIPs‐engineered microbeads. Once finishing the affinity purification, all of eluents were collected, merged, and concentrated. The resulting eluents were used for PISA analysis and MS validation. For the PISA analysis at cell population level, largely different from the situation in single living cells, Figure 4e,f illustrates that even the Du145 cell number reached up to ∼10^6^, almost no signal was detected. It can be rationalized that conspicuous dilution effect in cell lysate had largely reduced the target microprotein concentration in the cell lysate. As the number of Du145 cells increased to ∼10^8^, significantly enhanced Raman signals were observed but with large fluctuations. The apparent signal fluctuations may reflect the possible short half‐life or rapid degradation of mP2. Such microprotein discovery at single living cell resolution offers two significant advantages over that in cell populations. First, the low‐volume confinement from individual intact cells can ensure the greatest concentration of analyte, which facilitate the capture efficiency, especially for the extremely low‐abundance microproteins. Second, single living cell provides native preservation to avoid cross‐contamination or degradation. For the control cells, almost no signal was detected. By virtue of the proposed affinity purification procedure, the microprotein solutions enriched from ∼2.5 × 10^8^ Du145 cells were subjected to MS identification. The consistency of MS spectra for synthesized and extracted microproteins gave a further supporting of our findings (Figure 4g). The above results suggested that our CLAIMID toolbox enabled not only fast discovery of microproteins in living cells but also purification and identification of microproteins in cell populations. Given the use of identical recognition elements, the discovery results in single living cells are highly credible and comparable with MS identification, but provide improved performance advantages.

### 2.5 Targeted Raman Imaging of Selected Microproteins in Clinical Tissues

**FIGURE 5:**
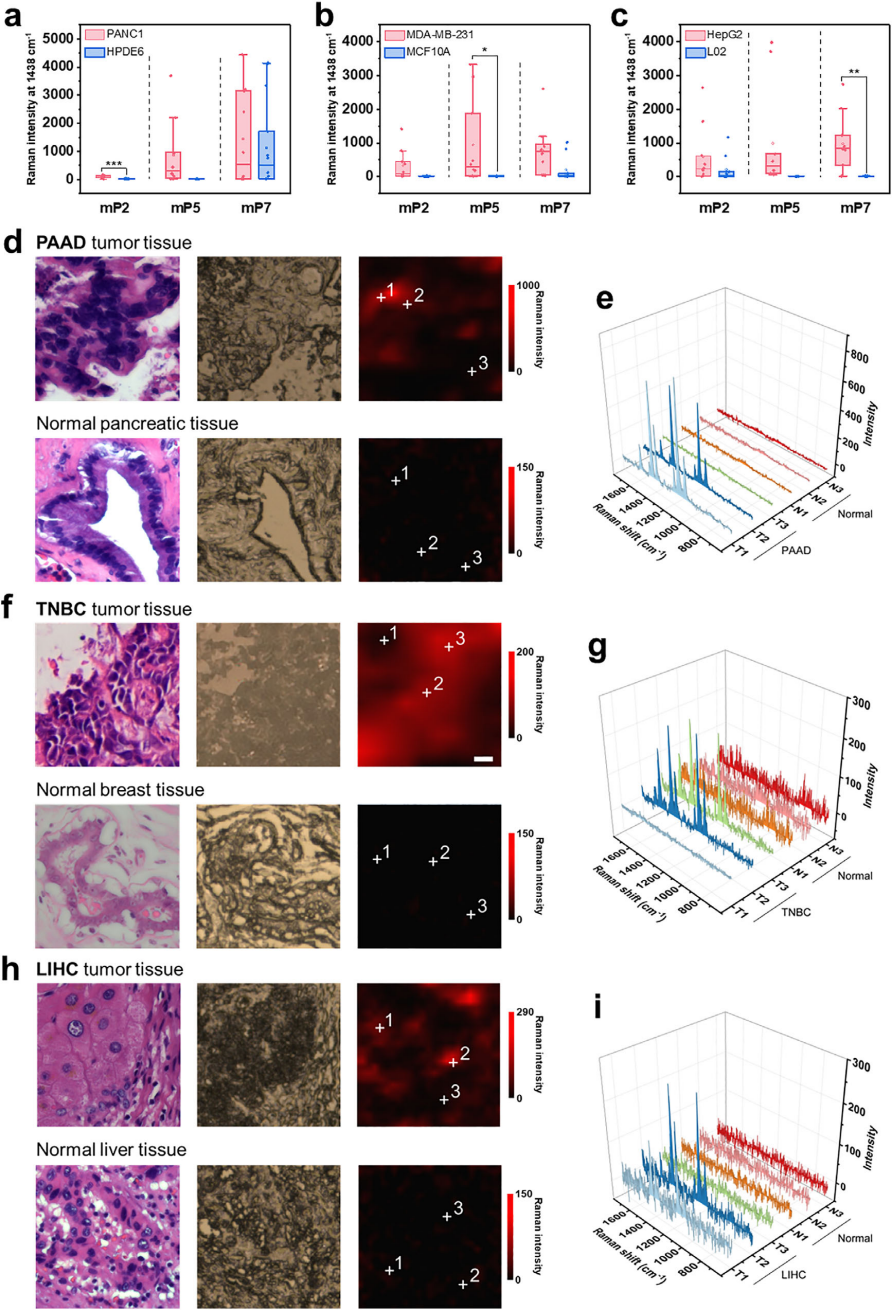
Expression difference of microproteins in single living cells and clinical tissues. (a–c) Detection of mP2, mP5, and mP7 in single living cells (*n*  = 10). The tested cell lines were pancreatic cells (a), mammary cells (b) and hepatocytes (c), respectively. The H&E staining and photographs of clinical tissue slides collected from a pancreatic cancer patient (d), a breast cancer patient (f) and a liver cancer patient (h), and respective SERS imaging of mP2, mP5, and mP7 in these slides. The corresponding three representative Raman spectra of selected positions in the tissues (e, g, and i). The selected positions were indicated in the imaging with cross symbols. Data represent mean ± SEM. No asterisks, not significant; *p < 0.05, **p < 0.01, ***p < 0.001.  All experiments were performed in triplicate.

Understanding the expressional profiling of protein in tissue has played an important role in defining physiological or pathological status and elucidating potential tumor biomarkers [46]. Here, with newly discovered mP2, mP5, and mP7 as representative microproteins, we tentatively explored their potential functions in cancer diagnosis through CLAIMID‐empowered tissue imaging. This was a reasonable choice for potential biomarkers referring to single‐cell experimental results. Apart from the expression profiles, the remarkable expression differences of these microproteins in single cancer and normal cells also pointed out the exciting possibilities of clinical values (Figure 5a–c and Figures S28–S30). As the only three microproteins that showed significant expression differences between corresponding tumor and normal cells, mP2, mP5, and mP7 could be considered as potential biomarkers for pancreatic cancer, breast cancer, and liver cancer, respectively. Complementing this, the bioinformatics results for corresponding RNAs also indicated the functional involvements of respective tumor‐related events, and this trait may also be transmitted to the encoded microproteins (Figures S31–S33). Based on the above observations, we further explored the potential biomarkers using clinical tissue imaging via polyethylene glycol (PEG)‐conjugated MIPs‐plasmonic nanotags. PEGylation of plasmonic nanotag could reduce non‐specific adsorption in clinical tissue samples. As shown in Figure 5d–i, the microscopic images in Raman system were highly matched with the morphology in H&E staining images. After labelling with the nanotags, only tumor tissue slices exhibited obvious SERS imaging signals for microprotein profiling. After testing representative regions of interest, the differential signals in SERS imaging were found to be entirely consistent with the situation in single cells. Together, the highly credible diagnostic information provided from single‐cell results and Raman imaging of tissues jointly revealed the three microproteins tested as possible tumor‐associated biomarkers, laying the foundation of functional explorations in the future.

Notably, different from indispensable cell‐derived xenograft (CDX) or patient‐derived tumor xenograft (PDX) animal model in conventional methods, herein, we realized direct microprotein‐targeted imaging in clinical tissues, showing its potential in clinical application. Undoubtedly, the emerging paradigm of microproteins as functional players in tumorigenesis and as a source of cryptic tumor antigens, as highlighted in recent works [3, 11]. Compared with the previous works in Table S2, here the identification of mP2, mP5, and mP7 as differentially expressed in specific cancers aligns with the broader search for cancer‐type‐specific microprotein biomarkers. The critical need for spatially resolved, sensitive detection tools (like CLAIMID‐enabled tissue imaging) to translate microprotein discoveries from cell lines to clinically actionable biomarkers in complex tumor biopsies. We reference studies that underscore the importance of protein‐level validation in tissues for diagnostic and prognostic applications. It is also worth mentioning that so far, we have completed a comprehensive validation of microproteins spanning from single living cells to cell populations, and to tissues. Despite the inherent limitations (e.g., genetic manipulation is prohibitive) of clinical biospecimens, we deemed the use of genetically defined models, such as CDX, PDX animal model or organoids from isogenic engineered cell lines, as a powerful future direction for more definitive validation of microprotein expression. The integration of CLAIMID with these advanced models such as PDXs or organoid cultures would provide a controlled yet physiologically relevant platform to perform definitive genetic perturbation controls, thereby enabling deeper mechanistic validation of candidate microprotein biomarkers and further establishing the utility of the CLAIMID platform in translational microprotein discovery and validation research. We can foresee that the deeper understandings from microprotein characterization in multiple hierarchical structures will finally draw a thorough network for their physical or functional proximities, which is one of the ultimate goals in biological sciences.

## 3 Conclusion

Spurred by the first draft of the human genome, numerous studies over the past decades have decoded the bulk of coding gene annotation, although many deemed noncoding sequences remain unknown. Complementing the genome and proteome landscapes, “knowing unknowns”—validation of sORFs‐encoded microproteins will be a key challenge in the future. Nowadays, we have reached the point at which the field of microproteins, calling for efficient, sensitive, and multiscale investigations, is hindered by technology. To cope with these difficulties, the presented artificial antibodies‐based toolbox CLAIMID has now been applied for microprotein validation with the unprecedented advance in all required qualities. Especially for the characterization in single living cells, completing this missing plot will promise a more comprehensive profiling into the dark proteome. The outstanding specificity of this method rivals the existing antibody‐based methods, ensuring the applicability in various biological matrix. Owing to the rapid and accurate annotation via CLAIMID, four predicted microproteins were validated at protein‐level and three of them were demonstrated to be associated with tumor progressions, including pancreatic cancer, breast cancer, and liver cancer, expanding the size and category of promising biomarker candidates.

Once the validation of a microprotein is finished, the equally cumbersome work also comes in the form of demonstrating a functional relevance. Gene perturbation is the standard strategy to elucidate functions and it can be achieved by misregulation approaches like gene knock‐down [47, 48]. However, there seems to be a contradiction between the importance of gene dosage for proper functional exertion and the extremely low expression of microproteins [49]. Loss‐ or even gain‐of‐function may not be sufficient to trigger an apparent phenotypical change from average outline of cell populations. Besides, considering the intricate bifunctions of some transcripts, the biological outputs from nucleic acid itself or its coding protein need strict distinctions [50]. In comparison of the conventional microprotein validation methods (Table S2), our method shows obvious advantages including accessible generation of artificial antibodies, ultrahigh sensitivity, multiscale validation, fast verification and high throughput operation. Recently, emerging single‐cell proteomics [51] and nanopore sequencing [52] have present wide coverage and high accuracy for polypeptides, nonetheless, cell lysis remains inevitable, which not only complicates the discovery and validation process but also fails to reflect the native status. From these points, our toolbox CLAIMID, offering ultrahigh sensitivity (single‐molecule level), high resolution (single‐cell and even sub‐cell) and direct evidences at protein level, is ideally suited for subsequent functional explorations of the validated microproteins. To enhance the methodology's utility for the community, we have set several guidelines for implementing CLAIMID for novel microprotein validation, as shown in Table S3. Due to the limited scope of this work, detailed biological roles and related mechanisms should be investigated in future studies.

Together, the newly developed microprotein‐validation toolbox CLAIMID, as a fascinating frontier pushing the thresholds of efficiency, sensitivity and scale, will bring transformative insights into microprotein biology.

## 4 Material and Methods

### 4.1 Reagents and Chemicals

Tetraethoxysilane (TEOS), 4‐aminothiophenol (PATP), bovine serum albumin (BSA), ribonuclease A (RNase A), ribonuclease B (RNase B), horseradish peroxidase (HRP), adenosine monophosphate (AMP), adenosine diphosphate (ADP), adenosine triphosphate (ATP), sodium hydrosulfide (Na_2_S), polyvinylpyrrolidone (PVP, Mw = ca. 55 000) and silver nitrate (AgNO_3_) were purchased from Sigma‐Aldrich (St. Louis, MO, USA). Adenosine, deoxyadenosine, glucose (Glc), galactose (Gal), N‐acetylglucosamine (GlcNAc), 3‐aminopropyltriethoxysilane (APTES), 3‐ureidopropyltriethoxysilane (UPTES), isobutyltriethoxysilane (IBTES), benzyltriethoxysilane (BnTES), 2,4‐difluoro‐3‐formyl‐phenylboronic acid (DFFPBA), and sodium cyanoborohydride (NaCNBH_3_) were purchased from J&K Scientific (Shanghai, China). HAuCl_4_ • 4H_2_O, acetone, and acetonitrile (ACN) were purchased from Macklin (Shanghai, China). Trisodium citrate, potassium bicarbonate (KHCO_3_), glacial acetic acid (HAc), hydrochloric acid (HCl, 36%, v/v), trisodium citrate, sodium hydroxide (NaOH), sodium chloride (NaCl), potassium chloride (KCl), magnesium sulfate (MgSO_4_), glycine, glycerol, methanol and isopropanol were purchased from Nanjing Reagent Company (Nanjing, China). Immunoglobulin G (IgG) was purchased from Beyotime Biotechnology (Shanghai, China). Epitope peptides, including MGAKAPRGPKVA, PVPSAQDFWTFM, MLHSKLLFSSQI, IFKEFGLKGAKL, MYILLWAFPNLI, ENKSHRKYNMFY, MGQTHPLLSKPS, SSQGLLPVLPLQ, MSGSYWSCQAHT, NVGKRNARAAG, MYVTDPESPAAW, LPSVSPAELWN, MVTVRAGPLPLP, HPRLGAEEPPL and corresponding glycated peptides, including Fru‐KMGAKAPRGPKVA, PVPSAQDFWTFMK‐Fru, Fuc‐KMLHSKLLFSSQI, IFKEFGLKGAKLK‐Fru, Fru‐KMYILLWAFPNLI, ENKSHRKYNMFYK‐Fru, Fru‐KMGQTHPLLSKPS, SSQGLLPVLPLQK‐Fru, Fru‐MSGSYWSCQAHT, NVGKRNARAAGK‐Fru, Fru‐MYVTDPESPAAW, LPSVSPAELWNK‐Fru, Fru‐MVTVRAGPLPLP and HPRLGAEEPPLK‐Fru with purity above 98% were obtained by Top‐Peptide Biotechnology (Shanghai, China). Human cervical cancer cell line HeLa (RRID:CVCL_0030), human prostate cancer cell line Du145 (RRID:CVCL_0105), human ovarian cancer cell line SKOV3 (RRID:CVCL_0532), human breast cancer cell line MCF7 (RRID:CVCL_0031) and human hepatoma cell line HepG2 (RRID:CVCL_0027) were obtained from KeySen Biotech (Nanjing, China) in September 2021. Human erythromyeloblastoid leukemia cell line K562 (RRID:CVCL_0004) was obtained from KeySen Biotech (Nanjing, China) in August 2022. All cell lines were cultured according to the supplier's recommendations. Routine mycoplasma contamination tests were performed using a PureLink Genomic DNA Mini Kit (Life K182001) for genomic DNA extraction, a PowerPlex18D system (Promega DC1802) for amplification and an ABI 3500 Genetic Analyzer (Life 3500) for identification. All cell lines were confirmed to be contamination free before use. Saline buffered solution for cell culture (1 × PBS), parenzyme cell digestion solution (containing 0.25% tryptase and 0.02% EDTA), Dulbecco Modified Eagle Medium (DMEM, containing 4.5 mg/mL glucose, 80 U/mL penicillin and 0.08 mg/mL streptomycin), McCoy's 5A medium (5A, containing 0.219 mg/mL glutamine, 80 U/mL penicillin and 0.08 mg/mL streptomycin), Roswell Park Memorial Institute 1640 medium (RPMI‐1640, containing 2.0 mg/mL D‐glucose, 0.3 mg/mL glutamine, 2.0 mg/mL NaHCO_3_, 80 U/mL penicillin and 0.08 mg/mL streptomycin), dimethyl sulfoxide (DMSO), cell freezing medium and cell lysis kit were purchased from KeyGen Biotech (Nanjing, China). Fetal bovine serum (FBS) was purchased from Gibco (Life Technologies, Australia). Trypsin (15090046; GIBCO, Invitrogen Corporation, NY, USA); PVDF membranes (0.2 µm; ISEQ00010; Millipore, Schwalbach, Germany); StarSignal Western Protein Marker (10–200 kDa; M227‐01; GenStar, Beijing, China); Ponceau S (97063‐650), Tween‐20 (97063‐872), and PMSF (97064‐898) (Amresco, VWR International, OH, USA); Western blot stripping buffer (ZN1923; Biolab, Beijing, China); 30% acrylamide solution (ST003), ammonium persulfate substitute (APS substitute; ST005), 1.0 mol L^−^^1^ Tris‐HCl (pH 6.8; ST768), and 1.5 mol L^−^^1^ Tris‐HCl (pH 8.8; ST789) (Beyotime, Shanghai, China); BCA protein assay kit (KGB2101) and RIPA lysis buffer (KGB5203) (KeyGEN BioTECH, Nanjing, China); loading buffer (WB2001; NCM Biotech, Suzhou, China); 10% SDS (BL517A; Biosharp, Hefei, China); TEMED (AR1165; BOSTER, Wuhan, China); anhydrous ethanol (10009257) and methanol (10014118) (SCRC, Shanghai, China); transfer buffer powder (G2017), electrophoresis buffer powder (G2018), and TBST buffer powder (G0001) (Servicebio, Wuhan, China); enhanced chemiluminescence (ECL) substrate (32209; Thermo Fisher Scientific, Pittsburgh, PA, USA).

The plasmids pLNCRNA‐FLAG, pLNCRNA‐ORF, pLNCRNA‐ORF‐FLAG, and pLNCRNA‐ORF‐mut(ΔATG)‐FLAG were constructed by Wuhan Luting Biotechnology Co., Ltd. (Wuhan, China). Opti‐MEM medium (31985070) and Lipofectamine 2000 transfection reagent (11668019) were purchased from Thermo Fisher Scientific (Pittsburgh, PA, USA). Anti‐FLAG antibody (ab205606), anti‐GAPDH antibody (ab181602), and HRP‐conjugated goat anti‐rabbit IgG (ab6721) were obtained from Abcam (Cambridge, UK). Borosilicate glass capillaries of 0.58 mm i.d. and 1.0 mm o.d. were purchased from DL Nature Gene Life Sciences (Shanghai, China). All of reagents used were of analytical grade or higher. HPLC‐grade anhydrous ethanol was purchased from Macklin Inc. (Shanghai, China). Water used in all the experiments was purified by a Milli‐Q Advantage A10 water purification system (Millipore, Milford, MA, USA).

### 4.2 Instruments

MIP‐based PISA was performed on confocal micro‐Raman spectrometry instrument (XploRA INV, Horiba) equipped with a high‐resolution grating with 1200 grooves/mm, additional band‐pass filter optics, and a CCD camera. Raman spectra were acquired using a 638 nm excitation laser with 1 s of integration time and three times of accumulation. The laser was focused onto the tip of microextraction probe by using a ×40 objective (N.A. 0.6), providing a spatial resolution of ca. 1 µm^2^. Raman imaging was performed using a 638 nm excitation laser with 1 s of integration time and one time of accumulation. The laser power at 638 nm was fixed as 28 mW and the filter was set as 10%. Before detection, wavelength calibration was auto‐performed by measuring silicon wafers through a×40 objective, evaluating the first‐order phonon band of Si at 520 cm^−1^. Each detection was repeated three times on the microprobe. Each spectrum was corrected by baseline. The microprobes used in this work were fabricated by tapering 1.0 mm core‐diameter borosilicate glass capillaries using a P‐2000 pipette puller (Sutter Instrument, Novato, CA, USA). A 3D manipulator (Eppendorf, Germany) equipped on an inverted microscope (Nikon, Japan) was used to precisely insert extraction microprobes into single living cells under investigation. A FemtoJet 4i microinjector (Eppendorf, Germany) was used to precisely inject a minute volume of protein solution into single living cells under investigation. Absorbance of test solutions was investigated on a Nanodrop One (Thermofisher, USA). Western blot system (Criterion electrophoresis cell and Trans‐Blot transfer cell; Bio‐Rad); chemiluminescence imaging system (Tanon 5200; Tanon, China); orbital shaker (XK‐8; Jiangsu Xinkang Medical Equipment Co., Ltd., China). The affinity experiments were carried out on an Octet RED96 instrument (Molecular Devices, USA). Transmission electron microscopy (TEM) characterization was performed on a JEM‐2800 system (JEOL, Tokyo, Japan). Scanning electron microscopy (SEM) characterization was performed on a FE‐SEM 7800 instrument (JEOL, Tokyo, Japan). Mass spectrometric analysis was performed on a Q Exactive mass spectrometer (Thermofisher, USA) via nano‐electrospray source (Thermofisher, USA).

### 4.3 RNA Structural Prediction and Analysis

To obtain the structural information of ncRNAs, secondary structures of these RNA sequences were predicted on a web‐based database, namely RNAfold server (http://rna.tbi.univie.ac.at/cgi‐bin/RNAWebSuite/RNAfold.cgi).

### 4.4 Prediction of Opening Reading Frames and Potential Microproteins

To obtain the sequence information of potential microproteins encoded by ncRNAs, opening reading frames (ORFs) were screened from a web‐based database, namely NCBI ORFfinder (https://www.ncbi.nlm.nih.gov/orffinder/). The start codon was set as AUG. The minimum ORF length was set as 75 nt.

### 4.5 Protein Structural Prediction

The predictive structures of microproteins were performed locally using the open source AlphaFold2 repository [36], where the model with a highest residue‐level confidence (pLDDT) was selected of all five predicted TM‐score monomer models. The input included the query sequence and MSA from MMseqs2 [53], with a maximum template date before Jan first, 2022. No additional refinement was performed on the models. The outputs conclude the 3D coordinates of the structures and per‐residue confidence metrics called pLDDT values.

### 4.6 Bioinformatic Analysis

All of the TCGA samples’ data can be found on the TCGA website (https://portal.gdc.cancer.gov/, accessed on 23 January 2024). Using the DESeq2 package in R, we conducted the differential expression genes (DEGs) analysis of TCGA samples with corresponding genes in high or low expression states. According to the median, the high and low expression groups were divided. DEGs were defined as |Log2foldChange (LogFC)| > 1 with adjusted *p* values < 0.05. Gene Ontology (GO) enrichment analysis and gene set enrichment analysis (GSEA) for the DEGs was then performed via clusterProfiler package in R. A *p*‐value < 0.05 was set as significant.

### 4.7 Synthesis and Characterization of Artificial Antibodies

The following epitope‐imprinted polymers are employed as artificial antibodies for specific recognition of the corresponding microproteins. The detailed synthesis and characterization procedures are shown as follows.

#### 4.7.1 Preparation of Silica NSs (SiO_2_ NSs)

SiO_2_ NSs were synthesized according to a reported Stöber method with slight modification [54]. Briefly, 27.5 mL anhydrous ethanol, 15 mL pure water, and 5 mL ammonium hydroxide was premixed and stirred at room temperature for 1 min. Then, a mixture of ethanol (45 mL) and TEOS (4.5 mL) was rapidly added one shot. The mixture was kept severely stirred at room temperature for 15 h. Finally, the resulting NSs were washed with anhydrous ethanol five times and dried under vacuum to obtain the SiO_2_ NSs.

#### 4.7.2 Preparation of Amino‐Functionalized SiO_2_ NSs (NH_2_‐SiO_2_ NSs)

Amino‐functionalization was performed according to our previous study with slight modification [55]. The obtained SiO_2_ NSs were dispersed into 100 mL of anhydrous ethanol by ultrasonication. Then, 0.5 mL APTES was added drop by drop into the mixture under vigorous stirring. After reaction at room temperature for 24 h, the resulting amino‐functionalized SiO_2_ spheres were collected by centrifugation and washed with anhydrous ethanol five times and dried under vacuum to obtain the NH_2_‐SiO_2_ NSs.

#### 4.7.3 Preparation of Boronic Acid‐Modified SiO_2_ NSs (BA‐SiO_2_ NSs)

Boronic acid functionalization was performed according to our previous study with slight modification [55]. 100 mg NH_2_‐SiO_2_ NSs were added into 50 mL anhydrous ethanol containing 1% (w/w) DFFPBA and 1% (w/w) sodium cyanoborohydride, the mixture was vigorously stirred for 48 h at room temperature. The obtained SiO_2_ NSs were collected by centrifugation and washed with anhydrous ethanol five times and dried under vacuum to obtain the DFFPBA‐SiO_2_ NSs.

#### 4.7.4 Preparation of Epitope‐Imprinted SiO_2_ NSs

The epitope‐imprinted SiO_2_ NSs were synthesized using boronate affinity‐oriented surface imprinting and cladding (BOSIC) strategy [4]. The procedure mainly includes three steps: 1) template immobilization; 2) boronate‐affinity oriented surface imprinting and cladding; 3) template removal. The detailed procedures are as follows.

1) Template immobilization: 100 mg DFFPBA‐SiO_2_ NSs were dispersed into 10 mL of 50 mM ammonium bicarbonate buffer containing 500 mM sodium chloride (pH 8.5) containing 1 mg of glycated epitope peptides, including Fru‐KMGAKAPRGPKVA, PVPSAQDFWTFMK‐Fru, Fuc‐KMLHSKLLFSSQI, IFKEFGLKGAKLK‐Fru, Fru‐KMYILLWAFPNLI, ENKSHRKYNMFYK‐Fru, Fru‐KMGQTHPLLSKPS, SSQGLLPVLPLQK‐Fru, Fru‐MSGSYWSCQAHT, NVGKRNA‐RAAGK‐Fru, Fru‐MYVTDPESPAAW, LPSVSPAELWNK‐Fru, Fru‐MVTVRAGPLPLP and HPRLGAEEPPLK‐Fru. The resulting mixture was kept shaking at room temperature for 3 h. The templated epitope‐immobilized SiO_2_ NSs were collected by centrifuging and washing with the ammonium bicarbonate buffer.
2) BOSIC: The obtained templated epitope‐immobilized SiO_2_ NSs were re‐dispersed into 40 mL of anhydrous ethanol containing 0.7 mL of ammonium hydroxide and 10 mL of pure water. Then 10 mL of anhydrous ethanol containing different molar ratios of APTES, UPTES, IBTES, BnTES, and TEOS (10 mM, total concentration) was added to the suspension and the mixture was gently stirred at room temperature for 45 min. The imprinted SiO_2_ NSs were collected by centrifugation, washed by with ethanol for three times, and re‐dispersed into 40 mL of anhydrous ethanol containing 0.7 mL of ammonium hydroxide and 10 mL of pure water. Subsequently, 10 mL of TEOS anhydrous ethanol (10 mm) was added and the mixture was gently stirred at room temperature for 10 min. The resulting epitope‐imprinted and cladded NSs were collected by centrifuging and washing with anhydrous ethanol.
3) Template removal: To remove the template, the epitope‐imprinted SiO_2_ NSs were dispersed into 10 mL of 0.1 M acetic acid containing 50% (v/v) acetonitrile and shaken for 12 h at room temperature. The obtained epitope‐imprinted SiO_2_ NSs were collected, washed with pure water for three times and then dried at 40°C under vacuum for 24 h.

Non‐imprinted SiO_2_ NSs were prepared using the same procedure except for the absence of template immobilization step. The resulting non‐imprinted SiO_2_ NSs were coated with a silica shell of the same thickness as that of the epitope‐imprinted SiO_2_ NSs.

#### 4.7.5 Characterization of Imprinting Factor

To evaluate imprinting factor (*IF*) that is the key factor for assessing recognition ability of MIP, the synthetic epitope peptides were employed as test compounds. Equivalent epitope‐imprinted SiO_2_ NSs and non‐imprinted SiO_2_ NSs (10 mg) were dispersed into 1 mL of 1 mg/mL of corresponding synthetic peptides dissolved in 50 mM ammonium bicarbonate buffer containing 500 mm sodium chloride (pH 8.5) and then the mixture was shocked on a rotator at room temperature for 2 h. The spheres were collected by centrifugation and rinsed with 50 mm ammonium bicarbonate buffer containing 500 mm sodium chloride (pH 8.5) for three times each. Then the epitope‐imprinted SiO_2_ NSs were dispersed into 0.2 mL of 0.1 M acetic acid contating 50%(v/v) acetonitrile and shaken for 3 h at room temperature. The epitope‐imprinted and non‐imprinted SiO_2_ NSs were precipitated by centrifuging and the absorbance of resulting suspendant was examined in Nanodrop at 214 nm. The measurement was repeated for three times. Based on the absorbance intensity of supernatant, *IF* were calculated according to the following equations:

$$IF=I_{MIP}\;/I_{NIP}$$

where *I_MIP_* and *I_NIP_* were the absorbance intensity using MIP‐coated SiO_2_ NSs and NIP‐coated SiO_2_ NSs.

#### 4.7.6 Selectivity Test

To evaluate the cross‐reactivity of epitope‐imprinted SiO_2_ NSs, we chose other common proteins and non‐target epitopes, including HRP, BSA, RNase A, RNase B, and non‐target synthesized epitopes, as interferences. Equivalent epitope‐imprinted SiO_2_ NSs and non‐imprinted SiO_2_ NSs (10 mg) were dispersed into 1000 µL of 1 mg/mL of each microproteins dissolved in 50 mm ammonium bicarbonate buffer containing 500 mm sodium chloride (pH 8.5) and then the mixture was shocked on a rotator at room temperature for 4 h. The spheres were collected by centrifugation and rinsed with 50 mm ammonium bicarbonate buffer containing 500 mm sodium chloride (pH 8.5) for three times each. Then, the spheres were re‐dispersed into 0.2 mL of 0.1 M HAc aqueous solution and shocked on a rotator at room temperature for 1 h. Afterward, the spheres were completely collected by centrifugation. Finally, the resulting supernatant was subjected to UV detection with the wavelength at 214 nm. The measurement was repeated for three times.

#### 4.7.7 Affinity Evaluation

To examine the affinity of epitope‐imprinted SiO_2_ NSs with terminal 12aa‐peptides (mP1) or microproteins (mP2‐mP7), the imprinted SiO_2_ NSs was loaded at 100 µg/mL in kinetics buffer (1 × PBS) for 5 min onto aminopropylsilane (APS) biosensors. Association of target peptides or microproteins at four different concentrations was performed in kinetics buffer solution (0.2‰ Tween‐20 in 1× PBS) for 300 s, respectively. The dissociation process in kinetics buffer solution (0.2‰ Tween‐20 in 1×PBS) was measured for 300s. All *K*_d_ values were calculated with the use of a 1:1 global fit model. Notably, for mP1, the terminal peptides were applied for the affinity evaluation because intact microprotein of as long as 90 aa was difficult to synthesized by chemical method; while other microproteins were directly for the affinity evaluation.

### 4.8 Preparation and Characterization of Gold‐Coated Microextraction Probe

#### 4.8.1 Preparation of Bare Microprobe

Bare microprobes were fabricated according to our previous protocol [38]. A commercial borosilicate capillary glass was first fixed on the pipette puller and then undrawn into two capillaries with a tip diameter of ca. 0.5 µm, namely bare microprobes. The obtained microprobes were carefully collected and stored under cool and dry conditions.

#### 4.8.2 Preparation of Gold‐Coated Microprobe

Before gold coating, amino‐functionalization was necessary. The obtained bare microprobes were first washed with 1 M HCl and 1 M NaOH for 1 h, successively. After washing with pure water and ethanol, the microprobes were immersed into ethanol containing 4% (v/v) of APTES for 3 h and then washed with ethanol 3–5 times. Then, the microprobes were coated with a thin gold layer according to our previous protocol with slight modification [38]. Briefly, the clean probes were immersed in a gold (Au)‐plating solution (12 mm HAuCl_4_, 0.5 M KHCO_3_, and 25 mm glucose) for 4–5 h at 35°C (air bath) until an obvious gold layer appeared on the surface of each probe. Subsequently, the Au‐coated probes were gently washed with water and anhydrous ethanol 3–5 times, separately. The obtained Au‐coated microprobes were carefully collected and stored under cool and dry conditions.

#### 4.8.3 Preparation of Boronic Acid Functionalized Gold‐Coated Microprobe

The Au‐coated microprobes were first immersed in a mixture of APTES anhydrous ethanol (4%, v/v) at room temperature for 6 h, followed by rinsing with ethanol to remove residual reagents. After that, the amino‐modified Au‐coated probes were immersed in a methanol solution containing 1% (w/w) DFFPBA and 1% (w/w) NaCHBH_3_ at room temperature for 24 h. Finally, the probes were gently washed with water and ethanol, dried at 40°C. The obtained boronic acid functionalized Au‐coated microprobes were carefully collected and stored under cool and dry condition.

#### 4.8.4 Preparation of MIP‐Based Au‐Based Microextraction Probe

The obtained boronic acid functionalized Au‐coated microprobes were successively modified molecularly imprinted and cladded polymers according to our previously reported BOSIC method [4], mainly including three steps: 1) template immobilization; 2) boronate‐affinity oriented surface imprinting and cladding; 3) template removal.

1) Template immobilization. Each boronic acid functionalized Au‐coated microprobe was immersed into the 0.1 mL of 50 mm ammonium bicarbonate buffer containing 500 mm sodium chloride (pH 8.5) containing 0.1 mg of glycated epitope peptides, including Fru‐KMGAKAPRGPKVA, Fuc‐KMLHSKLLFSSQI, Fru‐KMYILLWAFPNLI, Fru‐KMGQTHPLLSKPS, Fru‐MSGSYWSC‐QAHT, Fru‐MYVTDPESPAAW, and Fru‐MVTVRAGPLPLP, respectively. The obtained probes were set aside at room temperature for 3 h. The templated epitope‐immobilized microprobes were gently washed with the ammonium bicarbonate buffer for three times, immediately followed by imprinting.
2) BOSIC on microprobe. A batch of the obtained templated epitope‐immobilized microprobes were immersed into 40 mL of anhydrous ethanol containing 0.7 mL of ammonium hydroxide and 10 mL of pure water. Then 10 mL of anhydrous ethanol containing different molar ratios of APTES, UPTES, IBTES, BnTES and TEOS (10 mm, total concentration) was added to the suspension and the mixture was gently stirred at room temperature for 45 min. The imprinted microprobes were gently washed by with ethanol for three times, and re‐immersed into 40 mL of anhydrous ethanol containing 0.7 mL of ammonium hydroxide and 10 mL of pure water. Subsequently, 10 mL of TEOS anhydrous ethanol (10 mm) was added and the mixture was gently stirred at room temperature for 10 min. The resulting epitope‐imprinted and cladded microprobes were gently washed with anhydrous ethanol.
3) Template removal. To remove the template, the epitope‐imprinted microprobes were dispersed into 10 mL of 0.1 M acetic acid containing 50%(v/v) acetonitrile and gently shaken for 12 h at room temperature. The obtained epitope‐imprinted microprobes were washed with pure water for three times and then dried at 40°C under vacuum for 24 h.

### 4.9 Preparation and Characterization of Silver Nanocube‐Based Raman Nanotag

#### 4.9.1 Synthesis of Silver Nanocube (AgNC)

In this work, AgNCs were employed as plasmonic core for preparation of Raman nanotag. AgNCs were synthesized according to a reported one‐pot synthesis method [56]. Briefly, 6.0 mL of EG was added into a glass vial and preheated at 150°C under magnetic stirring for 1 h. Subsequently, 0.07 mL of 3.0 mm Na_2_S solution in EG, 1.5 mL of 20 mg/mL PVP solution in EG, and 0.5 mL of 48 mg/mL AgNO_3_ solution in EG were successively added. The reaction was continued at 150°C for 20 min and then quenched in an ice bath. The obtained AgNCs were first diluted with 20 mL of acetone washed and then washed with ultrapure anhydrous ethanol and pure water for three times. The resulting AgNCs were collected via centrifugation and finally stored in pure water at 4°C.

#### 4.9.2 Synthesis of Agcube@PATP@SiO_2_ Nanostructures

The obtained AgNCs were diluted with 40 mL of anhydrous ethanol under magnetic stirring. Then, 50 µL of 1 mm PATP (dissolved in ethanol) was added and incubate for 60 min. Afterward, 10 mL of pure water and 0.7 mL of ammonium hydroxide was added. Subsequently, 10 mL of TEOS anhydrous ethanol (10 mm) was added and the mixture was gently stirred at room temperature for 50 min. The resulting Agcube@PATP@SiO_2_ NPs were collected by centrifuging and washing with anhydrous ethanol for three times.

#### 4.9.3 Synthesis of Agcube@PATP@SiO_2_@DFFPBA Nanostructures

The obtained Agcube@PATP@SiO_2_ NPs were dispersed into 30 mL of anhydrous ethanol by ultrasonication. Then, 0.1 mL APTES was added drop by drop into the mixture under vigorous stirring. After reaction at room temperature for 1 h, the resulting amino‐functionalized NPs were collected by centrifugation and washed with anhydrous ethanol for three times. The resulting NPs were added into 30 mL anhydrous ethanol containing 1% (w/w) DFFPBA and 1% (w/w) sodium cyanoborohydride, the mixture was vigorously stirred for 24 h at room temperature. Finally, the obtained Agcube@PATP@SiO_2_@DFFPBA NPs were collected by centrifugation and washed with anhydrous ethanol for three times.

#### 4.9.4 Synthesis of AgNC@PATP@SiO_2_@BA@MIP Nanostructures

The obtained Agcube@PATP‐@SiO_2_@DFFPBA NPs were successively modified molecularly imprinted and cladded polymers according to our previously reported BOSIC method [4], mainly including three steps: 1) template immobilization; 2) boronate‐affinity oriented surface imprinting and cladding; 3) template removal.

1) Template immobilization: 20 mg of AgNC@PATP@SiO_2_@BA nanostructures were dispersed into 10 mL of 50 mm ammonium bicarbonate buffer containing 500 mm sodium chloride (pH 8.5) containing 0.1 mg of glycated epitope peptides, including PVPSAQDFWTFMK‐Fru, IFKEFGLKGAKLK‐Fru, ENKSHRKYNMFYK‐Fru, SSQGLLPVLPLQK‐Fru, NVGKRNA‐RAAGK‐Fru, LPSVSPAELWNK‐Fru, and HPRLGAEEPPLK‐Fru. The resulting mixture was kept shaking at room temperature for 3 h. The templated epitope‐immobilized AgNC@PATP@SiO_2_@BA nanostructures were collected by centrifuging and washing with the ammonium bicarbonate buffer.
2) BOSIC on nanotag: The obtained templated epitope‐immobilized nanostructures were re‐dispersed into 40 mL of anhydrous ethanol containing 0.7 mL of ammonium hydroxide and 10 mL of pure water. Then, 10 mL of anhydrous ethanol containing different molar ratios of APTES, UPTES, IBTES, BnTES, and TEOS (10 mm, total concentration) was added to the suspension and the mixture was gently stirred at room temperature for 45 min. The imprinted nanostructures were collected by centrifugation, washed by with ethanol for three times, and re‐dispersed into 40 mL of anhydrous ethanol containing 0.7 mL of ammonium hydroxide and 10 mL of pure water. Subsequently, 10 mL of TEOS anhydrous ethanol (10 mm) was added and the mixture was gently stirred at room temperature for 10 min. The resulting epitope‐imprinted and cladded nanostructures were collected by centrifuging and washing with anhydrous ethanol.
3) Template removal: To remove the template, the epitope‐imprinted and cladded nanostructures were dispersed into 10 mL of 0.1 M acetic acid containing 50%(v/v) acetonitrile and shaken for 12 h at room temperature. The obtained epitope‐imprinted nanostructures were washed with pure water for three times and then stored at 4°C under until use.

### 4.10 Cross‐Reactivity Test

The selectivity of MIP‐based PISA was evaluated using mP2, Cyt C, BSA, RNase A, IgG, Glc, Gal, GlcNAc, AMP, ADP, and ATP as test compounds. First, both mP2 solution of 1 ng/mL and interference protein solutions of 1 µg/mL dissolved in 10 mm phosphate buffer (pH 7.4) were prepared. Subsequently, each microprobe was immersed into 0.1 mL of the corresponding solution and then was kept at room temperature for 10 min. After that, the above microprobe was washed with 1 mL of 10 mm phosphate buffer solution (pH 7.4) for three times. Next, the probe was immersed into 0.1 mL of the corresponding nanotag sol for 10 min. Finally, the microprobe was washed with 1 mL of 10 mm phosphate buffer solution (pH 7.4) for three times. The microprobe was subjected to MIP‐based PISA analysis. The amount of target microprotein on the probe was determined by measuring the Raman intensity at 1438 cm^−1^. The measurement was repeated three times. For the blank control experiment, all steps were the same as above except that no protein was added to the test solution.

### 4.11 Establishment of Standard Curve

Each MIP‐modified microprobe was immersed into 0.1 mL of different concentrations of microprotein solutions, including 0.0001, 0.001, 0.01, 0.1, 1.0, 10.0, 100.0, and 1000.0 ng/mL. After the above solution was kept at room temperature for 10 min, the probes were washed with 1 mL of phosphate buffer (pH 7.4, 10 mm) for three times. Subsequently, the obtained microprobes were immersed into 0.1 mL of the corresponding nanotag sol for 10 min. Finally, the microprobes were washed with 1 mL of 10 mm phosphate buffer solution (pH 7.4) for three times. The concentration of target microprotein in the solution was determined by measuring the Raman intensity at 1438 cm^−1^. The above experiments were repeated three times.

### 4.12 Reproducibility Test

The reproducibility of MIP‐based PISA was evaluated using mP2 as model compound. Ten mP2 N‐terminal epitope‐imprinted microprobes were randomly selected from a batch of probes for test. First, mP2 solution of 1 µg/mL dissolved in 10 mm phosphate buffer (pH 7.4) were prepared. Subsequently, each microprobe was immersed into 0.1 mL of the corresponding solution and then was kept at room temperature for 10 min. After that, the above microprobe was washed with 1 mL of 10 mm phosphate buffer solution (pH 7.4) for three times. Next, the probe was immersed into 0.1 mL of the corresponding nanotag sol for 10 min. Finally, the microprobe was washed with 1 mL of 10 mm phosphate buffer solution (pH 7.4) for three times. The microprobe was subjected to MIP‐based PISA analysis.

### 4.13 Cell Culture

Cell lines (HeLa, Du145, MCF10A, HepG2, PANC1, and HPDE6) were seeded in DMEM medium with 10% fetal bovine serum for 2–3 days (37°C, 5% CO_2_). Cell line SKOV3 was seeded in 5A medium with 10% fetal bovine serum for 2–3 days (37°C, 5% CO_2_). Cell lines (MCF7, L02, and K562) was seeded in 1640 medium with 10% fetal bovine serum for 2–3 days (37°C, 5% CO_2_). MDA‐MB‐231 cell was seeded in 1640 medium with 10% fetal bovine serum for 2–3 days (37°C without CO_2_). For adherent cells, the cell culture medium was removed and the cells remained on the cell culture dishes were washed with culture medium for two times, then the cells were kept in culture medium for subsequent MIP‐based PISA. For suspension cells, the cell culture medium was removed by centrifugation and sedimented cells were then dispersed by blowing. The dispersed cells were suspended in cell culture medium for subsequent MIP‐based PISA.

### 4.14 Single‐Cell MIPs‐Based PISA

All of cell lines were separately seeded in corresponding medium with 10% fetal bovine serum for 1 days and grown on a 35‐mm petri dish until 70%–80% confluency was reached. The petri dish was transferred on the object stage of the micro‐Raman spectrometry. For adhered cells, using the 3D manipulator, the prepared extraction microprobe was directly and precisely inserted into a single living cell. For suspension cells, two 3D manipulators were applied for fixing and inserting into a single living cell, respectively. After 3 min of extraction, the probe was carefully taken out of cell and then gently washed three times with PBS (1 ×). Afterward, the probe was immersed into a labelling nanotag solution, kept at room temperature for 10 min and washed with 1 mL of phosphate buffer (pH 7.4, 10 mm) for three times. Finally, the microprobes were subsequent to MIP‐based PISA.

### 4.15 Single‐Cell Microinjection and MIP‐Based PISA Assay

MCF‐7 cells were seeded in 1640 medium with 10% fetal bovine serum for one day (37°C, 5% CO_2_) and grown on a 35‐mm petri dish until 70%–80% confluency was reached. According to our previous protocol [5], after careful evaluation of microinjection volume, 92 fL of microprotein solution was quantitatively injected into single MCF7 cell. Afterward, microextraction probe was inserted into selected single living cells using a home‐built 3D micromanipulator. After in vivo extraction for 3 min, the probe was drawn out from the living cell and washed with PBS (pH 7.4) for three times. The following procedures were the same to the previous section. The single‐cell spike‐and‐test experiments were also performed as described previously.

### 4.16 Preparation of Cell Lysates

The cultured cells were grown on a 100‐mm plate until 80%–90% confluency was reached. They were detached using trypsin then washed twice with 1 × PBS buffer. Cells were then counted with the cell counter, followed by centrifugation at 800 g for 3 min at 4°C, then the appropriate volume of 1 × PBS buffer was used to resuspend the cells to obtained 5 × 10^6^ cells/mL. A volume of 450 µL of modified RIPA buffer (Triton‐X 100: 1%, NaCl: 150 M and tris‐HCl: 50 mm, pH 7.4) was used to resuspend 20 µL of cells. Then the obtained product was incubated on ice for 30 min, and the debris was pelleted by centrifugation at 3000 rpm for 10 min and the supernatant was collected for further use.

### 4.17 Plasmid Transfection

Twenty‐four hours prior to transfection, cells were seeded into six‐well plates at a density of 1 × 10^6^ cells per well in 2.5 mL complete culture medium. When cell confluence reached approximately 80%, transfection was performed. Briefly, 0.75 µg plasmid DNA was diluted in 150 µL Opti‐MEM and gently mixed. Separately, 15 µL Lipofectamine 2000 was diluted in 150 µL Opti‐MEM and incubated at room temperature for 5 min. The diluted plasmid and Lipofectamine solutions were then combined, gently mixed, and incubated at room temperature for an additional 5 min to allow complex formation. Subsequently, 250 µL of the plasmid–Lipofectamine complexes was added to each well containing 2.25 mL Opti‐MEM, and the plate was gently rocked to ensure even distribution. Cells were incubated at 37°C with 5% CO_2_ for 24 h, after which the transfection medium was replaced with complete culture medium.

### 4.18 Western Blot Analysis

Cells were harvested at approximately 90% confluence, washed twice with ice‐cold PBS, and lysed in RIPA buffer supplemented with PMSF. Lysates were centrifuged at 12 000 × g for 5 min at 4°C, and the supernatants were collected as total protein extracts. Protein concentrations were determined using a BCA assay, after which samples were mixed with 5× loading buffer, boiled for 10 min, and stored at −20°C until analysis.

Equal amounts of protein (60 µg per lane) were separated by SDS–PAGE using 5% stacking gels and 10% resolving gels, followed by transfer onto PVDF membranes. Membranes were briefly stained with Ponceau S to confirm efficient transfer and then blocked in 5% non‐fat milk prepared in TBST at room temperature for 1 h. Membranes were incubated overnight at 4°C with primary antibodies diluted in TBST (see Table S3), washed three times with TBST, and subsequently incubated with HRP‐conjugated secondary antibodies at room temperature for 1 h. Protein signals were visualized using enhanced chemiluminescence and imaged with a Tanon 5200 imaging system. For reprobing, membranes were stripped and reprocessed following standard procedures.

Band intensities were quantified using Image‐Pro Plus 6.0 software. Relative protein expression levels were calculated as the ratio of target protein to internal control protein, or, for phosphorylated proteins, as the ratio of phosphorylated protein to total protein.

### 4.19 MIP‐Based Affinity Purification (AP)‐PISA

The obtained cell lysates with different amounts of cells were diluted with four parts of 1 × PBS buffer in volume. The solutions were boiled for 10 min and cooled to room temperature. Afterward, targeted recognition‐based affinity extraction was performed in three cycles using N‐terminal epitope‐imprinted microbeads (∼5  µm in diameter), N‐epitope‐imprinted sub‐2 microspheres (∼1.5 µm in diameter) and N‐epitope‐imprinted NSs (∼350 nm in diameter) in succession. For the first extraction, 20 mg of epitope‐imprinted microbeads were dispersed into the above solution through vigorous vortex and the mixture was shaken for 2 h on a rotator. After complete precipitation by centrifugation, the supernatant was collected for second extraction. The obtained solution was diluted to 1 mL with 1 × PBS, and 20 mg of epitope‐imprinted sub‐2 microspheres were dispersed into the above solution through vigorous vortex and the mixture was shaken for 2 h on a rotator. After complete precipitation by centrifugation, the supernatant was collected for third extraction. The obtained solution was diluted to 1 mL with 1 × PBS, and 20 mg of epitope‐imprinted NSs were dispersed into the above solution through vigorous vortex and the mixture was shaken for 2 h on a rotator. After complete precipitation by centrifugation, the supernatant was taken out and the collected nanospheres were washed with 0.1 mL of 1 × PBS for three time. Finally, the sediment was dispersed into 50 µL of 0.1 M acetic acid containing 50% (v/v) acetonitrile and shaken for 6 h at room temperature. The nanospheres were completely separated by centrifugation and the eluant was going to be merged with other eluants as follows.

The precipitate obtained from first extraction was carefully redispersed with 1 mL of 1 × PBS by gentle shaking for 2 h. After complete precipitation by centrifugation, the supernatant was collected for second and third extraction as mentioned above. The precipitate after second centrifugation was carefully redispersed with 1 mL of 1 × PBS by gentle shaking for 2 h to perform third extraction. The procedure was the same to the second extraction except using epitope‐imprinted NSs as affinity‐purification materials. At last, all of eluants were collected and concentration by lyophilization and diluted with 100 µL of 1 × PBS for MIP‐based PISA analysis.

### 4.20 MIP‐Based Affinity Purification‐Mass Spectrometry (AP‐MS)

The similar procedures were performed for extraction of target microproteins except in the last step using 100 µL of 0.1 M acetic acid containing 50% (v/v) acetonitrile for ESI‐MS/MS analysis. The MS/MS was operated in data‐dependent acquisition mode. Up to the top 10 most abundant ions with charge 2+ or 3+ from the prior scan were selected with an isolation window of 1.6 Thomson and fragmented by higher‐energy collision dissociation (HCD) with normalized collision energies of 35. The maximum ion injection times for the survey scan and the MS/MS scans were 30 and 60 ms, respectively. The ion target values for the survey scan and the MS/MS scans were set to 1 ×10^5^ and 1 ×10^4^, respectively.

### 4.21 Clinical Tissue Slice Imaging

The clinical human tissue sections in the following tests were provided by Nanjing Drum Tower Hospital, which was approved and enrolled in clinical trial (2025‐0296‐01). All procedures involving human participants were conducted in accordance with the ethical standards of the institutional research committee and the Declaration of Nanjing Drum Tower Hospital. Written informed consent was obtained from all patients prior to sample collection. The tissue specimens were collected during operation and subsequently fixed in formalin and embedded in paraffin until testing. Before test, all sections were covered with paraffin wax according to a standardized workflow. H&E staining was performed according to a standardized workflow. For Raman imaging, the adjacent tissue slice was collected and covered with paraffin. Before test, the paraffin on slice was first eluted by means of gradient elution, then incubated with 200 µL of 500 µg/mL PEGylated terminal imprinted AgNC‐based nanotags. After incubation of 30 min, the excessive nanotags were washed out with 1 × PBS. The resulting tissue slides were profiled under the confocal Raman spectrometry. The PEGylated nanotags were prepared as the same procedure mentioned above except in cladding step that the cladding duration was reduced into 5 min and the amino group was modified with APTES for 5 min followed by functionalization with abundant NHS‐PEG1000. The obtained PEGylated nanotags were rinsed with 1 × PBS and collected by centrifugation.

### 4.22 Statistical Analysis

Statistical analysis was performed in OriginLab Pro (Ver. 2025b). The experimental data were presented as mean ± SEM. Unpaired two‐sided Student's *t* test or one‐way analysis of variance (ANOVA) was used to evaluate the significance of mean differences. Significance was indicated by * for *p* < 0.05, ** for *p* < 0.01, and *** for *p* < 0.001.

## Author Contributions

H.H. and A.Z. contributed equally to this work. Z.L. conceived the project and designed the research. H.H. conducted most of the experimental work, computational simulation, and data analysis. A.Z. performed the bioinformatic analysis. Z.G. performed material characterizations. S.G. predicted the microprotein structures. Y.L. conducted the affinity investigation. J.C. predicted the RNA structures. S.S. performed partial tissue imaging tests. W.G., Q.L., and J.H. confirmed tissue annotation. H.H. and A.Z. drafted the manuscript and Z.L. finalized the manuscript. All authors approved the final version of the manuscript.

## Funding

The Key Scientific Instrumentation Grant (22327804 and 21627810) from the National Natural Science Foundation of China, and the Young Scientists Grant (22004064) from the National Natural Science Foundation of China.

## Conflicts of Interest

The authors declare no conflicts of interest.

## Supporting information




**Supporting File**: advs74710‐sup‐0001‐SuppMat.docx.

## Data Availability

The data that support the findings of this study are available from the corresponding author upon reasonable request.

## References

[1] J. P. Couso and P. Patraquim , “Classification and Function of Small Open Reading Frames,” Nature Reviews Molecular Cell Biology 18, no. 9 (2017): 575–589, 10.1038/nrm.2017.58. 28698598

[2] S. J. Andrews and J. A. Rothnagel , “Emerging Evidence for Functional Peptides Encoded by Short Open Reading Frames,” Nature Reviews Genetics 15, no. 3 (2014): 193–204, 10.1038/nrg3520. 24514441

[3] J. M. Mudge , J. Ruiz‐Orera , J. R. Prensner , et al., “Standardized Annotation of Translated Open Reading Frames,” Nature Biotechnology 40, no. 7 (2022): 994–999, 10.1038/s41587-022-01369-0. PMC9757701 35831657

[4] E. Vieira de Souza , A. L. Bookout , C. A. Barnes , et al., “Rp3: Ribosome Profiling‐Assisted Proteogenomics Improves Coverage and Confidence During Microprotein Discovery,” Nature Communications 15, no. 1 (2024): 6839, 10.1038/s41467-024-50301-4. PMC11316118 39122697

[5] J. R. Prensner , O. M. Enache , V. Luria , et al., “Noncanonical Open Reading Frames Encode Functional Proteins Essential for Cancer Cell Survival,” Nature Biotechnology 39, no. 6 (2021): 697–704, 10.1038/s41587-020-00806-2. PMC8195866 33510483

[6] N. G. D'Lima , J. Ma , L. Winkler , et al., “A Human Microprotein that Interacts With the mRNA Decapping Complex,” Nature Chemical Biology 13, no. 2 (2017): 174–180, 10.1038/nchembio.2249. 27918561 PMC5247292

[7] B. W. Wright , Z. Yi , J. S. Weissman , and J. Chen , “The Dark Proteome: Translation From Noncanonical Open Reading Frames,” Trends in Cell Biology 32, no. 3 (2022): 243–258, 10.1016/j.tcb.2021.10.010. 34844857 PMC8934435

[8] C. He , C. Jia , Y. Zhang , and P. Xu , “Enrichment‐Based Proteogenomics Identifies Microproteins, Missing Proteins, and Novel smORFs in *Saccharomyces cerevisiae* ,” Journal of Proteome Research 17, no. 7 (2018): 2335–2344, 10.1021/acs.jproteome.8b00032. 29897761

[9] M. Li , X. Li , Y. Zhang , et al., “Micropeptide MIAC Inhibits HNSCC Progression by Interacting With Aquaporin 2,” Journal of the American Chemical Society 142, no. 14 (2020): 6708–6716, 10.1021/jacs.0c00706. 32176498

[10] C. Zheng , Y. Wei , P. Zhang , et al., “CRISPR–Cas9‐Based Functional Interrogation of Unconventional Translatome Reveals Human Cancer Dependency on Cryptic Non‐Canonical Open Reading Frames,” Nature Structural & Molecular Biology 30, no. 12 (2023): 1878–1892, 10.1038/s41594-023-01117-1. PMC10716047 37932451

[11] D. A. Hofman , J. R. Prensner , and S. van Heesch , “Microproteins in Cancer: Identification, Biological Functions, and Clinical Implications,” Trends in Genetics 41, no. 2 (2024): 146–161, 10.1016/j.tig.2024.09.002. 39379206 PMC11794034

[12] C. A. Makarewich and E. N. Olson , “Mining for Micropeptides,” Trends in Cell Biology 27, no. 9 (2017): 685–696, 10.1016/j.tcb.2017.04.006. 28528987 PMC5565689

[13] M. P. Nagle , G. S. Tam , E. Maltz , Z. Hemminger , and R. Wollman , “Bridging Scales: From Cell Biology to Physiology Using In Situ Single‐Cell Technologies,” Cell Systems 12, no. 5 (2021): 388–400, 10.1016/j.cels.2021.03.002. 34015260

[14] J. Chen , L. Larsson , A. Swarbrick , and J. Lundeberg , “Spatial Landscapes of Cancers: Insights and Opportunities,” Nature Reviews Clinical Oncology 21, no. 9 (2024): 660–674, 10.1038/s41571-024-00926-7. 39043872

[15] J. A. Christopher , C. Stadler , C. E. Martin , et al., “Subcellular Proteomics,” Nature Reviews Methods Primers 1, no. 1 (2021): 32, 10.1038/s43586-021-00029-y. PMC8451152 34549195

[16] I. Amelio and G. Melino , “Context is Everything: Extrinsic Signalling and Gain‐of‐Function p53 Mutants,” Cell Death Discovery 6, no. 1 (2020): 16, 10.1038/s41420-020-0251-x. 32218993 PMC7090043

[17] M. Labib and S. O. Kelley , “Single‐Cell Analysis Targeting the Proteome,” Nature Reviews Chemistry 4, no. 3 (2020): 143–158, 10.1038/s41570-020-0162-7. 37128021

[18] T. Stuart and R. Satija , “Integrative Single‐Cell Analysis,” Nature Reviews Genetics 20, no. 5 (2019): 257–272, 10.1038/s41576-019-0093-7. 30696980

[19] G. Vlatakis , L. I. Andersson , R. Müller , and K. Mosbach , “Drug Assay Using Antibody Mimics Made by Molecular Imprinting,” Nature 361, no. 6413 (1993): 645–647, 10.1038/361645a0. 8437624

[20] K. Haupt , P. X. Medina Rangel , and B. T. S. Bui , “Molecularly Imprinted Polymers: Antibody Mimics for Bioimaging and Therapy,” Chemical Reviews 120, no. 17 (2020): 9554–9582, 10.1021/acs.chemrev.0c00428. 32786424

[21] M. F. Cardinal , E. Vander Ende , R. A. Hackler , et al., “Expanding Applications of SERS Through Versatile Nanomaterials Engineering,” Chemical Society Reviews 46, no. 13 (2017): 3886–3903, 10.1039/c7cs00207f. 28640313

[22] S. P. B. Teixeira , R. L. Reis , N. A. Peppas , M. E. Gomes , and R. M. A. Domingues , “Epitope‐Imprinted Polymers: Design Principles of Synthetic Binding Partners for Natural Biomacromolecules,” Science Advances 7, no. 44 (2021): 9884, 10.1126/sciadv.abi9884. PMC8555893 34714673

[23] Y. L. Diao , J. Gao , Y. Ma , and G. Q. Pan , “Epitope‐Imprinted Biomaterials With Tailor‐Made Molecular Targeting for Biomedical Applications,” Bioactive Materials 45 (2025): 162–180, 10.1016/j.bioactmat.2024.11.012. 39634057 PMC11616479

[24] R. R. Xing , Z. C. Guo , H. F. Lu , Q. Zhang , and Z. Liu , “Molecular Imprinting and Cladding Produces Antibody Mimics with Significantly Improved Affinity and Specificity,” Science Bulletin 67, no. 3 (2022): 278–287, 10.1016/j.scib.2021.10.006. 36546077

[25] S. Azam , F. Yang , and X. Wu , “Finding Functional Microproteins,” Trends in Genetics 41, no. 2 (2025): 107–118, 10.1016/j.tig.2024.12.001. 39753408 PMC11794006

[26] S. van Heesch , F. Witte , V. Schneider‐Lunitz , et al., “The Translational Landscape of the Human Heart,” Cell 178, no. 1 (2019): 242–260, 10.1016/j.cell.2019.05.010. 31155234

[27] Z. Ji , R. Song , A. Regev , and K. Struhl , “Many lncRNAs, 5'UTRs, and Pseudogenes are Translated and Some are Likely to Express Functional Proteins,” Elife 4 (2015): 08890, 10.7554/eLife.08890. PMC4739776 26687005

[28] J. Chen , A. D. Brunner , J. Z. Cogan , et al., “Pervasive Functional Translation of Noncanonical Human Open Reading Frames,” Science 367, no. 6482 (2020): 1140–1146, 10.1126/science.aay0262. 32139545 PMC7289059

[29] T. F. Martinez , Q. Chu , C. Donaldson , D. Tan , M. N. Shokhirev , and A. Saghatelian , “Accurate Annotation of Human Protein‐Coding Small Open Reading Frames,” Nature Chemical Biology 16, no. 4 (2020): 458–468, 10.1038/s41589-019-0425-0. 31819274 PMC7085969

[30] B. Gaertner , S. van Heesch , V. Schneider‐Lunitz , et al., “A Human ESC‐Based Screen Identifies a Role for the Translated lncRNA LINC00261 in Pancreatic Endocrine Differentiation,” Elife 9 (2020): 58659, 10.7554/eLife.58659. PMC7423336 32744504

[31] A. Raj , S. H. Wang , H. Shim , et al., “Thousands of Novel Translated Open Reading Frames in Humans Inferred by Ribosome Footprint Profiling,” Elife 5 (2016): 13328, 10.7554/eLife.13328. PMC4940163 27232982

[32] A. Matsumoto , A. Pasut , M. Matsumoto , et al., “mTORC1 and Muscle Regeneration are Regulated by the LINC00961‐Encoded SPAR Polypeptide,” Nature 541, no. 7636 (2017): 228–232, 10.1038/nature21034. 28024296

[33] Y. Huang , H. Lu , Y. Liu , et al., “Micropeptide hSPAR Regulates Glutamine Levels and Suppresses Mammary Tumor Growth via a TRIM21‐P27KIP1‐mTOR Axis,” The EMBO Journal 44, no. 5 (2025): 1414–1441, 10.1038/s44318-024-00359-z. 39875724 PMC11876615

[34] K. Li , J. Kong , S. Zhang , T. Zhao , and W. Qian , “Distance‐Dependent Inhibition of Translation Initiation by Downstream Out‐of‐Frame AUGs is Consistent With a Brownian Ratchet Process of Ribosome Scanning,” Genome Biology 23, no. 1 (2022): 254, 10.1186/s13059-022-02829-1. 36510274 PMC9743702

[35] K. Haupt and K. Mosbach , “Molecularly Imprinted Polymers and Their Use in Biomimetic Sensors,” Chemical Reviews 100, no. 7 (2000): 2495–2504, 10.1021/cr990099w. 11749293

[36] J. Jumper , R. Evans , A. Pritzel , et al., “Highly Accurate Protein Structure Prediction With AlphaFold,” Nature 596, no. 7873 (2021): 583–589, 10.1038/s41586-021-03819-2. 34265844 PMC8371605

[37] J. Liu , D. Yin , S. Wang , H. Y. Chen , and Z. Liu , “Probing Low‐Copy‐Number Proteins in a Single Living Cell,” Angewandte Chemie International Edition 55, no. 42 (2016): 13215–13218, 10.1002/anie.201608237. 27634436

[38] J. Liu , H. He , D. Xie , Y. Wen , and Z. Liu , “Probing Low‐Copy‐Number Proteins in Single Living Cells Using Single‐Cell Plasmonic Immunosandwich Assays,” Nature Protocols 16, no. 7 (2021): 3522–3546, 10.1038/s41596-021-00547-9. 34089021

[39] G. Ge , Y. Wen , P. Li , Z. Guo , and Z. Liu , “Single‐Cell Plasmonic Immunosandwich Assay Reveals the Modulation of Nucleocytoplasmic Localization Fluctuation of ABL1 on Cell Migration,” Analytical Chemistry 95, no. 48 (2023): 17502–17512, 10.1021/acs.analchem.3c02593. 38050674

[40] Y. Wen , G. Ge , D. Xie , and Z. Liu , “Single Living Cell Analysis Decodes Dynamical Signaling Patterns Triggering Different Phenotypes of Cell Migration,” Analytical Chemistry 95, no. 14 (2023): 6080–6089, 10.1021/acs.analchem.3c00233. 36995353

[41] A. Zhang , Z. Guo , G. Ge , and Z. Liu , “Insights Into In Vivo Environmental Effects on Quantitative Biochemistry in Single Cells,” Analytical Chemistry 95, no. 47 (2023): 17246–17255, 10.1021/acs.analchem.3c03102. 37963214

[42] A. Zhang , W. Qu , P. Guan , Y. Li , and Z. Liu , “Single Living Cell “Observation‐Analysis” Integrated Platform Decodes Cell Migration Plasticity Orchestrated by Nucleocytoplasmic STAT3,” Nano Letters 24, no. 27 (2024): 8361–8368, 10.1021/acs.nanolett.4c01841. 38940365

[43] J. Liu , Y. Wen , H. He , H. Y. Chen , and Z. Liu , “Probing Cytoplasmic and Nuclear microRNAs in Single Living Cells via Plasmonic Affinity Sandwich Assay,” Chemical Science 9, no. 36 (2018): 7241–7246, 10.1039/c8sc02533a. 30288244 PMC6148463

[44] J. Liu , D. Xie , and Z. Liu , “Probing Nucleus‐Enriched Proteins in Single Living Cells via a Subcellular‐Resolved Plasmonic Immunosandwich Assay,” The Analyst 146, no. 9 (2021): 2878–2885, 10.1039/d1an00003a. 33687045

[45] L. Zhang , P. Zhang , G. Wang , et al., “Ras and Rap Signal Bidirectional Synaptic Plasticity via Distinct Subcellular Microdomains,” Neuron 98, no. 4 (2018): 783–800, 10.1016/j.neuron.2018.03.049. 29706584 PMC6192044

[46] O. Braubach , S. Basak , M. Gallina , et al., “P01.06 Spatially‐Resolved, Highly Multiplexed Biomarker Analysis of Cancerous and Normal Human Breast Tissues,” Journal of ImmunoTherapy of Cancer 8, no. 2 (2020): A10, 10.1136/jitc-2020-ITOC7.19.

[47] Y. J. Li , S. H. Chien , R. Huang , et al., “A Platform to Deliver Single and Bi‐Specific Cas9/Guide RNA to Perturb Genes In Vitro and In Vivo,” Molecular Therapy 32, no. 10 (2024): 3629–3649, 10.1016/j.ymthe.2024.07.025. 39091030 PMC11489542

[48] M. Ozturk , A. Freiwald , J. Cartano , et al., “Proteome Effects of Genome‐Wide Single Gene Perturbations,” Nature Communications 13, no. 1 (2022): 6153, 10.1038/s41467-022-33814-8. PMC9579165 36257942

[49] J. M. Sheltzer and A. Amon , “The Aneuploidy Paradox: Costs and Benefits of an Incorrect Karyotype,” Trends in Genetics 27 (2011): 446–453, 10.1016/j.tig.2011.07.003. 21872963 PMC3197822

[50] L. Poliseno , M. Lanza , and P. P. Pandolfi , “Coding, or Non‐Coding, that is the Question,” Cell Research 34, no. 9 (2024): 609–629, 10.1038/s41422-024-00975-8. 39054345 PMC11369213

[51] T. Guo , J. A. Steen , and M. Mann , “Mass‐Spectrometry‐Based Proteomics: From Single Cells to Clinical Applications,” Nature 638, no. 8052 (2025): 901–911, 10.1038/s41586-025-08584-0. 40011722

[52] K. Motone , D. Kontogiorgos‐Heintz , J. Wee , et al., “Multi‐Pass, Single‐Molecule Nanopore Reading of Long Protein Strands,” Nature 633, no. 8030 (2024): 662–669, 10.1038/s41586-024-07935-7. 39261738 PMC11410661

[53] M. Steinegger and J. Soding , “MMseqs2 Enables Sensitive Protein Sequence Searching for the Analysis of Massive Data Sets,” Nature Biotechnology 35, no. 11 (2017): 1026–1028, 10.1038/nbt.3988. 29035372

[54] M. Xing , B. Chen , J. Feng , et al., “Confined Growth of Quantum Dots in Silica Spheres by Ion Exchange of “Trapped NH_4_ ^+^” for White‐Light Emission,” Chemistry 5, no. 8 (2019): 2195–2214, 10.1016/j.chempr.2019.06.010.

[55] R. R. Xing , Z. C. Guo , H. F. Lu , Q. Zhang , and Z. Liu , “Molecular Imprinting and Cladding Produces Antibody Mimics With Significantly Improved Affinity and Specificity,” Science Bulletin 67, no. 3 (2022): 278–287, 10.1016/j.scib.2021.10.006. 36546077

[56] S. E. Skrabalak , L. Au , X. Li , and Y. Xia , “Facile Synthesis of Ag Nanocubes and Au Nanocages,” Nature Protocols 2, no. 9 (2007): 2182–2190, 10.1038/nprot.2007.326. 17853874

