# Supplementary material for: An Artificial Antibody‐Based Toolbox Accelerates Validation of Hidden Microproteins Encoded by the Dark Genome
Source: Adv Sci (Weinh). 2026 Mar 3;13(28):e15707. doi: 10.1002/advs.202515707 (PMC13185872; doi:10.1002/advs.202515707)
Supplement: Supplementary file 1 — Supporting File: advs74710‐sup‐0001‐SuppMat.docx. [file ADVS-13-e15707-s001.docx]

Supporting Information

An artificial antibody-based toolbox accelerates validation of hidden microproteins encoded by the dark genome

Hui He, Anqi Zhang, Zhanchen Guo, Qi Li, Song Gao, Wenyan Guan, Ying Li, Jingran Chen, Sijia Sun, Jian He, and Zhen Liu*

Table of content

[**Supplementary Tables** 2](#_Toc223030720)

[**Supplementary Figures** 5](#_Toc223030721)

[**References** 38](#_Toc223030722)

**Supplementary Tables**

**Table S1 Basic biological information of selected microproteins.**

| Name | Gene  Symbol | ORF source | ORF Location (hg 38) | Strand | Sequence | Length (aa) |
| --- | --- | --- | --- | --- | --- | --- |
| mP1 | LINC00961 | LINC00961-ORF7 | chr9:35910420-35910692 | + | MGAKAPRGPKVAQWAMETAVIGVVVVLFVVTVAITCVLCCFSCDSRAQDPQGGPGRSFTVATFRQEASLFTGPVRHAQPVPSAQDFWTFM | 90 |
| mP2 | LINC00961 | LINC00961-ORF5 | chr9:35910722-35910808 | + | MGQTHPLLSKPSLAFPSSQGLLPVLPLQ | 28 |
| mP3 | LINC00961 | LINC00961-ORF6 | chr9:35911244-35911345 | + | MYILLWAFPNLIRKTTKGNRRENKSHRKYNMFY | 33 |
| mP4 | LINC00961 | LINC00961-ORF3 | chr9:35911510-35911650 | + | MLHSKLLFSSQIRGYSVFINRKPILPWPWNKCHAIFKEFGLKGAKL | 46 |
| mP5 | EBLN3P | c9riboseqorf26  (ORF4) | chr9:37080034-37080114 | + | MYVTDPESPAAWDPCLPSVSPAELWN | 26 |
| mP6 | ZDHHC4 | c7riboseqorf13  (uORF4) | chr7:6618222-6618329 | + | MSGSYWSCQAHTAAQEELLFELSVNVGKRNARAAG | 35 |
| mP7 | RNF145 | c5riboseqorf114  (uORF7) | chr5:158636362-158636457 | - | MVTVRAGPLPLPLPPPPQKQHPRLGAEEPPL | 31 |

**Table S2 Comparison of methodologies for microprotein validation.**

| No. | Recognition methods  /customizability | Detection sensitivity | Multiscale validation | Speed | Throughout | Ref |
| --- | --- | --- | --- | --- | --- | --- |
| 1 | Antibody-based method (Western blot)  /medium | ng/mL level | - Cell lysate - Tissue | Medium | Medium | ^[S1-S3]^ |
| 2 | Antibody-based method(Immunofluorescence)  /medium | ng/mL level | - Intact cell - Tissue | Medium | Medium | ^[S2-S4]^ |
| 3 | Flag-fusion-based method method(CRISPR knock-in)  /high | / | - Intact cell - Cell lysate - Tissue | Medium | Medium | ^[S5-S7]^ |
| 4 | Flag-fusion-based method (Epitope tagging)  /high | / | - Intact cell - Cell lysate - Tissue | Medium | Medium | ^[S8-S10]^ |
| 5 | Affinity-purification method  (IP/MS)  /high | ng/mL level | - Cell lysate - Tissue | Slow | Medium | ^[S3,S7,S11-S13]^ |
| 6 | MIP  /very high | pg/mL  level | - Single living cell - Cell lysate - Tissue | Fast | High | This work |

**Table S3** **Guidelines for implementing CLAIMID for novel microprotein validation.**

| No. | Item | Experimental | Index |
| --- | --- | --- | --- |
| 1 | **Candidate Selection and epitope Design.** | Selecting sORFs from omics data / computational biology and choosing optimal linear epitopes | Detected by Ribo-seq or other omics but not detected at protein-level. |
| 2 | **MIP Synthesis and Characterization** | Optimizing the formula of monomers and polymerization conditions | Evaluation of Imprinting factor, cross-reactivity and affinity. |
| 3 | **Specificity validation** | Constructing the overexpression/knockdown cell models and confirm specificity before probing endogenous expression. | A clear signal-to-background ratio |
| 4 | **Expression level determination** | Detecting the target microprotein at both single-cell and cell population level. | Both spectroscopic and MS identification are necessary |
| 5 | **Tissue or *in vivo* validation** | Profiling the target microprotein expressed in clinical tissues or other real biological samples. | A comparison of conventional staining and the proposed imaging is necessary. |

**Supplementary Figures**

**
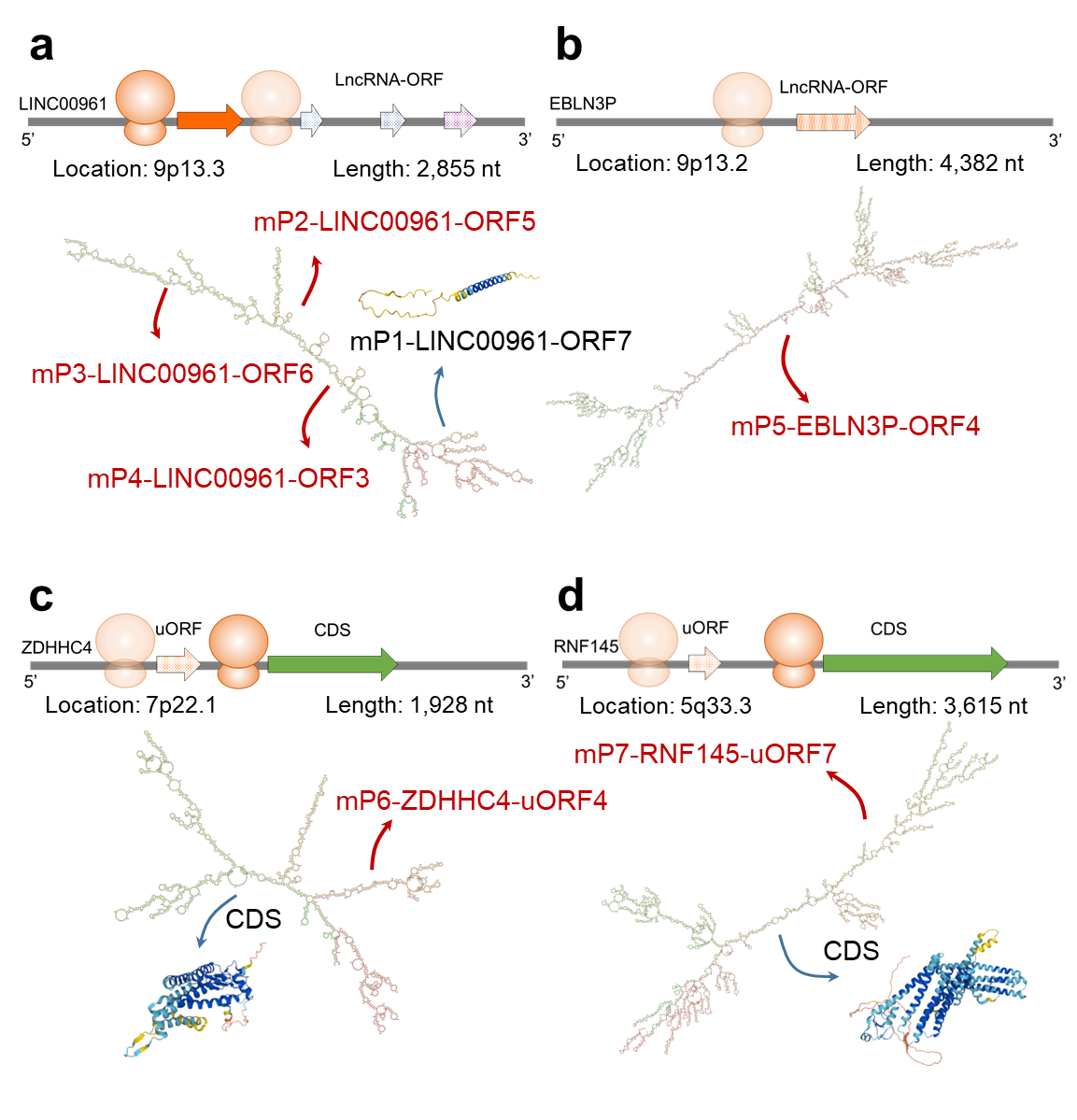
**

**Figure S1. Selection of sORFs from LINC00961 (a), EBLN3P(b), ZDHHC4(c) and RNF145(d).**

**
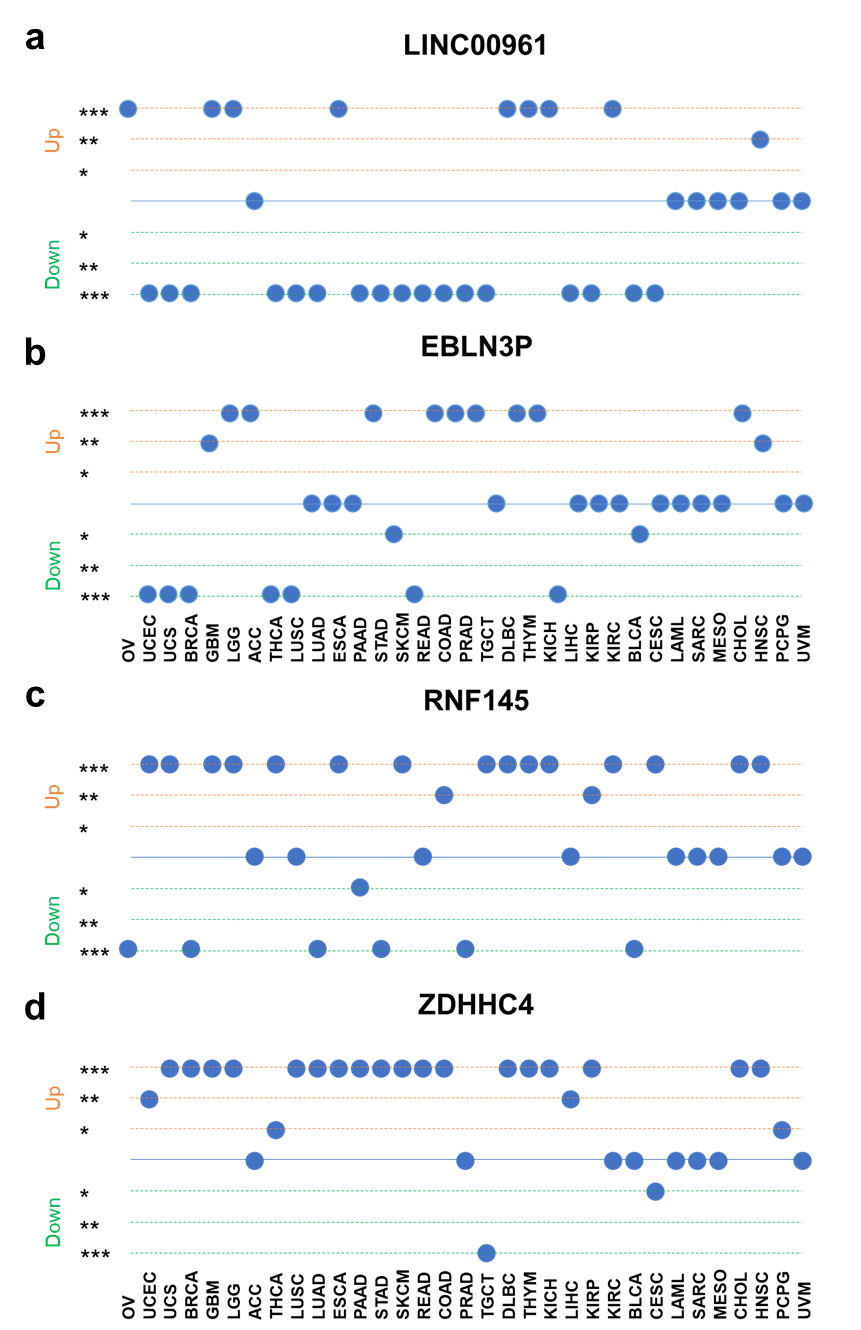
**

**Figure S2. Pan-cancer analysis of four genes including LINC00961 (a), EBLN3P (b), RNF145 (c) and ZDHHC4 (d).** Data were collected from TCGA and GTEx database.

**
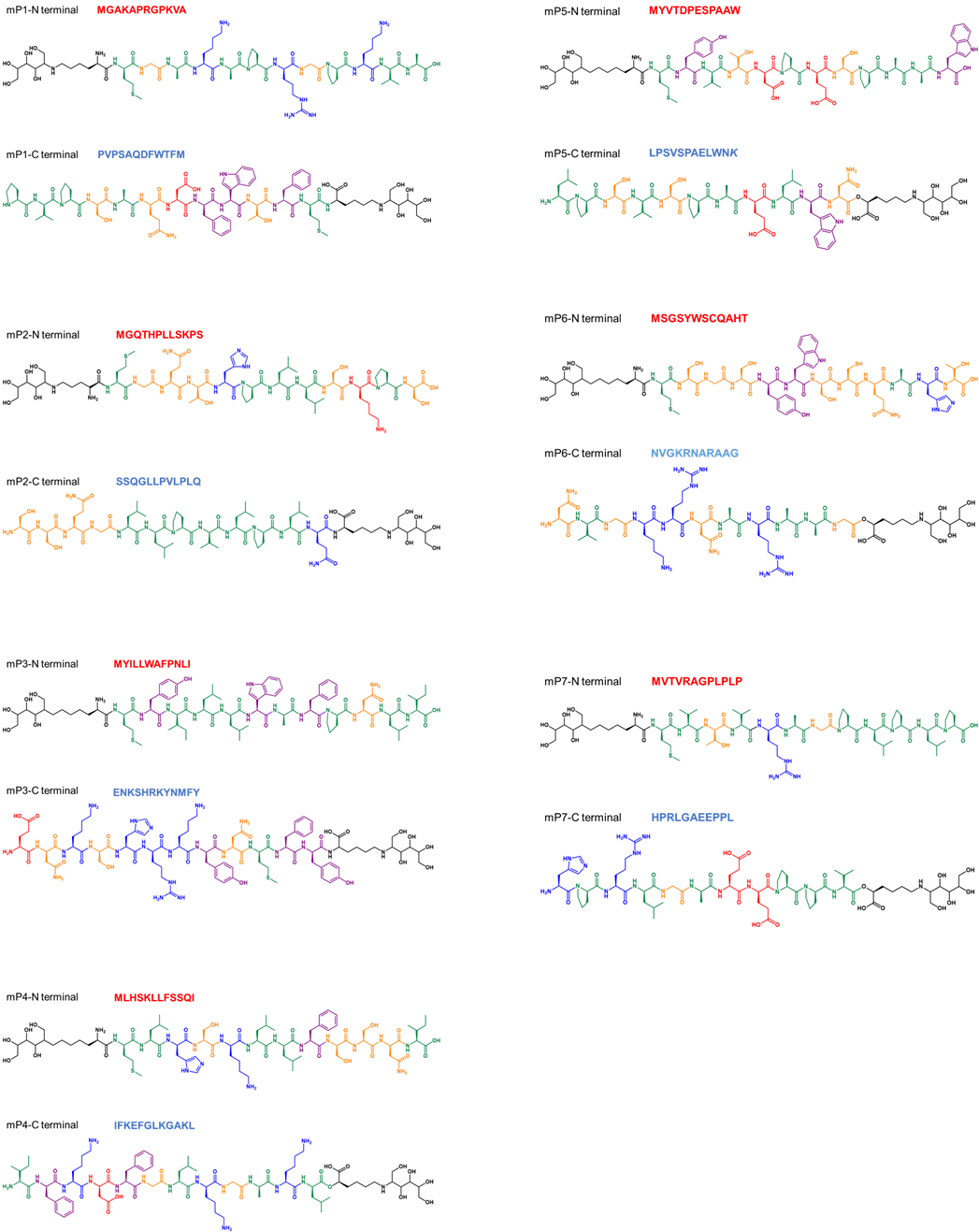
**

**Figure S3. Glycated terminal epitopes (12 aa) of seven selected microproteins.**

**
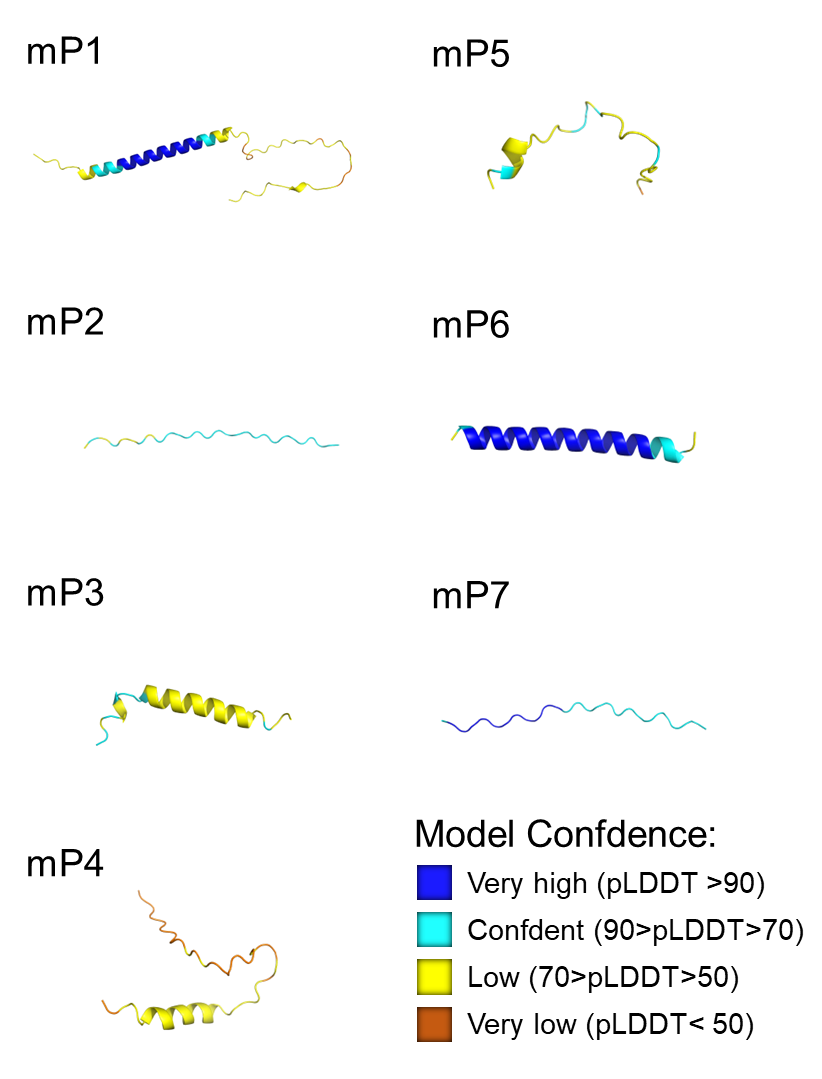
**

**Figure S4. Structure prediction of seven selected microproteins via AlphaFold2.**

**
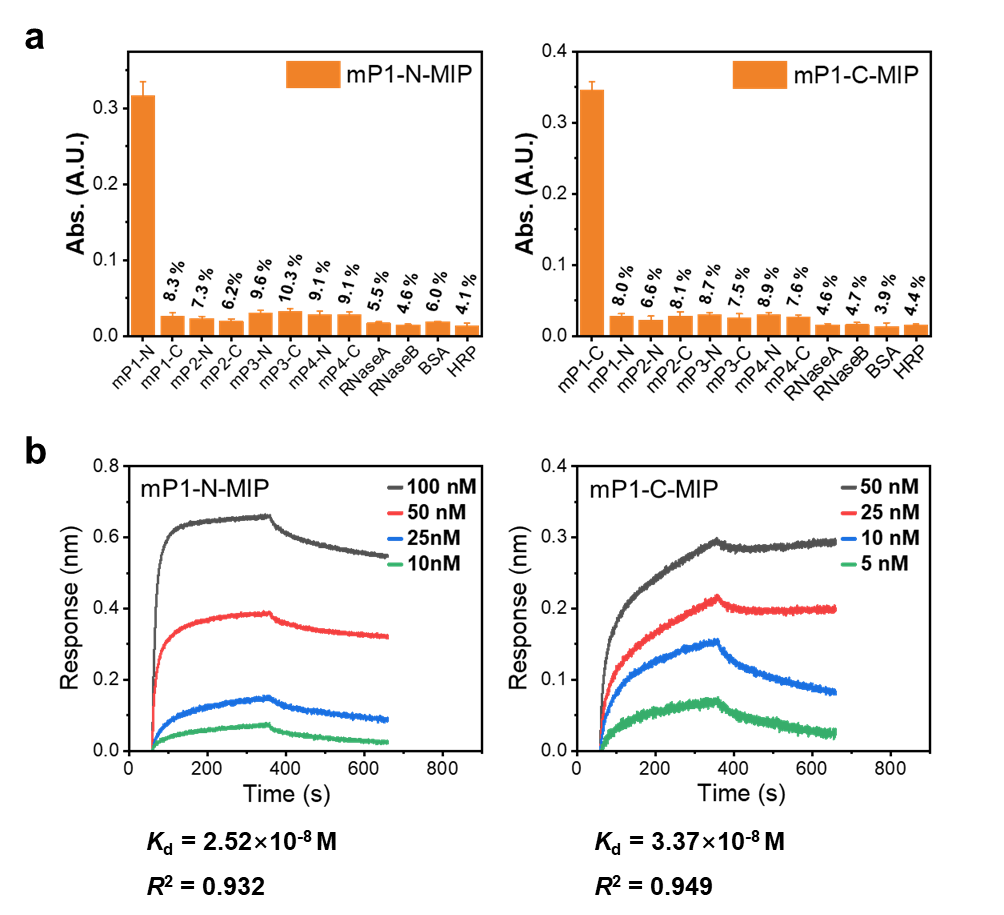
**

**Figure S5. Terminal epitope imprinting of mP1.** a. Selectivity of optimized mP1-N-MIP and mP1-C-MIP. b. BLI adsorption kinetics and dissociation constants of optimized mP1-N-MIP and mP1-C-MIP. Data represent mean ± SEM.  All experiments were performed in triplicate.

**
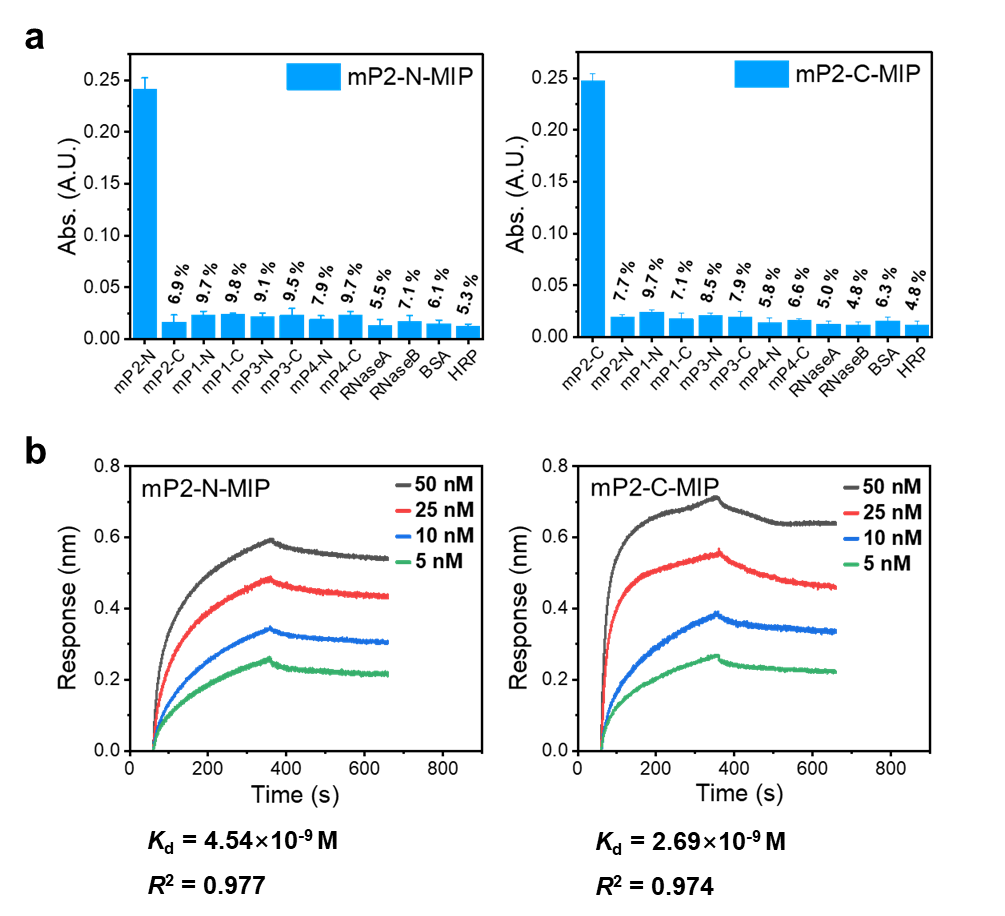
**

**Figure S6. Terminal epitope imprinting of mP2.** a. Selectivity of optimized mP2-N-MIP and mP2-C-MIP. b. BLI adsorption kinetics and dissociation constants of optimized mP2-N-MIP and mP2-C-MIP. Data represent mean ± SEM.  All experiments were performed in triplicate.

**
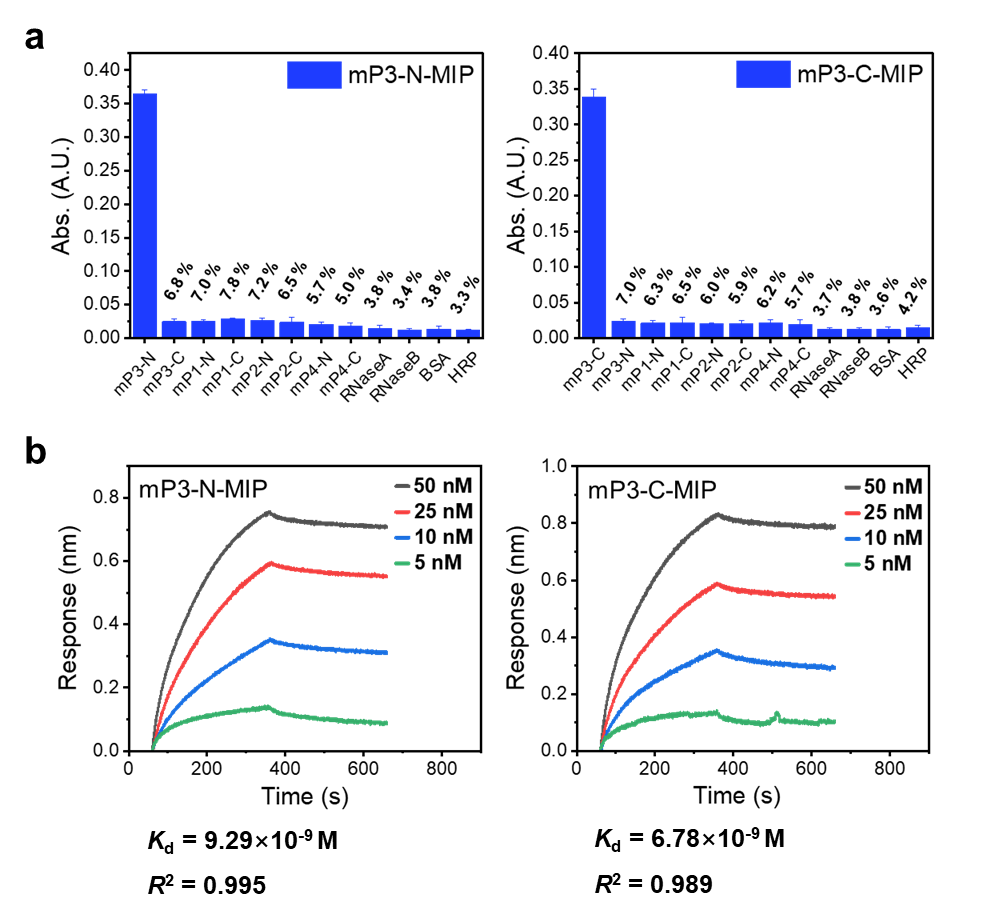
**

**Figure S7. Terminal epitope imprinting of mP3.** a. Selectivity of optimized mP3-N-MIP and mP3-C-MIP. b. BLI adsorption kinetics and dissociation constants of optimized mP3-N-MIP and mP3-C-MIP. Data represent mean ± SEM.  All experiments were performed in triplicate.

**
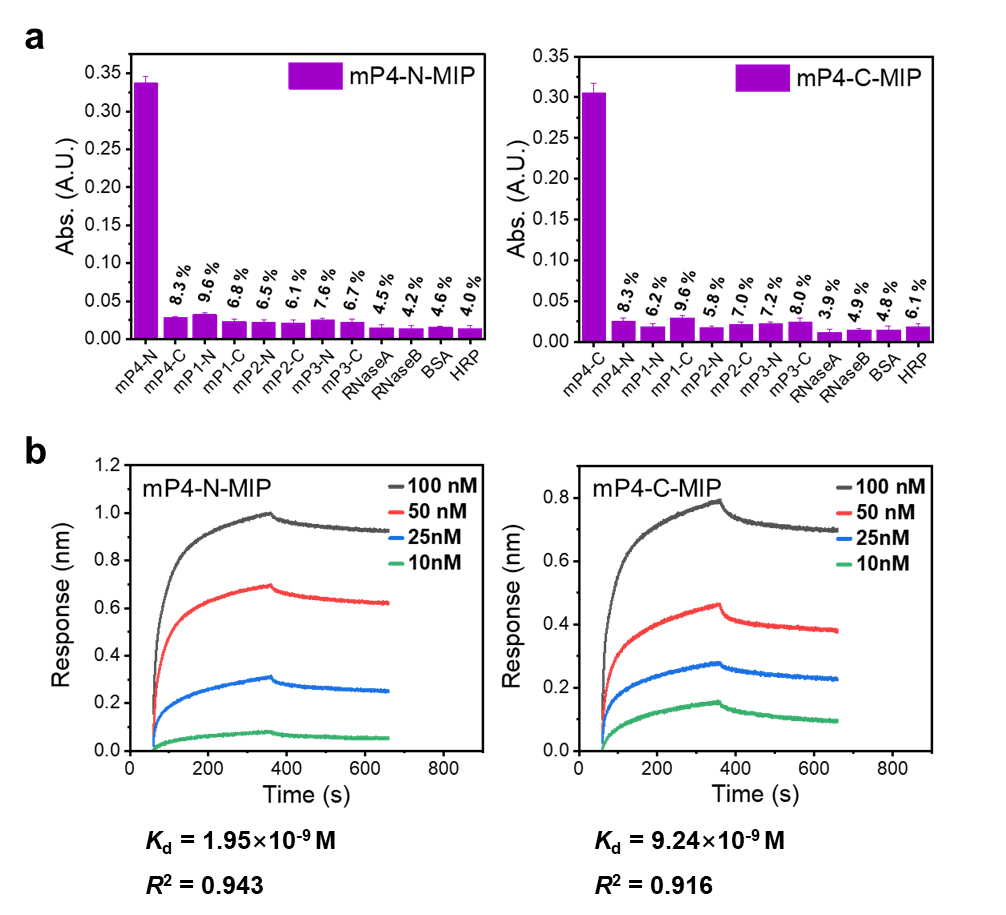
**

**Figure S8. Terminal epitope imprinting of mP4.** a. Selectivity of optimized mP4-N-MIP and mP4-C-MIP. b. BLI adsorption kinetics and dissociation constants of optimized mP4-N-MIP and mP4-C-MIP. Data represent mean ± SEM.  All experiments were performed in triplicate.

**
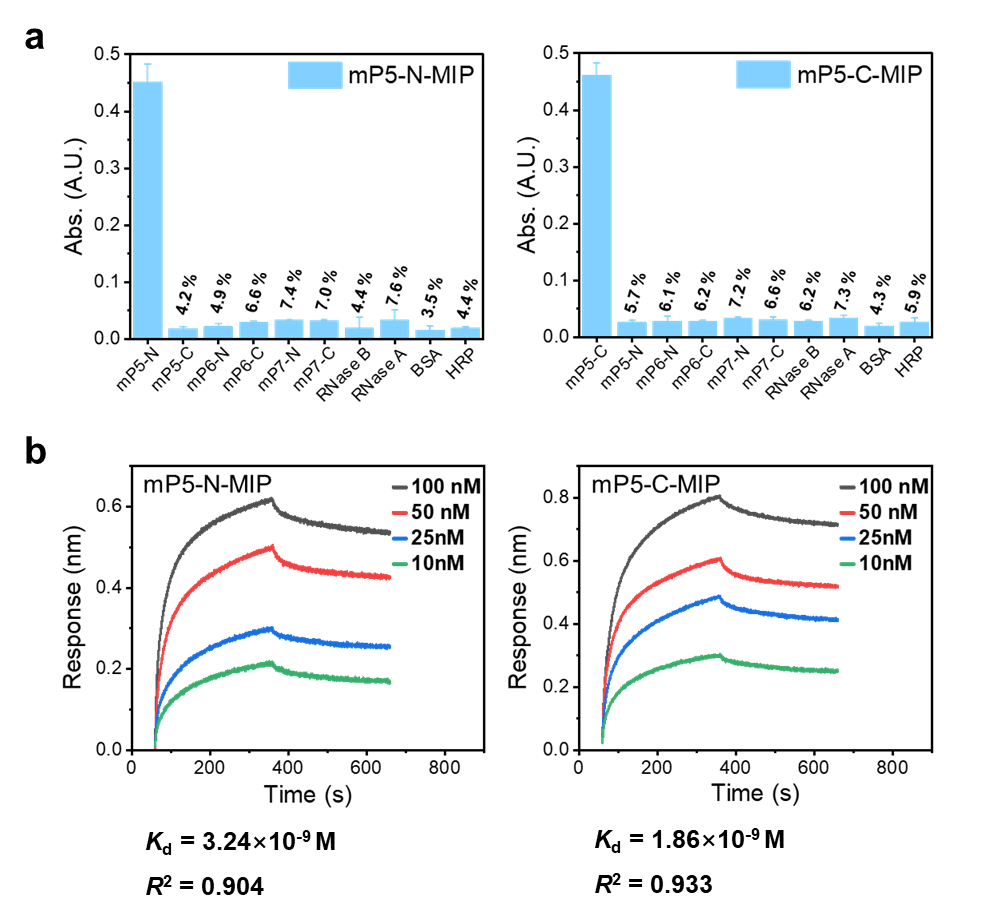
**

**Figure S9. Terminal epitope imprinting of mP5.** a. Selectivity of optimized mP5-N-MIP and mP5-C-MIP. b. BLI adsorption kinetics and dissociation constants of optimized mP5-N-MIP and mP5-C-MIP. Data represent mean ± SEM.  All experiments were performed in triplicate.

**
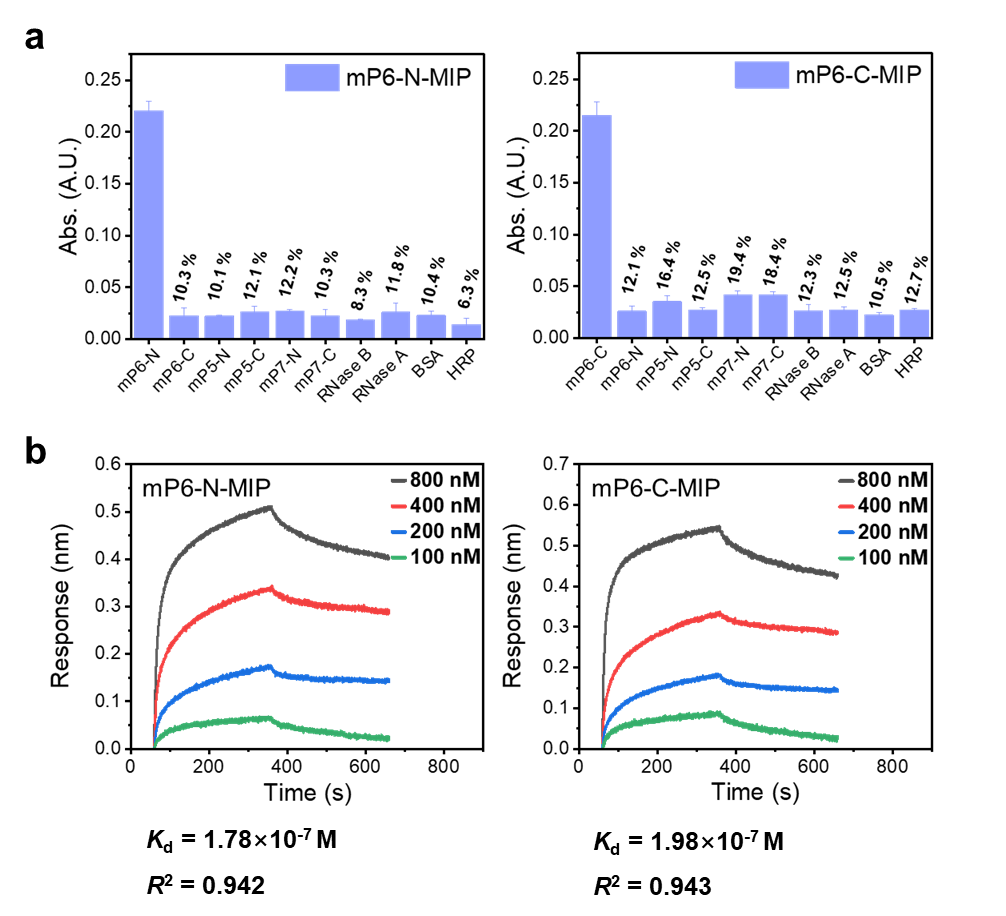
**

**Figure S10. Terminal epitope imprinting of mP6.** a. Selectivity of optimized mP6-N-MIP and mP6-C-MIP. b. BLI adsorption kinetics and dissociation constants of optimized mP6-N-MIP and mP6-C-MIP. Data represent mean ± SEM.  All experiments were performed in triplicate.

**
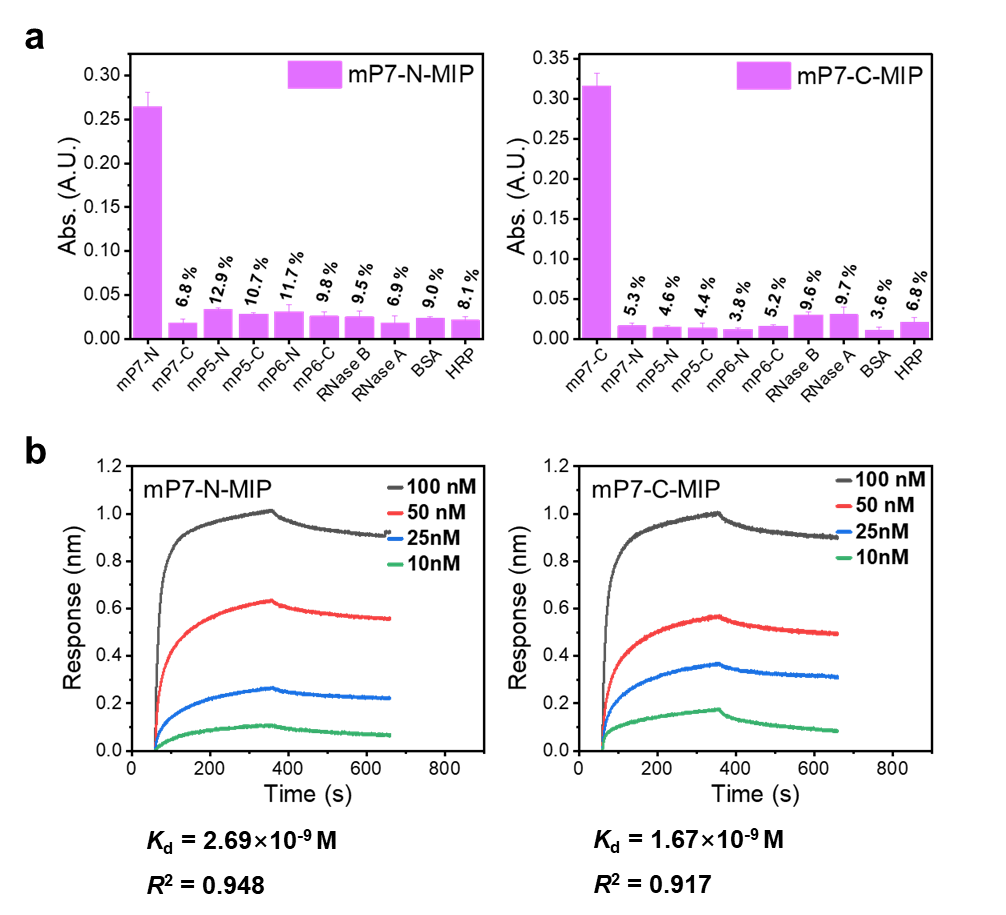
**

**Figure S11. Terminal epitope imprinting of mP7.** a. Selectivity of optimized mP7-N-MIP and mP7-C-MIP. b. BLI adsorption kinetics and dissociation constants of optimized mP7-N-MIP and mP7-C-MIP. Data represent mean ± SEM.  All experiments were performed in triplicate.

**
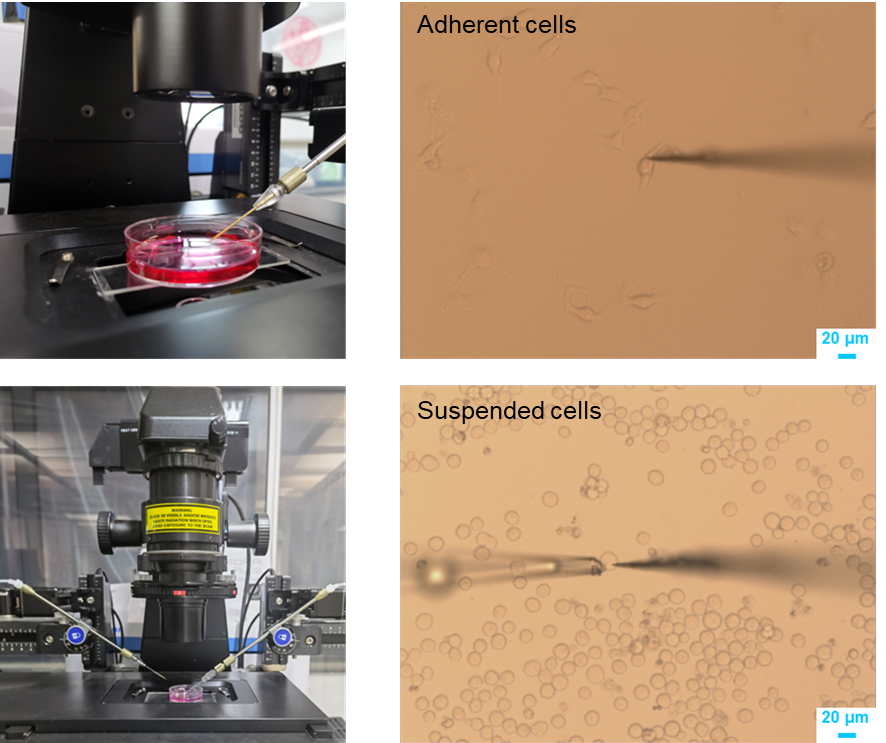
**

**Figure S12. Imaging of single cell analysis via a home-built 3D-micromanipulation for whether adherent or suspended living cells.**

**
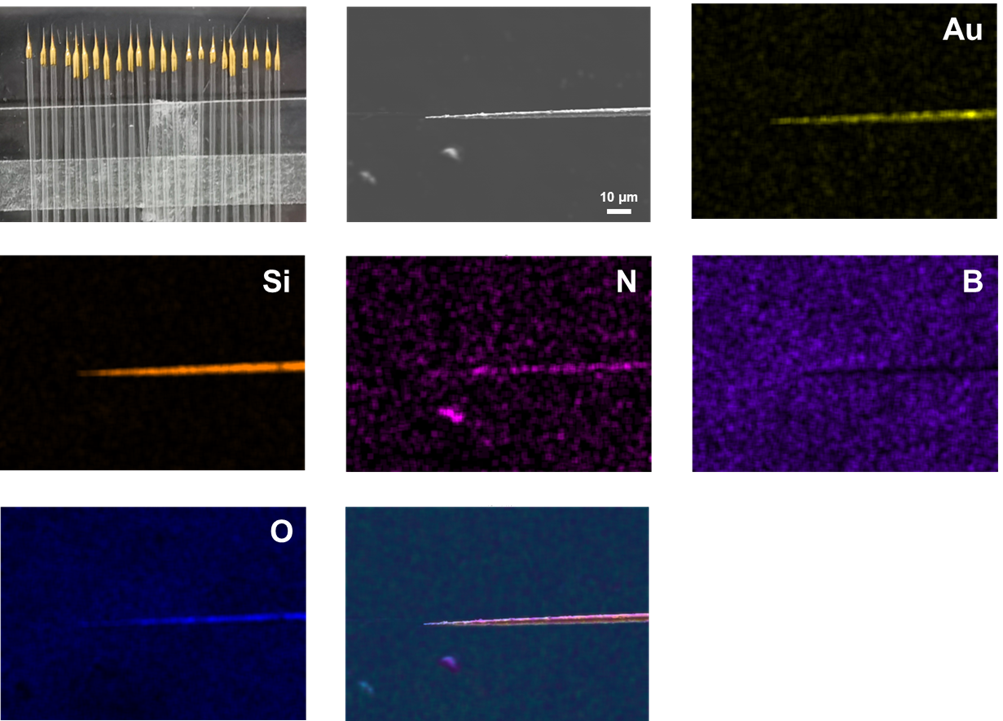
**

**Figure S13. Characterization of molecularly imprinted gold-based microprobe by photograph and scanning electron microscopy (SEM).**

**
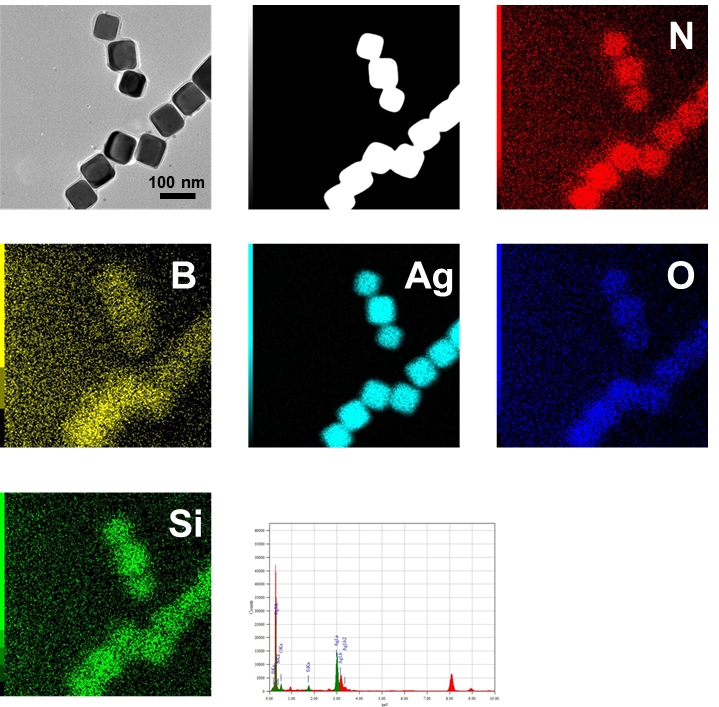
**

**Figure S14. Characterization of molecularly imprinted silver nanocube by transmission electron microscopy (TEM).**

**
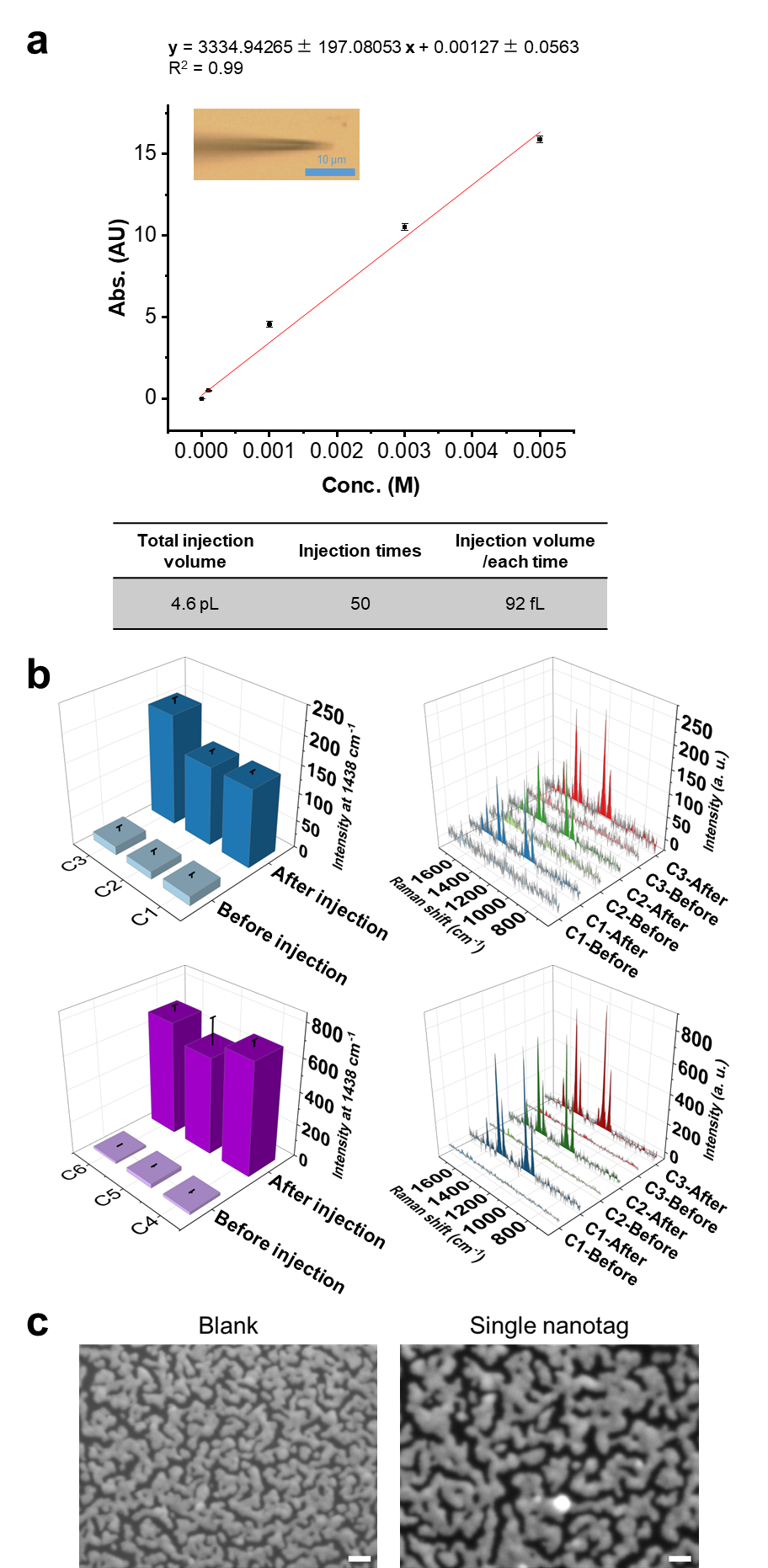
**

**Figure S15. Quantification of injection volume with dopamine as a marker.** a. quantitative evaluation of microinjection volume. b. single-molecule level detection after microinjection. c. SEM characterization of cube-on-probe type under single-molecule detection mode. Scale bar: 100 nm. Data represent mean ± SEM.  All experiments were performed in triplicate.

**
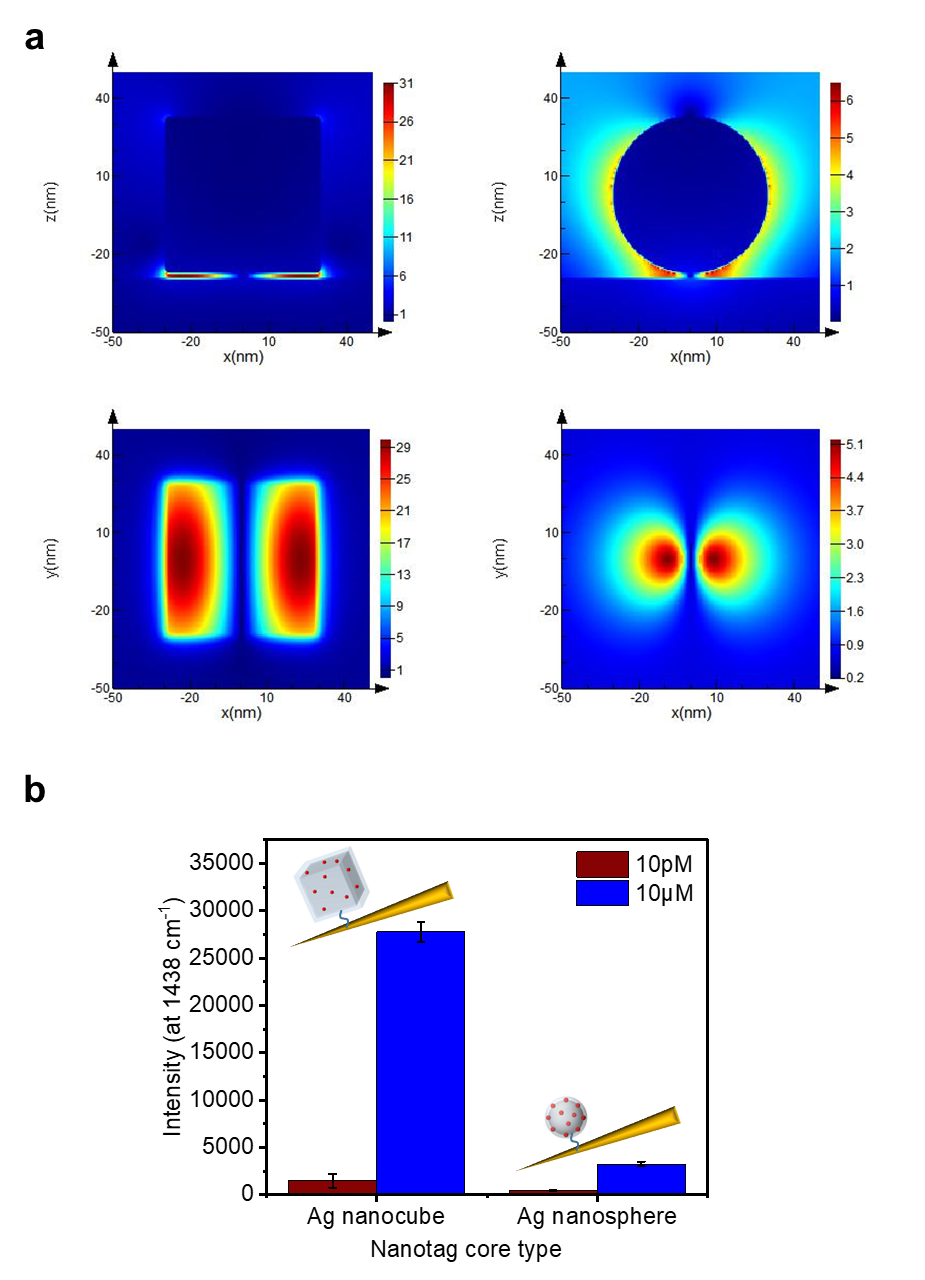
**

**Figure S16. Signal amplification performance of cube-on-probe type and particle-on-probe type nanostructure.** a. Comparison of electromagnetic enhancement effect by finite difference time domain (FDTD). b. Comparison of Raman signal enhancement using plasmonic cube-on-probe or sphere-on-probe type. Data represent mean ± SEM.  All experiments were performed in triplicate.

**
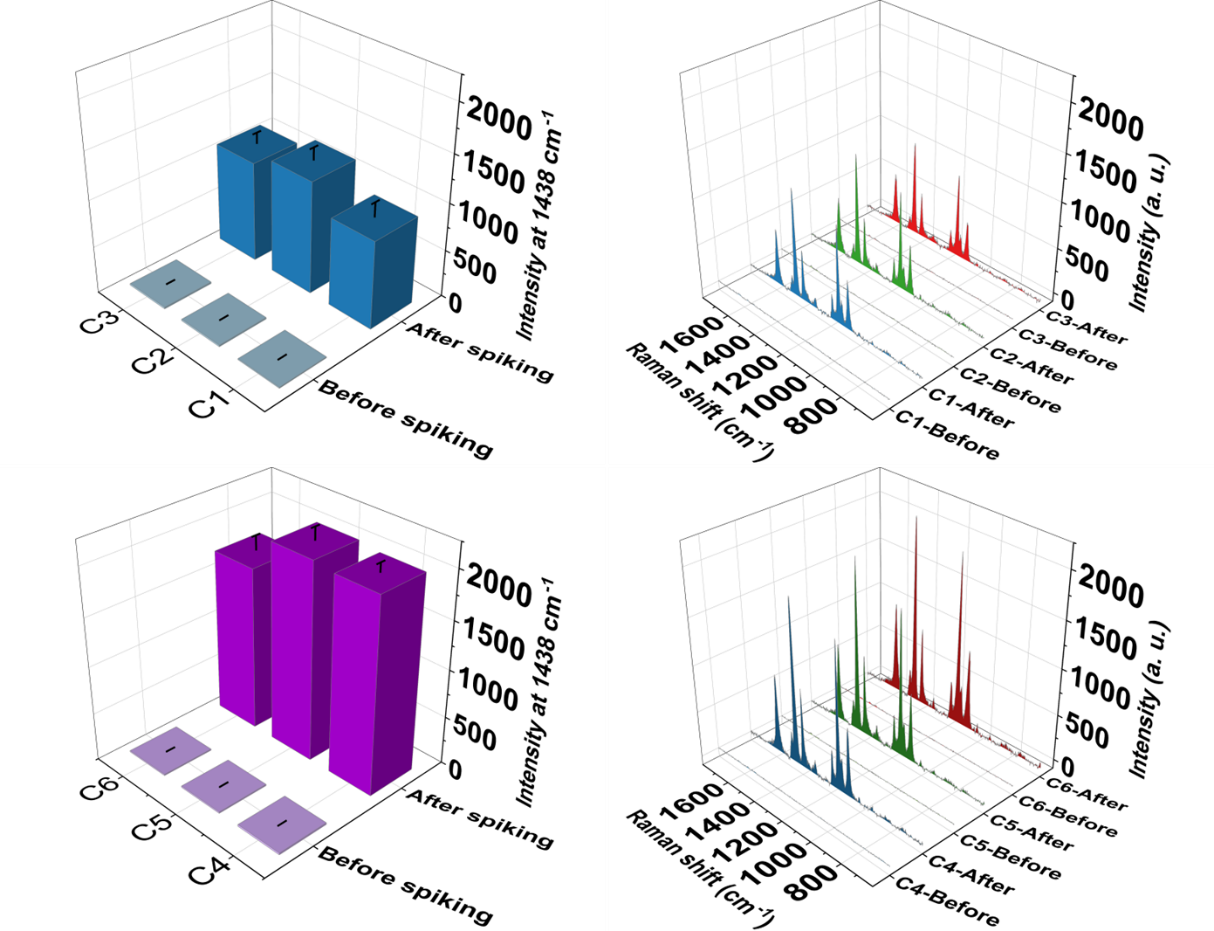
**

**Figure S17. Spike-and-test experiments of mP5 in single living MCF10A cells with spiking ~ 20 copies and ~ 200 copies, separately.** C1-C3, randomly selected single living cells for microinjection with ~ 20 copies. C4-C6, randomly selected single living cells for microinjection with ~ 200 copies. Data represent mean ± SEM.  All experiments were performed in triplicate.

**
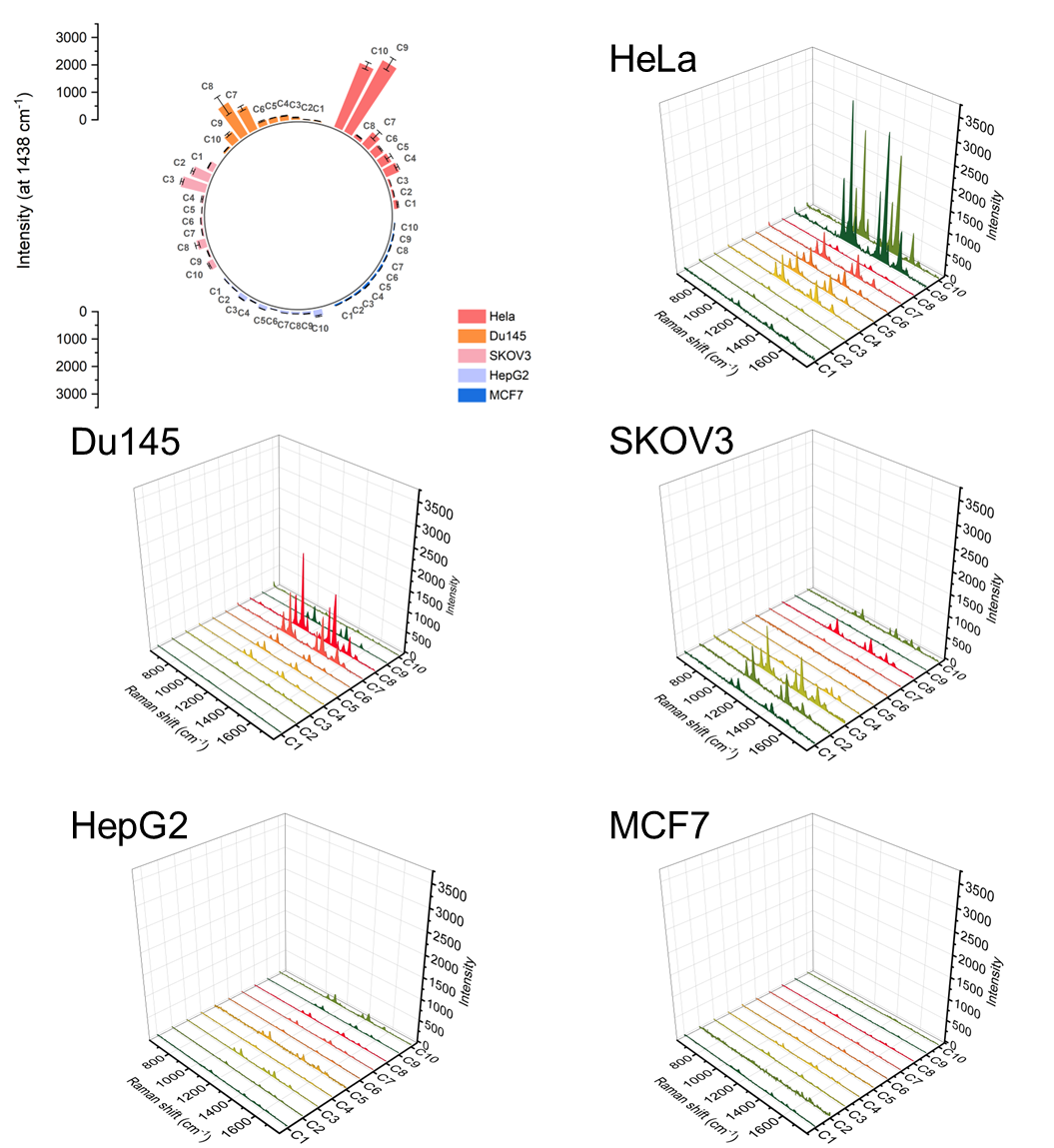
**

**Figure S18. Raman spectra and signal at 1438 cm^-1^ for detection of mP1 in single living cells using MIP-based PISA.** Data represent mean ± SEM.  All experiments were performed in triplicate.

**
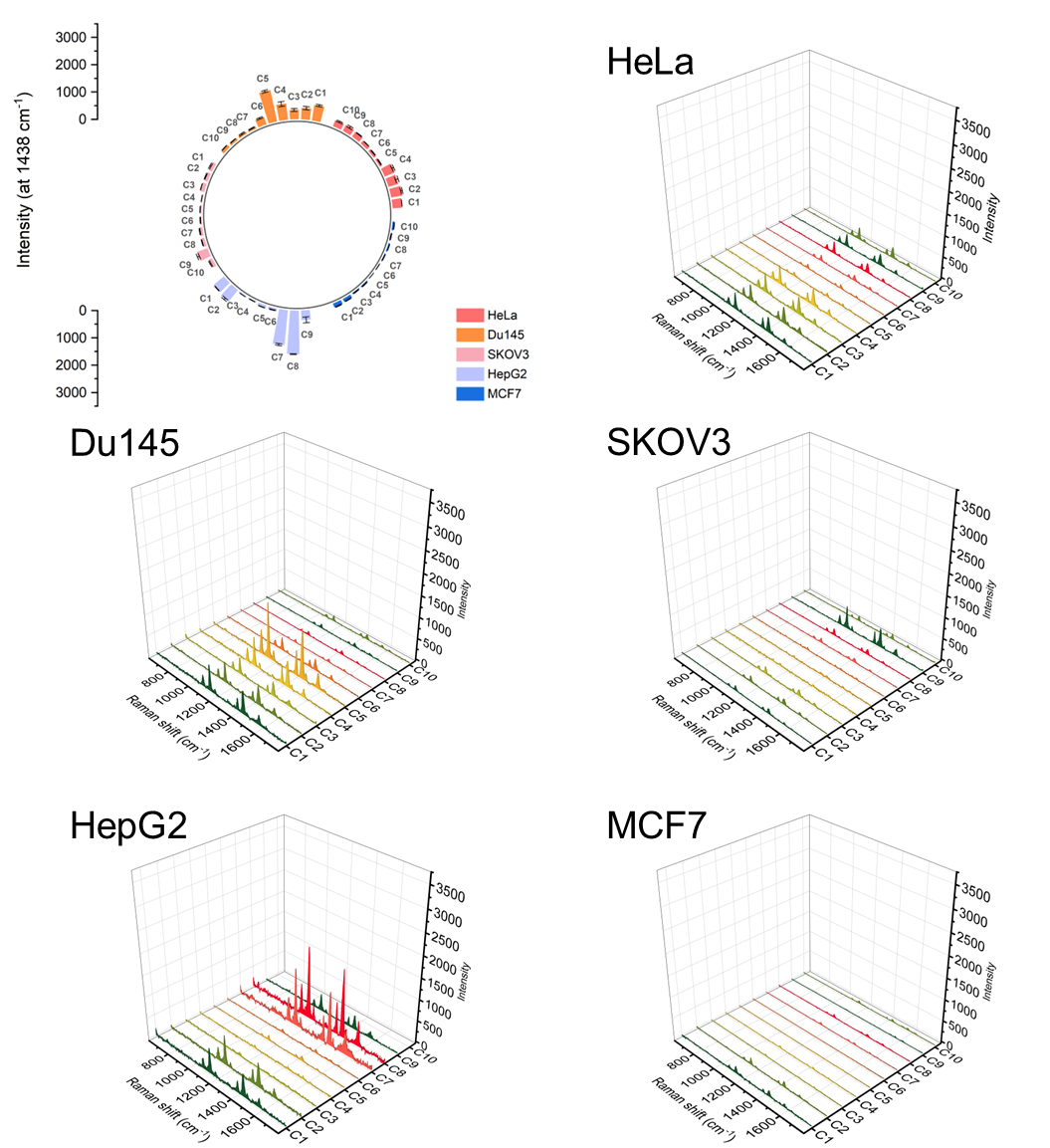
**

**Figure S19. Raman spectra and signal at 1438 cm^-1^ for detection of mP2 in single living cells using MIP-based PISA.** Data represent mean ± SEM.  All experiments were performed in triplicate.

**
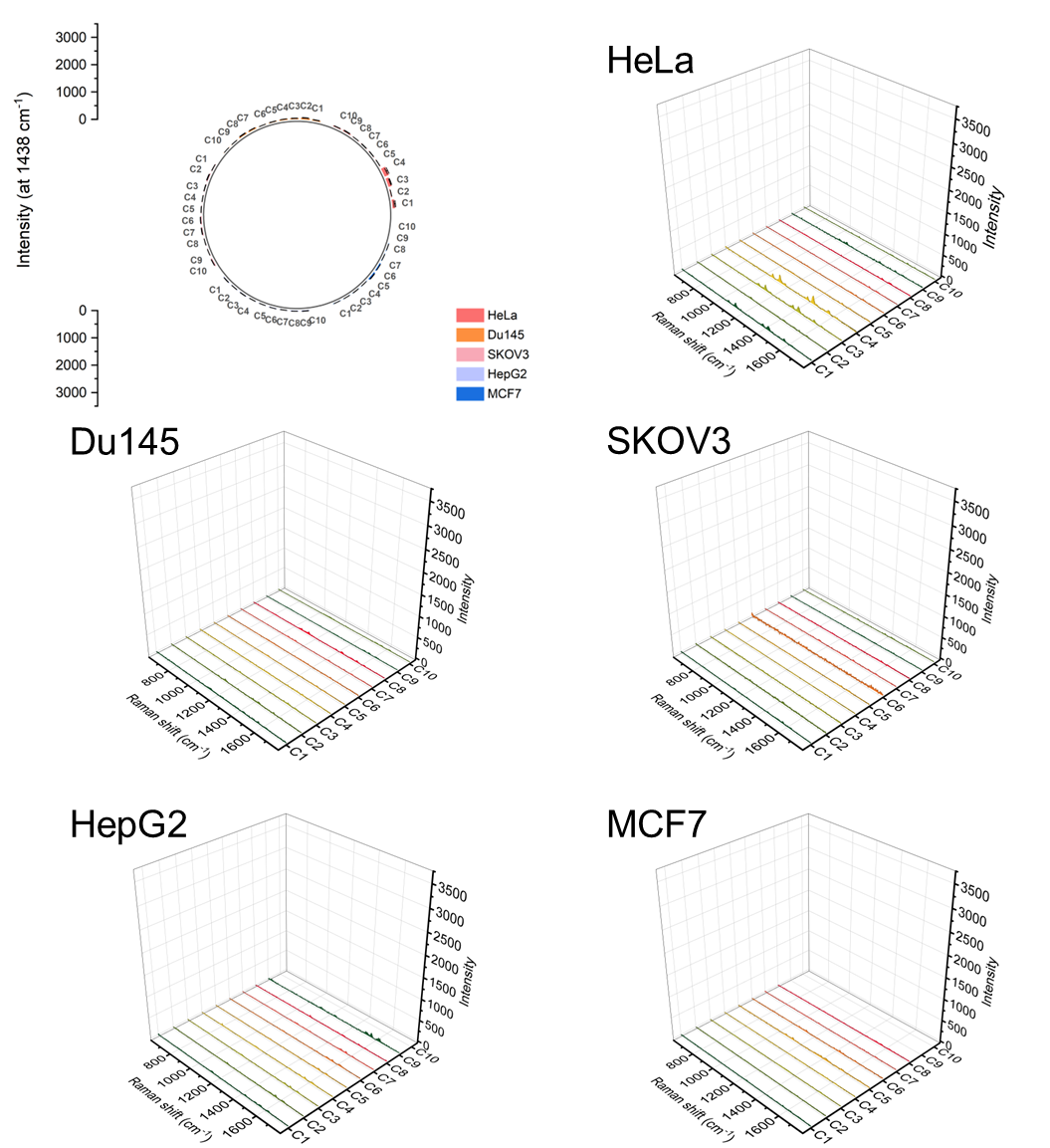
**

**Figure S20. Raman spectra and signal at 1438 cm^-1^ for detection of mP3 in single living cells using MIP-based PISA.** Data represent mean ± SEM.  All experiments were performed in triplicate.

**
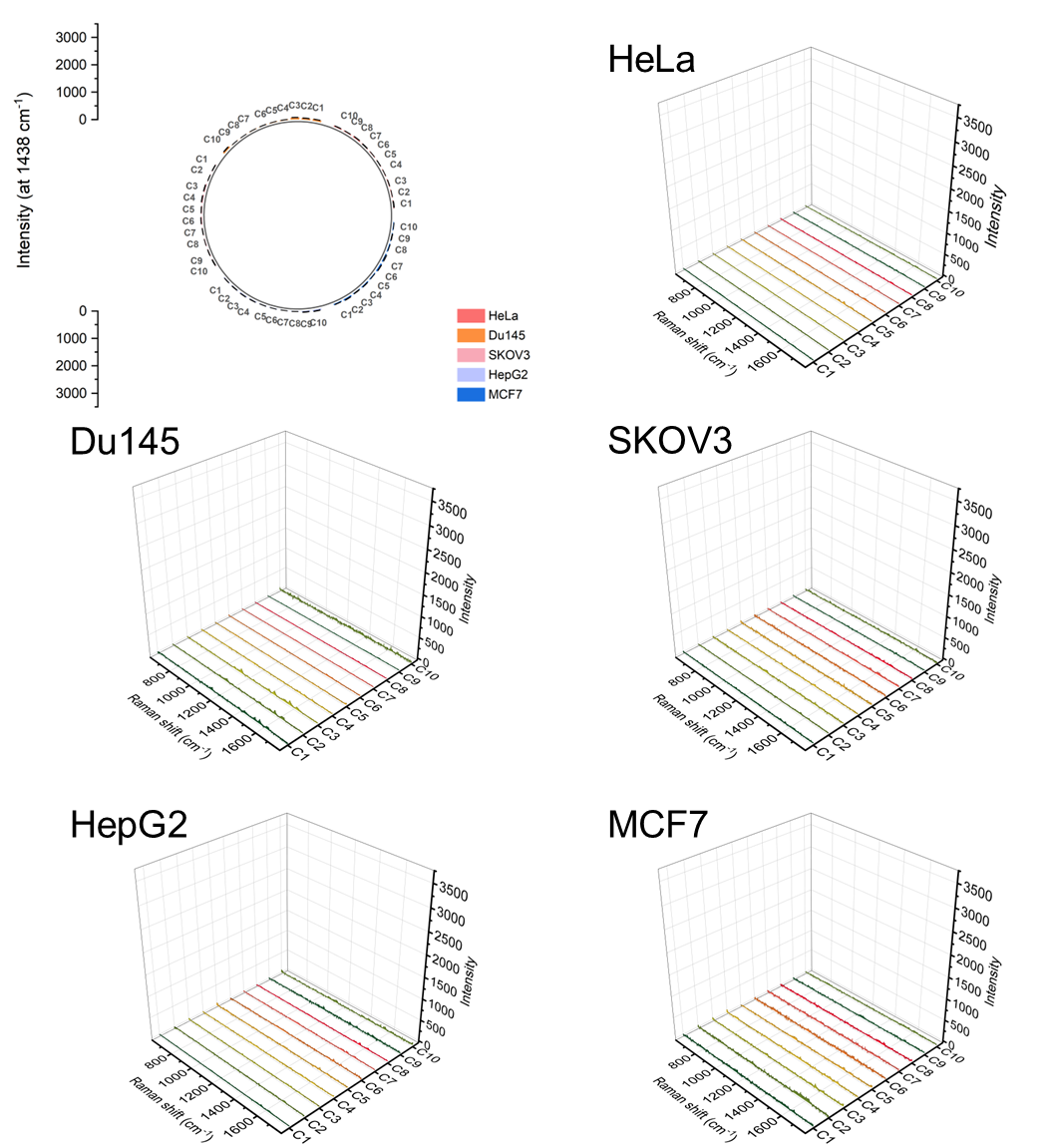
**

**Figure S21. Raman spectra and signal at 1438 cm^-1^ for detection of mP4 in single living cells using MIP-based PISA.** Data represent mean ± SEM.  All experiments were performed in triplicate.

**
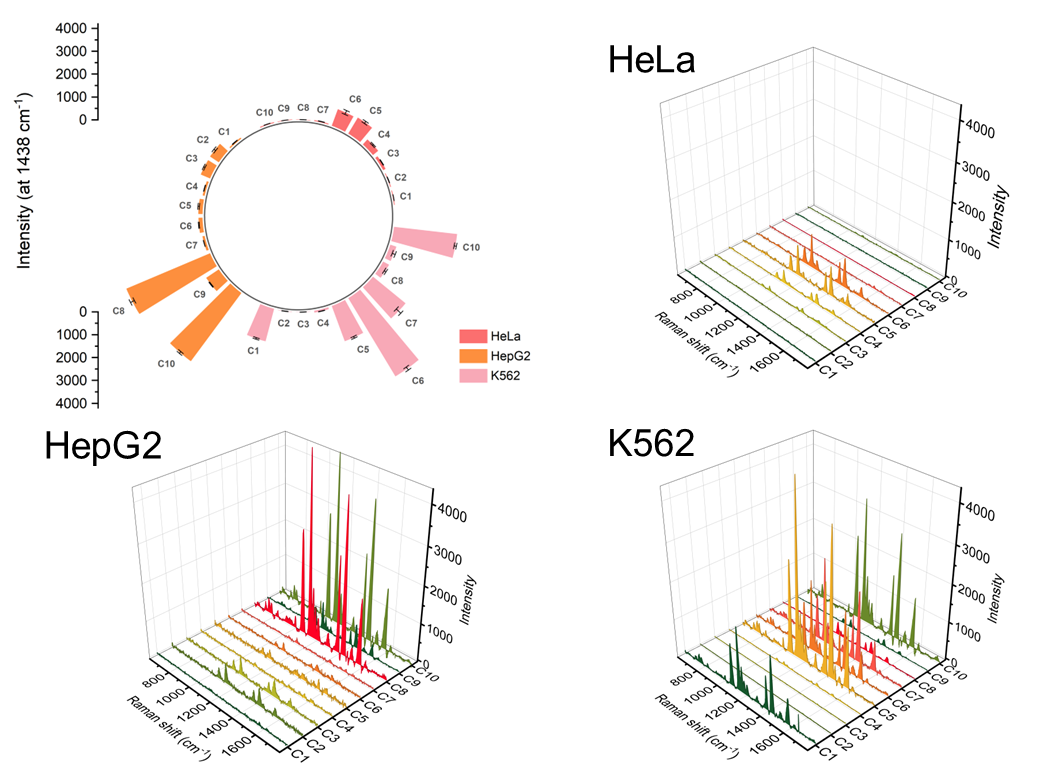
**

**Figure S22. Raman spectra and signal at 1438 cm^-1^ for detection of mP5 in single living cells using MIP-based PISA.** Data represent mean ± SEM.  All experiments were performed in triplicate.

**
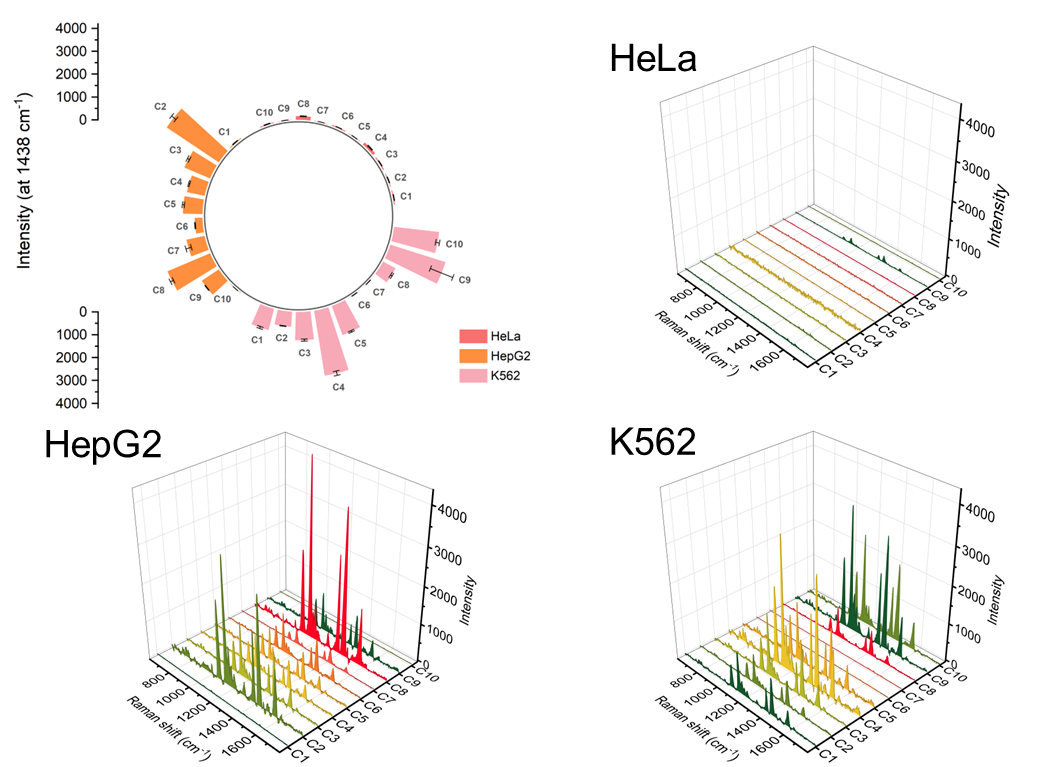
**

**Figure S23. Raman spectra and signal at 1438 cm^-1^ for detection of mP6 in single living cells using MIP-based PISA.** Data represent mean ± SEM.  All experiments were performed in triplicate.

**
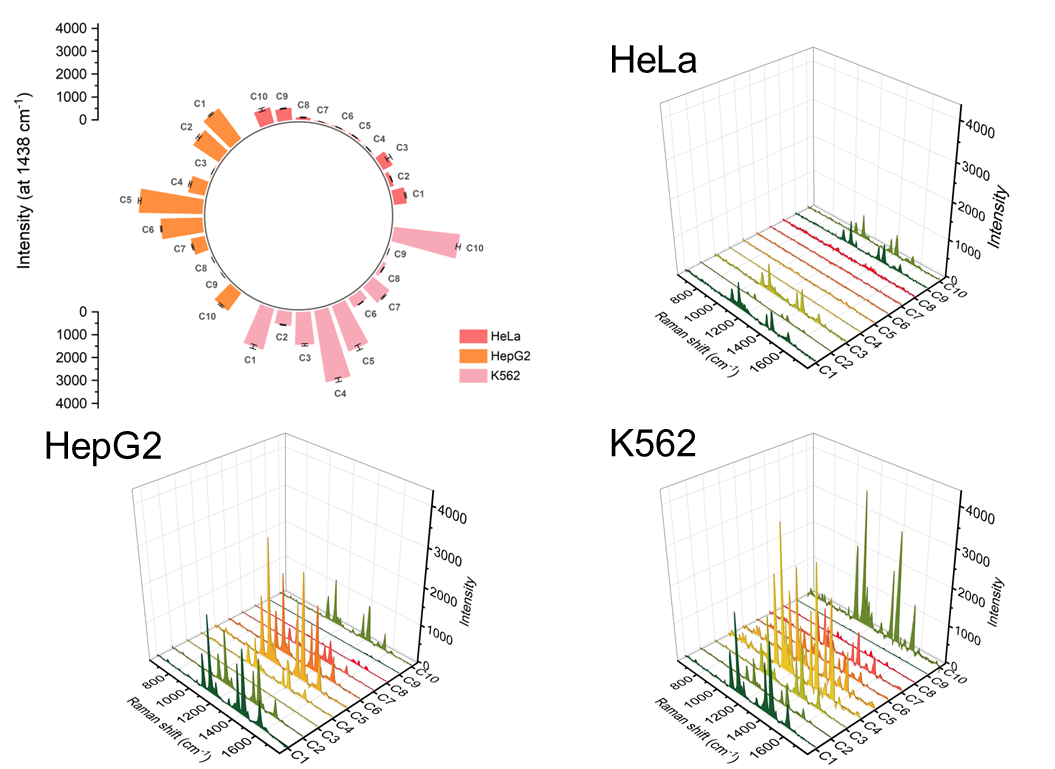
**

**Figure S24. Raman spectra and signal at 1438 cm^-1^ for detection of mP7 in single living cells using MIP-based PISA.** Data represent mean ± SEM.  All experiments were performed in triplicate.

**
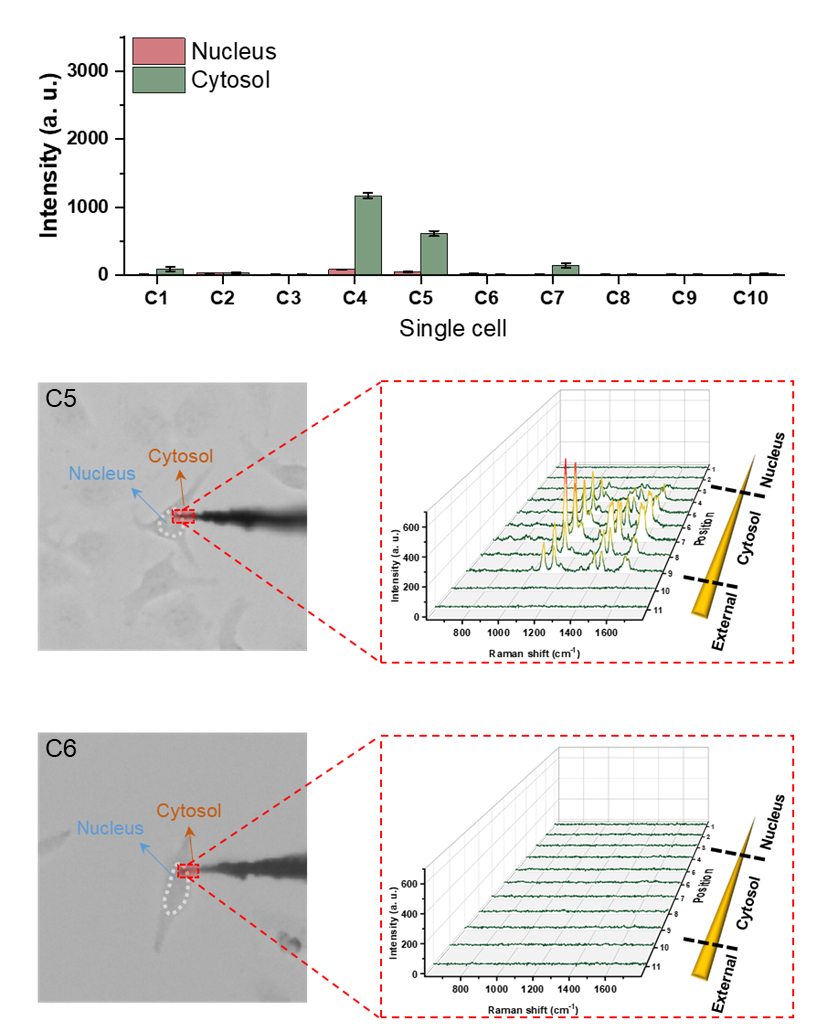
**

**Figure S25. Subcellular detection of mP5 in single living HepG2 cells.** Data represent mean ± SEM.  All experiments were performed in triplicate.


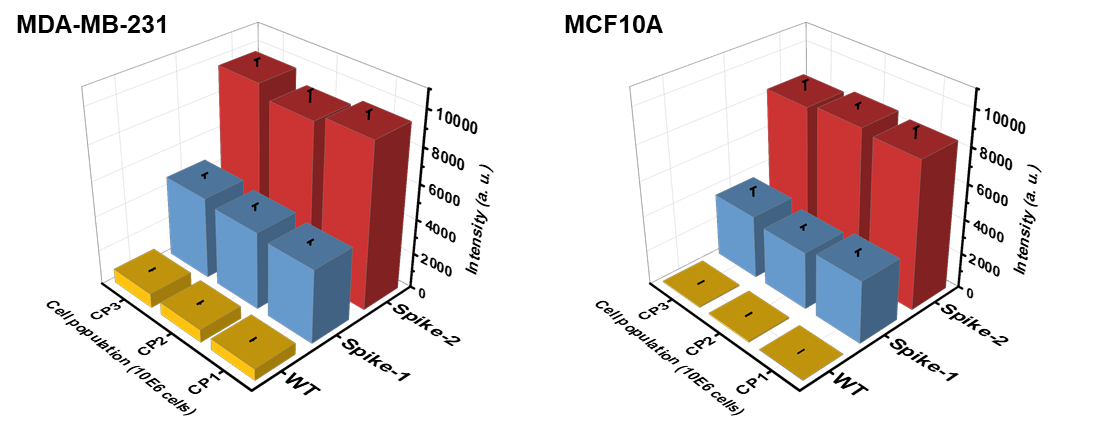


**Figure S26. Spike-and-test experiments of mP5 in cell populations.** WT: no spiking mP5, Spike-1: mP5 spiking concentration is 10 pg/mL, Spike-2: mP5 spiking concentration is 100 pg/mL. Data represent mean ± SEM.  All experiments were performed in triplicate.


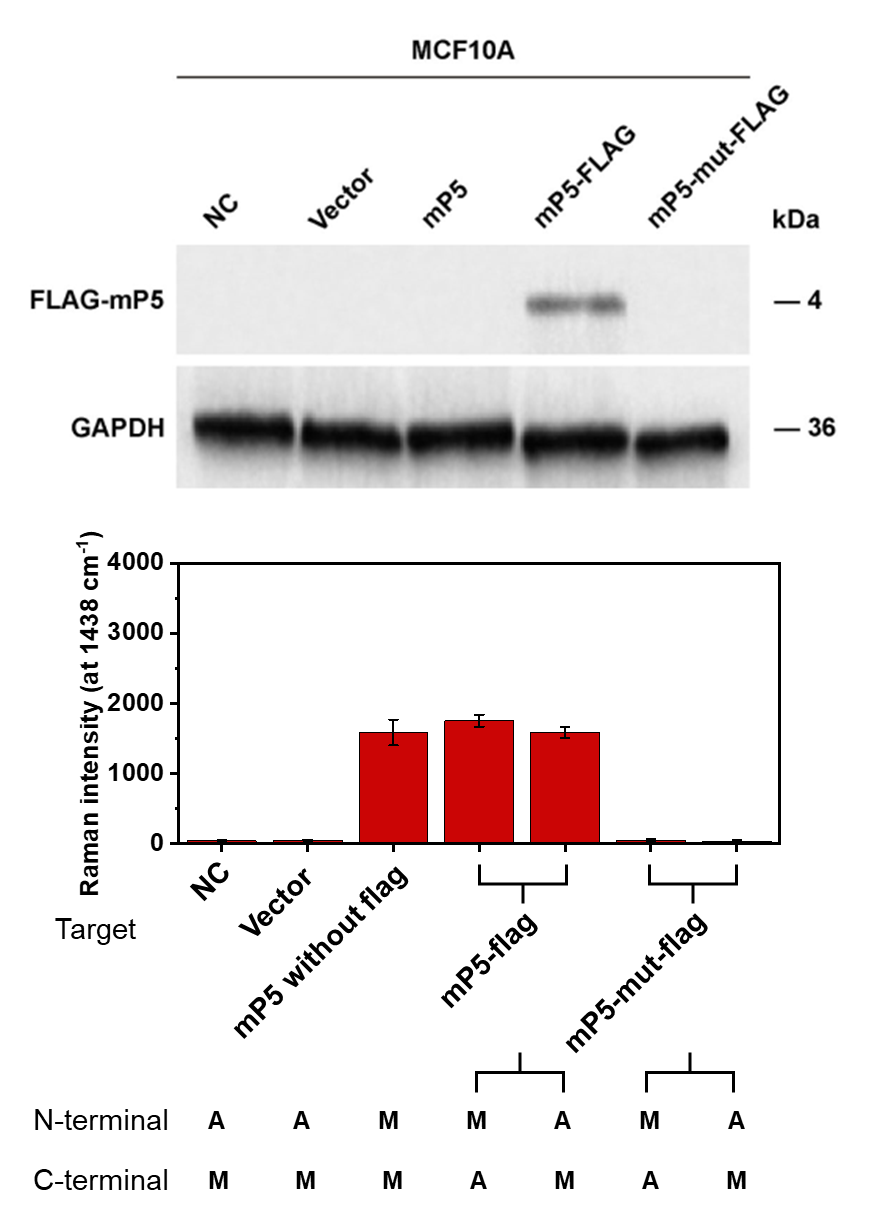


**Figure S27. Specificity validation by the comparison of WB and CLAIMID.** WB and CLAIMID for detection of overexpressed mP5, mP5-flag and mP5-mut-flag in MCF10A. A, anti-flag-based probe and SERS nanotag. M, MIP-based probe or SERS nanotag. Data represent mean ± SEM.  All experiments were performed in triplicate.

**
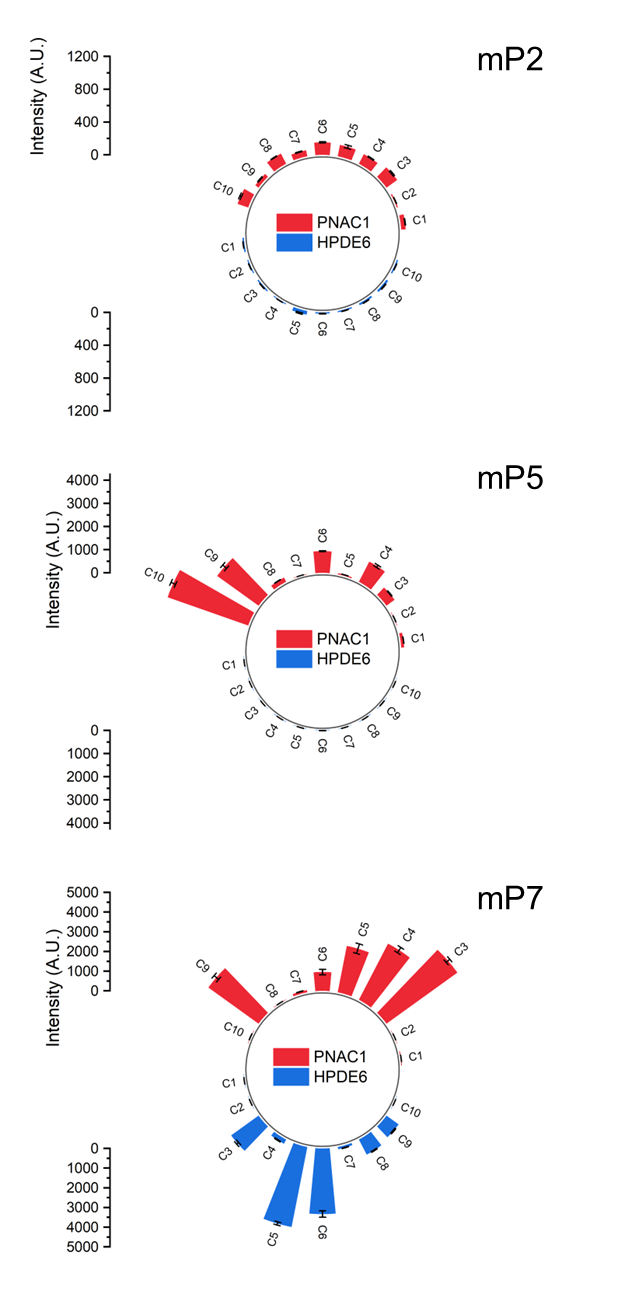
**

**Figure S28. Expression difference of mP2, mP5 and mP7 in single living pancreatic cells.** Data represent mean ± SEM.  All experiments were performed in triplicate.

**
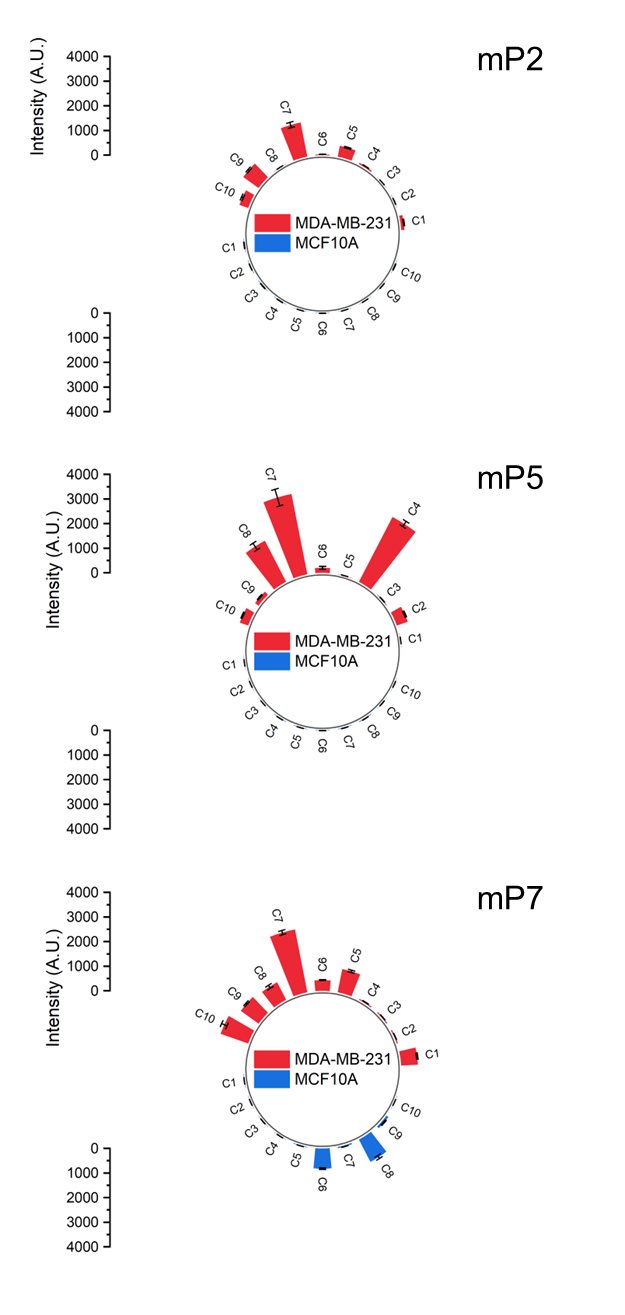
**

**Figure S29. Expression difference of mP2, mP5 and mP7 in single living mammary cells.** Data represent mean ± SEM.  All experiments were performed in triplicate.

**
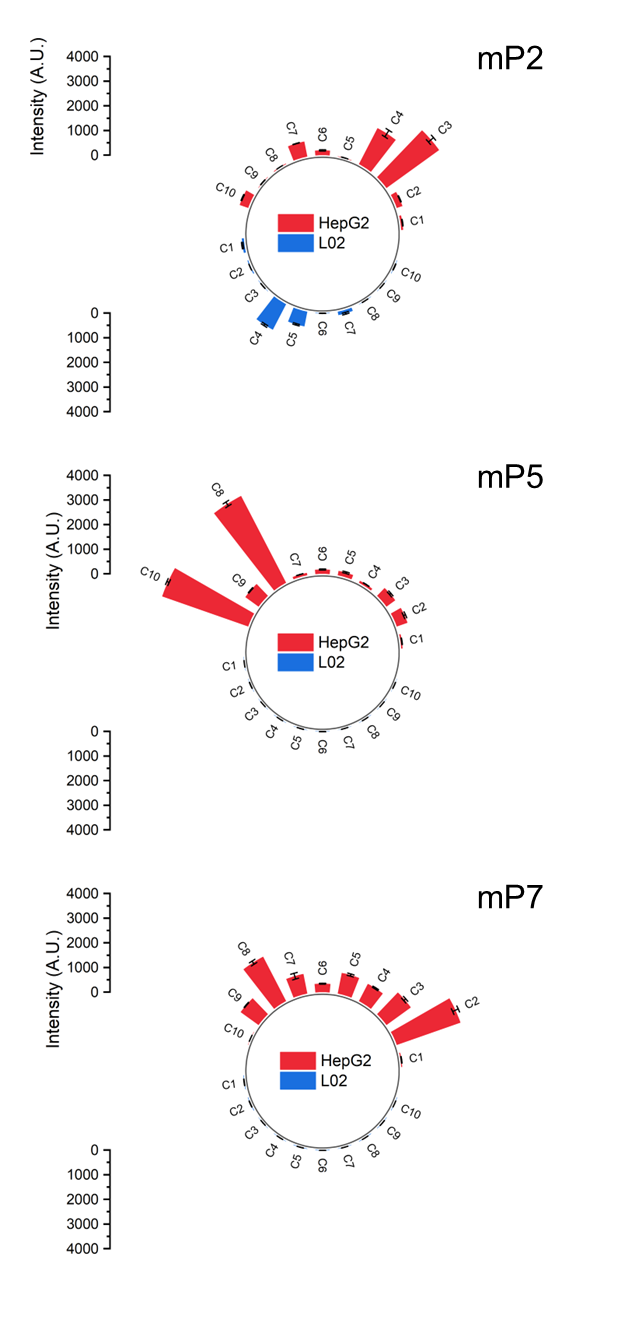
**

**Figure S30. Expression difference of mP2, mP5 and mP7 in single living hepatocytes.** Data represent mean ± SEM.  All experiments were performed in triplicate.

**
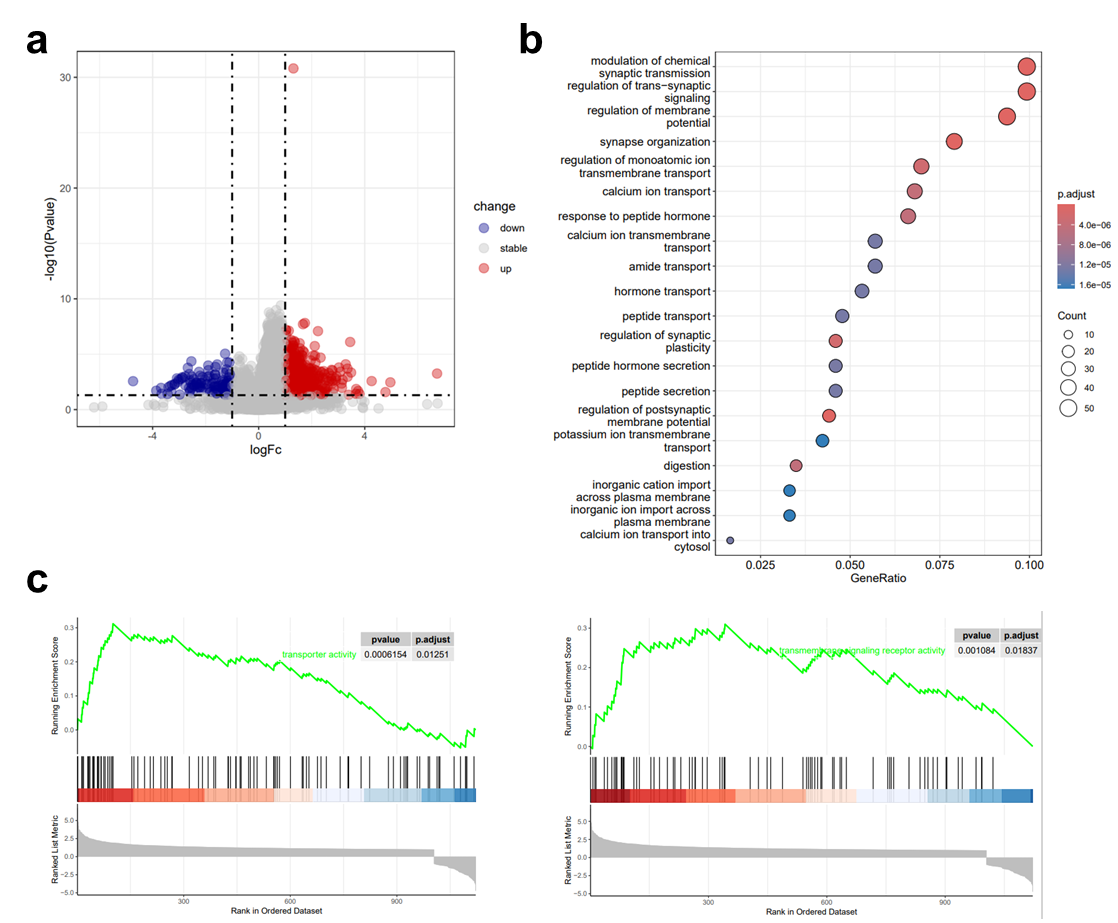
**

**Figure S31. Bioinformatic analysis for LINC00961.** a. Differentially expressed genes (DEGs) analysis of LINC00961 in clinical pancreatic cancer samples from TCGA (LINC00961 encoded mP2). b. Gene Ontology (GO) enrichment analysis for the DEGs. c. Gene set enrichment analysis (GSEA) for the DEGs.

**
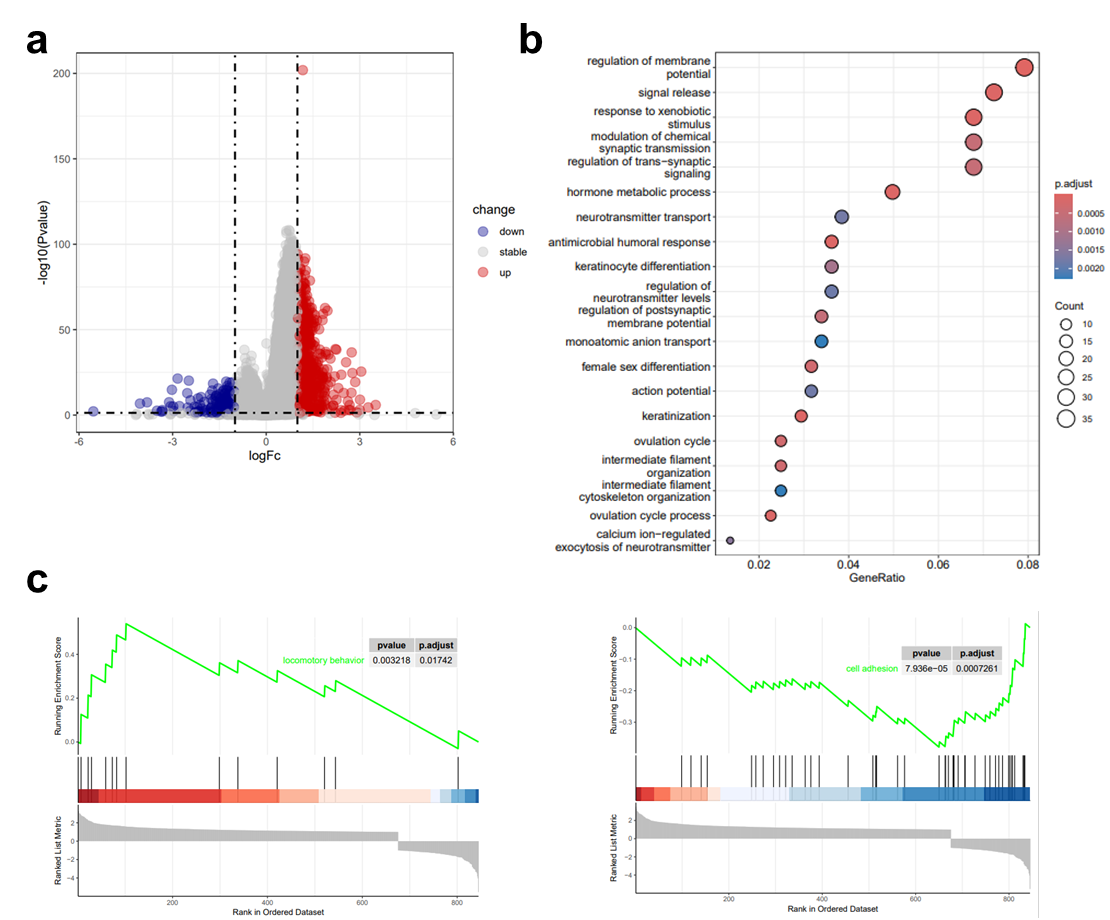
**

**Figure S32. Bioinformatic analysis for EBLN3P.** a. Differentially expressed genes (DEGs) of EBLN3P in clinical breast cancer samples from TCGA (EBLN3P encoded mP5). b. Gene Ontology (GO) enrichment analysis for the DEGs. c. Gene set enrichment analysis (GSEA) for the DEGs.

**
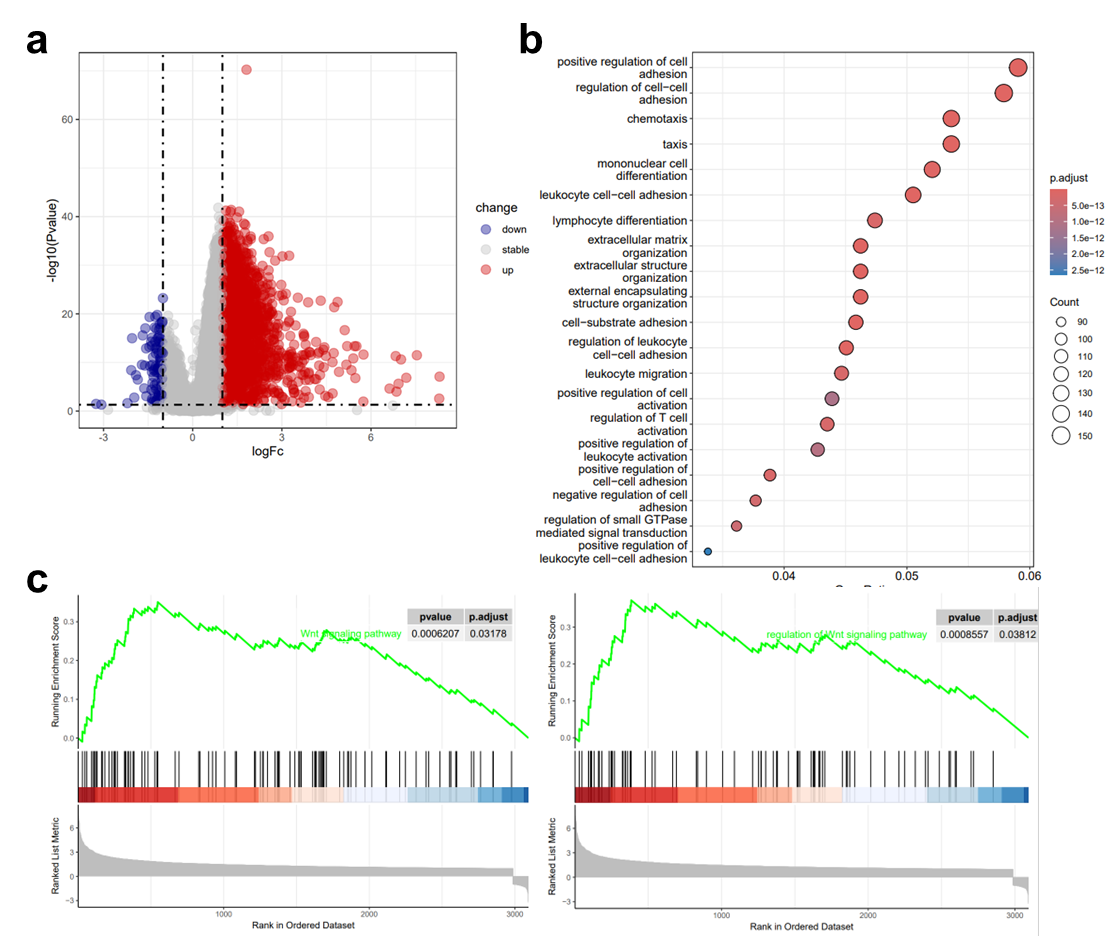
**

**Figure S33. Bioinformatic analysis for RNF145.** a. Differentially expressed genes (DEGs) of RNF145 in clinical liver cancer samples from TCGA (RNF145 encoded mP7). b. Gene Ontology (GO) enrichment analysis for the DEGs. c. Gene set enrichment analysis (GSEA) for the DEGs.

**References**

1. D. Lauressergues, J. M. Couzigou, H. S. Clemente, Y. Martinez, C. Dunand, G. Becard, J. P. Combier, *Nature* **2015**, *520* (7545), 90. <https://doi.org/10.1038/nature14346>
2. B. R. Nelson, C. A. Makarewich, D. M. Anderson, B. R. Winders, C. D. Troupes, F. Wu, A. L. Reese, J. R. McAnally, X. Chen, E. T. Kavalali, S. C. Cannon, S. R. Houser, R. Bassel-Duby, E. N. Olson, *Science* **2016**, *351* (6270), 271. <https://doi.org/10.1126/science.aad4076>
3. A. Matsumoto, A. Pasut, M. Matsumoto, R. Yamashita, J. Fung, E. Monteleone, A. Saghatelian, K. I. Nakayama, J. G. Clohessy, P. P. Pandolfi, *Nature* **2017**, *541* (7636), 228. <https://doi.org/10.1038/nature21034>
4. Q. Zhang, A. A. Vashisht, J. O'Rourke, S. Y. Corbel, R. Moran, A. Romero, L. Miraglia, J. Zhang, E. Durrant, C. Schmedt, S. C. Sampath, S. C. Sampath, *Nat Commun* **2017**, *8*, 15664. <https://doi.org/10.1038/ncomms15664>
5. D. M. Anderson, K. M. Anderson, C. L. Chang, C. A. Makarewich, B. R. Nelson, J. R. McAnally, P. Kasaragod, J. M. Shelton, J. Liou, R. Bassel-Duby, E. N. Olson, *Cell* **2015**, *160* (4), 595. <https://doi.org/10.1016/j.cell.2015.01.009>
6. P. Bi, A. Ramirez-Martinez, H. Li, J. Cannavino, J. R. McAnally, J. M. Shelton, E. Sanchez-Ortiz, R. Bassel-Duby, E. N. Olson, *Science* **2017**, *356* (6335), 323. <https://doi.org/10.1126/science.aam9361>
7. J. Chen, A. D. Brunner, J. Z. Cogan, J. K. Nunez, A. P. Fields, B. Adamson, D. N. Itzhak, J. Y. Li, M. Mann, M. D. Leonetti, J. S. Weissman, *Science* **2020**, *367* (6482), 1140. <https://doi.org/10.1126/science.aay0262>
8. R. Jackson, L. Kroehling, A. Khitun, W. Bailis, A. Jarret, A. G. York, O. M. Khan, J. R. Brewer, M. H. Skadow, C. Duizer, C. C. D. Harman, L. Chang, P. Bielecki, A. G. Solis, H. R. Steach, S. Slavoff, R. A. Flavell, *Nature* **2018**, *564* (7736), 434. <https://doi.org/10.1038/s41586-018-0794-7>
9. S. Zhu, J. Z. Wang, Chen, Y. T. He, N. Meng, M. Chen, R. X. Lu, X. H. Chen, X. L. Zhang, G. R. Yan, *Nat Commun* **2020**, *11* (1), 1685. <https://doi.org/10.1038/s41467-020-15403-9>
10. Q. Li, G. Guo, Y. Chen, L. Lu, H. Li, Z. Zhou, J. Guo, X. Gan, Y. Hu, Q. Li, M. Sun, X. Liu, *Adv Sci* **2024**, *11* (46), e2407012. <https://doi.org/10.1002/advs.202407012>
11. T. F. Martinez, Q. Chu, C. Donaldson, D. Tan, M. N. Shokhirev, A. Saghatelian, *Nat Chem Biol* **2020**, *16* (4), 458. <https://doi.org/10.1038/s41589-019-0425-0>
12. X. Cao, A. Khitun, Y. Luo, Z. Na, T. Phoodokmai, K. Sappakhaw, E. Olatunji, C. Uttamapinant, S. A. Slavoff, *Nat Commun* **2021**, *12* (1), 508. <https://doi.org/10.1038/s41467-020-20841-6>
13. A. L. Rocha, C. Schmedt, G. Perkins, A. Pinto, J. K. Diedrich, H. Shan, K. Plucinska, E. Vieira de Souza, J. M. Vaughan, M. Foster, S. C. Sampath, S. C. Sampath, P. Cohen, M. H. Ellisman, A. Saghatelian, *Sci Adv* **2025**, *11* (35), eads7381. <https://doi.org/10.1126/sciadv.ads7381>

((Please insert your Supporting Information text/figures here. Please note: Supporting Display items, should be referred to as Figure S1, Equation S2, etc., in the main text…)
